# Training Gaps in Digital Skills for the Cancer Health Care Workforce Based on Insights From Clinical Professionals, Nonclinical Professionals, and Patients and Caregivers: Qualitative Study

**DOI:** 10.2196/78490

**Published:** 2025-10-08

**Authors:** David Liñares, Theologia Tsitsi, Noemí López-Rey, Wilfredo Guanipa-Sierra, Susana Aldecoa-Landesa, Carme Carrión, Daniela Cabutto, Deborah Moreno-Alonso, Clara Madrid-Alejos, Andreas Charalambous, Ana Clavería

**Affiliations:** 1 Galician Agency for Health Technology Assessment Avalia-T. Galician Agency for Health Knowledge Management Santiago Spain; 2 I-Saúde Group Galicia Sur Health Research Institute (IIS Galicia Sur) Servicio Galego de Saúde-Universidade de Vigo Vigo Spain; 3 Research Network in Chronicity, Primary Care and Health Promotion (Red de Investigación en Cronicidad, Atención Primaria y Promoción de la Salud) Zaragoza Spain; 4 Department of Nursing Cyprus University of Technology Limassol Cyprus; 5 Health Center CIS-Milagrosa (Servicio Galego de Saúde) Lugo Spain; 6 Health Center Beiramar (Servicio Galego de Saúde) Vigo Spain; 7 eHealth Lab Research Group School of Health Sciences Universitat Oberta de Catalunya (UOC). Barcelona Spain; 8 e-Oncologia and Cancer Education Research Unit Institut Català d'Oncologia (ICO) Barcelona Spain; 9 Department of Nursing University of Turku Turku Finland; 10 Área de Vigo, Servicio Galego de Saúde Vigo Spain

**Keywords:** health literacy, professional competence, health personnel, telemedicine, oncology nursing

## Abstract

**Background:**

The integration of digital technologies is becoming increasingly essential in cancer care. However, limited digital health literacy among clinical and nonclinical cancer health care professionals poses significant challenges to effective implementation and sustainability over time. To address this, the European Union is prioritizing the development of targeted digital skills training programs for cancer care providers, the TRANSiTION project among them. A crucial initial step in this effort is conducting a comprehensive gap analysis to identify specific training needs.

**Objective:**

The aim of this work is to identify training gaps and prioritize the digital skill development needs in the oncology health care workforce.

**Methods:**

An importance-performance analysis (IPA) was conducted following a survey that assessed the performance and importance of 7 digital skills: information, communication, content creation, safety, eHealth problem-solving, ethics, and patient empowerment.

**Results:**

A total of 67 participants from 11 European countries completed the study: 38 clinical professionals (CP), 16 nonclinical professionals (NCP), and 13 patients or caregivers (PC). CP acknowledged the need for a comprehensive training program that includes all 7 digital skills. Digital patient empowerment and safety skills emerge as the highest priorities for both CP and NCP. Conversely, NCP assigned a lower priority to digital content creation skills, and PC assigned a lower priority to digital information and ethical skills. The IPA also revealed discrepancies in digital communication skills across groups (*H*=6.50; *P*=.04).

**Conclusions:**

The study showcased the pressing need for comprehensive digital skill training for cancer health care professionals across diverse backgrounds and health care systems in Europe, tailored to their occupation and care setting. Incorporating PC perspectives ensures a balanced approach to addressing these training gaps. These findings provide a valuable knowledge base for designing digital skills training programs, promoting a holistic approach that integrates the perspectives of the various stakeholders involved in digital cancer care.

## Introduction

### Background

Cancer is the second leading cause of premature mortality and morbidity worldwide [1]. In the European Union, nearly 4.7 million new cases of cancer and 2.1 million cancer-related deaths occur each year [2]. According to the European Commission, the urgency to address cancer control and outcomes is a significant political challenge, as reflected in Europe’s Beating Cancer Plan [3], with cancer being one of the 5 missions included in the Horizon Europe program.

Health literacy (HL), defined as the individual capacity to access, understand, evaluate, and apply health information to make informed health decisions [4]**,** is widely recognized as a critical factor in effective cancer care [5,6]. With the increasing integration of digital technologies, such as symptom monitoring platforms, treatment adherence tools, telehealth, and mobile apps, in oncology, HL has evolved to encompass the digital environment, giving rise to the concept of digital health literacy (DHL) [7-11]. DHL has been defined in various ways, reflecting the evolving nature of health information environments. Broadly, DHL refers to the ability to seek, find, understand, and appraise health information from electronic sources and to apply this knowledge to solve a health problem [12,13]. However, there is ongoing debate about its key attributes, particularly the relative weight of technical skills, critical thinking, health knowledge, and digital engagement, in shaping a comprehensive definition [14,15]. In response, recent studies have focused on 4 major areas: (1) conceptualizing and measuring DHL; (2) identifying and addressing the digital divide; (3) exploring the factors that influence DHL development; and (4) examining the health outcomes associated with DHL levels [16]. Regarding the latter, DHL enhances access to and quality of health care to the extent of being considered a “super determinant” of health, a factor with a profound impact across various health outcomes [17,18].

In cancer care, DHL is particularly relevant, enabling health care professionals and patients to benefit from digital innovations such as electronic health records, patient portals, symptom tracking tools, and remote care services [19]. Low levels of DHL have been associated with poorer clinical outcomes, including reduced overall survival among patients with cancer [20-22]. Limited DHL not only hinders patients and caregivers (PC) but also poses challenges for health care professionals, potentially impeding the effective adoption of digital health solutions in clinical practice [23]. The Towards European Health Data Space (TEHDAS) project highlights significant disparities in the health system infrastructures across European countries, with not all of them being adequately equipped to ensure effective management of digital health [24]. Still, the main barriers to implementing digital health strategies in health care organizations are not technical issues (such as infrastructure or connectivity). Instead, they are rooted in gaps in digital skills among professionals and patients, concerns about data security and confidentiality in digital environments, and limited time availability [25-28].

As a result of these challenges, experts in the field emphasize the need to develop flexible and easily accessible training programs, such as online modules and hands-on learning approaches, supported by appropriate incentives to engage and retain the oncology workforce [29]. Nevertheless, the results of DigiCanTRain, a European-cofunded project under the EU4Health Programme (2021-2027) of approximately €1.98 million (US $2.32 million), highlight significant challenges in the implementation of digital skills training programs for cancer professionals across 25 EU countries. These challenges include a lack of coordination between national and international organizations in promoting training initiatives, as well as limited access to continuous accreditation mechanisms that ensure the quality and consistency of educational content [30]. In response to these gaps, DigiCanTRain aims to design, pilot, and evaluate a comprehensive digital skills training curriculum for both clinical and nonclinical oncology professionals, with the goal of enhancing the adoption of eHealth technologies and fostering more person-centered, efficient, and resilient cancer care.

Despite these initiatives, international continuing education programs fail to identify the specific digital skills required by health care professionals [31]. Even if DigComp 2.2: The Digital Competence Framework for Citizens provides a reference framework for the global population on existing digital competencies [32], it does not include specific competencies oriented to health care professionals in cancer care, such as ethical or patient empowerment skills [33,34]. Moreover, there are no validated and widely used measurement tools available to assess eHealth competencies [35,36]. Therefore, the review by Tinmaz et al [37] highlighted the need to create updated digital frameworks for different work settings, professional categories, and contexts. In the context of cancer care, the study by Leena et al [34] revealed that the digital skills of health care professionals are multifaceted. Consequently, the authors indicated that it is imperative that these skills be subjected to a process of assessment to facilitate the provision of training that is based on the actual learning needs of the professionals in question.

In view of this, the TRANSiTION project [38] was cofunded at 80% by the European Union with a total budget of €2,299,541.28 (US $2,690,371). The project aims to design an advanced training program for both clinical professionals (CP) and nonclinical professionals (NCP) involved in cancer care, equipping them with essential digital skills to enhance the efficiency and effectiveness of information exchange with patients and other health care providers. TRANSiTION brings together an interdisciplinary consortium of 24 partners from 14 member states, all with extensive experience in the development, evaluation, and successful implementation of continuing professional development and training programs in oncology.

### Theoretical Framework

According to the European Commission, the development of a digital skills training program should be preceded by a thorough analysis of training needs and existing gaps [39]. A needs analysis is a systematic process used to identify discrepancies between the current and ideal states of an organization or service [40].

One widely adopted and intuitive method for conducting such an analysis is importance-performance analysis (IPA). Originally developed by Martilla and James [41], IPA provides a visual and analytical framework to support strategic decision-making by comparing the importance of specific attributes to their perceived performance. It has been extensively applied across diverse sectors, including IT services, marketing, banking, tourism, and sports [42]. More recently, IPA has been used in the assessment of training needs [43,44], process improvement [45], and evaluation of health care services [46].

IPA is based on a 2D grid that plots attributes according to their mean scores on 2 axes: (1) importance, which is the value or relevance assigned to the attribute by users, and (2) performance, which is the perceived effectiveness or quality of that attribute.

The axes of the IPA grid are determined by the overall mean scores of importance and performance across all attributes assessed. The position of each attribute within the grid reflects its scores on these 2 dimensions. This allows for a relative comparison, enabling the identification of attributes that deviate from the general trend.

The resulting matrix is divided into 4 quadrants, each linked to distinct action strategies [47]: Quadrant I—Focus here: high importance, low performance; these are critical areas in need of immediate improvement. Quadrant II—Keep up the good work: high importance, high performance; these are strengths to be preserved. Quadrant III—Low priority: low importance, low performance; these areas require minimal attention as they are not strategically significant. Quadrant IV—Possible overkill: low importance, high performance; these attributes may be receiving more resources than necessary.

To enhance the discriminative power of the analysis, this study used a modified version of the IPA proposed by Abalo et al [48]. This adaptation addresses the common issue of attribute saturation, whereby attributes tend to receive uniformly high ratings, limiting the ability to differentiate among them. The modified approach uses ordinal rankings instead of mean scores, improving the identification of priority areas, particularly relevant in health care settings [49] and training needs assessments [50].

Based on this approach, an IPA chart was generated using discrepancy scores from 3 key stakeholder groups: CP, NCP, and PC. CP were defined as members of health care organizations providing direct cancer care (eg, oncologists, radiotherapists, oncology nurses, family physicians, and community nurses). NCP included individuals performing administrative or managerial tasks related to cancer care, regardless of professional category. Caregivers were defined as those who provide physical, emotional, practical, and, in some cases, medical support to individuals diagnosed with cancer. These may be family members, close friends, or other persons designated by the patient who play a critical role across all stages of treatment and recovery. A patient with cancer was defined as any individual at any stage of the disease, including those undergoing active treatment, in remission (with favorable progression and no ongoing treatment), considered cured but undergoing regular follow-up, cured without active medical surveillance, or experiencing a relapse.

**Figure 1:**
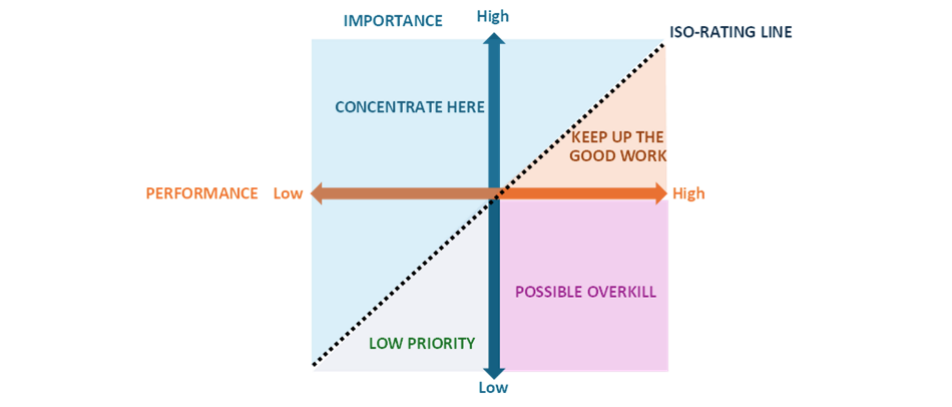
Representation of the alternative version of the importance-performance analysis (IPA) grid.

The position of each attribute was analyzed in relation to the diagonal and the corresponding quadrants of the IPA grid (see Figure 1).

The current literature highlights a significant gap in digital skills among health care professionals [51]. Furthermore, although it is widely recognized that digital skills in health care are inherently multifaceted, a standardized framework to delineate and prioritize the most essential skills remains absent. Furthermore, we expected not only training gaps but also differences between the health professionals included in the study.

Consequently, the aim of this study was to identify training gaps and prioritize the digital skill development needs in the oncology health care workforce through the application of IPA.

## Methods

### Study Design

A selective methodology was used. It involved conducting an online survey among a group of experts selected within the consortium of the European project. Given the exploratory nature of the study and the specific expertise required from participants, a convenience sampling approach was used to facilitate recruitment across diverse stakeholder groups and countries. This strategy was deemed appropriate due to the practical constraints of accessing individuals with relevant professional or lived experience in cancer care across several member states, all within a limited time frame.

The inclusion criteria required participants to be CP, NCP, or PC from member states of the TRANSiTION consortium, with the ability to comprehend and respond in English. Moreover, participants were required to have the capacity to provide informed consent. Recruitment was supported by consortium partners who identified and invited suitable individuals based on their direct involvement in cancer care or their experience as patients or caregivers. This pragmatic sampling approach enabled the collection of meaningful insights while ensuring feasibility in a multinational context.

### Instrument

An ad hoc online questionnaire, available exclusively in English, was developed by the research team. Although several validated instruments exist in the fields of DHL and eHealth for the general population [12,15], they did not adequately address the specific objectives or context of our study. The TRANSiTION project aimed to design a training course tailored to professionals involved in cancer care. Therefore, beyond assessing general skills, it was crucial to identify the specific learning needs, expectations, and contextual factors affecting this particular group. Given these requirements, the development of a customized instrument was deemed necessary to ensure the relevance, specificity, and practical utility of the collected data for informing the design of the training intervention.

The structure and items of the questionnaire were based on *DigComp 2.2: The Digital Competence Framework for Citizens* [32] and *Measuring What Matters: The Patient-Reported Indicator Surveys* [52]. For the development of the items, the contents of the *Core Curriculum in eHealth* from the EU×US initiatives were reviewed [53], as well as the document *Mapping Health Data Management Systems through Country Visits: Development, Needs, and Expectations of the EHDS* by TEHDAS [24].

The final selection of questionnaire items was reached by consensus in an online focus group conducted in May 2023. During this session, it was decided to include 2 additional and independent sections (ie, patient empowerment and ethics) informed by the prior work and practical experience of project partners who had previously identified core digital health skills as applied in professional practice [54].

The questionnaire was organized into 3 thematic sections. The first section included the information sheet and the consent form for participants. The second section addressed sociodemographic variables. The third section focused on assessing the need for training of 7 key digital skills: information, communication, content creation, safety, problem-solving, ethics, and patient empowerment. Moreover, 7-point Likert scale items were used to evaluate performance (with 1 representing “Very bad” and 7 representing “Very good”) and importance (with 1 representing “Not important” and 7 representing “Highly important”). The questionnaire items for CP and NCP were identical. Additionally, to make the results more representative, the third section asked about the performance of their colleagues within their organization from the respondent’s perspective, aiming to shift the focus away from self-assessment of personal skills. Similarly, the items on the importance of digital skills referred to how crucial these skills are for cancer care. In contrast, patients were asked about their perception of the digital skills shown by the cancer professionals attending to them and about the importance of these skills for their cancer care—or, in the case of caregivers, for the care of the patient. The information letters and questionnaires can be found in Multimedia Appendix 1.

### Procedure

The questionnaire was administered as a closed, invitation-only survey, accessible exclusively to individuals invited by TRANSiTION consortium partners. It was fully compatible with mobile phones, tablets, and computers across all major operating systems. To prevent multiple entries from the same individual, participants were authenticated through their email address prior to receiving single-use access to the survey platform.

Recruitment was facilitated by the TRANSiTION consortium, which disseminated information about the study to eligible participants. The consortium consisted of 24 European reference partners in the field of cancer care, including research institutes, universities, hospitals, oncology centers, and patient organizations [38].

The survey was piloted by 8 members of the Spanish partners of the TRANSiTION consortium during June 2023 and July 2023. Based on the pilot, minor adjustments were made to improve the wording of certain items. On average, it was estimated that participants required approximately 15 minutes to complete the online questionnaire. Additionally, it was suggested to include the item “Have you received prior training in digital competencies/skills?” with a dichotomous response option of “Yes” or “No.”

Participants were recruited during July 2023 and August 2023. Data were collected and stored using an online questionnaire implemented through the eDelphi website [55] in September 2023. To ensure procedural standardization, daily monitoring of the data collection process was conducted, allowing for the immediate resolution of any questions or technical issues that arose.

### Data Analysis

Data analysis included descriptive statistics, reported as mean and SD. Internal consistency of the items assessing digital skill domains was evaluated using Cronbach α. Normality was assessed using the Shapiro-Wilk test. In cases where the assumption of normality was not met, nonparametric tests were applied. Specifically, group comparisons of importance and performance scores across CP, NCP, and PC were conducted using 1-way ANOVA or, when appropriate, the Kruskal-Wallis test. All analyses were performed using SPSS version 25.0 (IBM Corp).

### Ethical Considerations

The study was reviewed and approved by the Pontevedra-Vigo-Ourense Research Ethics Committee (reference: 2023/309).

Before the survey, they were informed about the study’s objectives, reminded of the voluntary nature of their participation, and asked to provide informed consent.

The online questionnaire platform provides a confidential, single-use access system to the questionnaire for each participant, ensuring the confidentiality and anonymity of responses. No financial compensation was provided to the participants.

## Results

### Demographics

Initially, 152 participants expressed interest in taking part in the study. Of these, 33 were excluded for not meeting the inclusion criteria. Specifically, 24 participants who had registered as CP or NCP were in fact students without professional experience, and 9 PC were excluded due to insufficient English proficiency. Additionally, 28 participants (12 CP, 10 NCP, 3 patients, and 3 caregivers) accepted the informed consent but did not begin the questionnaire. A further 24 participants (8 CP, 10 NCP, 3 patients, and 3 caregivers) declined to provide informed consent.

A total of 67 participants completed the study: 38 CP, 16 NCP, and 13 PC. All participants who completed the survey responded to all questionnaire items. Of the total, 50 participants were women (50/67, 75%), participants were primarily aged between 31 years and 45 years (27/67, 40%), and most resided in municipalities with populations greater than 100,000 (44/67, 66%). Table 1 presents the main sociodemographic characteristics of the sample, stratified by group.

**Table 1:** Sociodemographic data of the participants, by group.

Characteristic			CP^a^ (n=38)		NCP^b^ (n=16)		PC^c^ (n=13)
Gender (female), n (%)			29 (76)		12 (75)		9 (69)
**Age (years), n (%)**							
	18-30	10 (26)		5 (31)		0 (0)	
	31-45	14 (37)		7 (44)		6 (46)	
	46-60	11 (29)		3 (19)		5 (39)	
	≥61	3 (8)		1 (6)		2 (15)	
**Population of the resident municipality, n (%)**							
	<50,000	7 (18)		2 (13)		5 (39)	
	50,000-100,000	6 (16)		1 (6)		2 (15)	
	>100,000	25 (66)		13 (81)		6 (46)	

^a^CP: clinical professionals*.*

^b^NCP: nonclinical professionals.

^c^PC: patients and caregivers.

The participants were from 11 European countries: Belgium (3 CP, 5 NCP, and 2 PC), Bulgaria (10 CP and 2 NCP), Croatia (4 CP, 1 NCP, and 3 PC), Cyprus (6 CP), Greece (1 CP, 1 NCP, and 1 PC), Italy (1 CP, 1 NCP, and 2 PC), Lithuania (1 CP and 1 NCP), Poland (1 PC), Portugal (3 CP, 1 NCP, and 2 PC), Slovenia (1 CP, 1 NCP, and 1 PC), and Spain (8 CP, 3 NCP, and 1 PC).

The professions of the health care professionals were diverse. Among CP (n=38), 16 (42%) were oncologists, 12 (32%) were oncology nurses, 6 (16%) were clinical researchers, and 4 (11%) worked in other clinical professions related to cancer care. Additionally, 19 (50%) worked in public organizations, 12 (32%) worked in subsidized private organizations, 4 (11%) worked in nonsubsidized private organizations, and 3 (8%) preferred not to specify. Among NCP (n=16), 5 (31%) were clinical data managers, 4 (25%) were part of the administrative staff related to cancer care, 3 (19%) worked in health care service management, and 4 (25%) were in other nonclinical professions related to cancer care. Furthermore, 8 (50%) worked in public organizations, 7 (44%) worked in subsidized private organizations, and 1 (6%) preferred not to specify. All PC (n=13) were users of public health care services.

### Internal Consistency

As shown in Table 2, the internal consistency of the digital skills questionnaire was high across all domains (items B1 through B7 in Multimedia Appendix 1) and participant groups. At the global level, Cronbach α values ranged from 0.92 (information) to 0.97 (ethics), indicating excellent reliability. When analyzed by subgroup, responses from CP and PC consistently showed very high internal consistency, with α values greater than 0.85 in all domains. Responses from NCP also showed acceptable to excellent consistency, though slightly lower in the domains of eHealth problem-solving (α=0.86) and ethics (α=0.90). These results support the internal reliability of the instrument across different respondent profiles.

**Table 2:** Internal consistency of the digital skill domains.

Digital skills	Global, Cronbach α	Clinical professionals, Cronbach α	Nonclinical professionals, Cronbach α	Patients and caregivers, Cronbach α
Information	0.92	0.95	0.91	0.85
Communication	0.93	0.95	0.89	0.90
Content creation	0.95	0.95	0.92	0.95
Safety	0.96	0.96	0.95	0.97
eHealth problem-solving	0.94	0.97	0.86	0.92
Ethics	0.97	0.97	0.90	0.97
Patient empowerment	0.96	0.96	0.90	0.98

### Performance and Importance Scores

This section presents the results from the third part of the questionnaire, which focused on 7 core digital competencies relevant to cancer care professionals. Additional findings related to perceived training needs are available in Multimedia Appendix 2. The 7 competencies assessed were information, communication, content creation, safety, eHealth problem-solving, ethics, and patient empowerment.

Information is defined as the ability to search, evaluate, and manage digital health information effectively. Communication refers to the capacity to interact, share, and collaborate using digital tools in health care contexts. Content creation is understood as the skill to produce, edit, and adapt digital content appropriately for clinical use. Safety encompasses data protection, privacy, and cybersecurity practices. Problem-solving is the ability to identify and resolve technical or digital challenges in the care process. Ethics is related to the understanding and application of ethical principles such as confidentiality, consent, and digital equity. Patient empowerment is defined as the ability to support patients with using digital tools to actively participate in their care.

Table 3 shows the importance and performance results for CP, NCP, and PC. As can be seen, all performance and importance scores reached notably high values, reflecting that oncology health care professionals perceive themselves as having strong digital skills, and, at the same time, that digital literacy is considered important in cancer care. Moreover, 22 of 38 CP (58%) reported having received prior training in digital skills, a figure that reached 11 of 16 NCP (69%).

**Table 3:** Importance and performance for clinical professionals (CP; n=38), nonclinical professionals (NCP; n=16), and patients and caregivers (PC; n=13).

Digital skills by group		Performance, mean (SD)^a^	Importance, mean (SD)^b^
**Information**			
	CP	6.18 (0.98)	6.34 (1.34)
	NCP	5.88 (0.96)	5.94 (1.23)
	PC	5.85 (2.07)	5.23 (1.59)
**Communication**			
	CP	6.03 (0.97)	6.45 (1.29)
	NCP	5.94 (0.85)	5.69 (1.08)
	PC	5.23 (1.78)	5.31 (1.32)
**Content creation**			
	CP	5.37 (1.58)	5.79 (1.36)
	NCP	5.56 (1.03)	5.44 (1.03)
	PC	4.15 (2.03)	4.15 (2.03)
**Safety**			
	CP	5.21 (1.49)	6.08 (1.56)
	NCP	5.25 (1.06)	5.81 (1.05)
	PC	4.69 (1.84)	4.62 (2.10)
**eHealth problem-solving**			
	CP	5.53 (1.31)	6.18 (1.37)
	NCP	5.56 (1.09)	5.81 (1.17)
	PC	4.38 (1.80)	4.38 (1.55)
**Ethics**			
	CP	5.61 (1.28)	6.03 (1.50)
	NCP	5.50 (1.15)	5.75 (1.06)
	PC	4.92 (2.10)	4.38 (1.85)
**Patient empowerment**			
	CP	5.50 (1.45)	6.32 (1.36)
	NCP	5.56 (1.09)	6.00 (1.15)
	PC	5.08 (2.11)	5.23 (1.36)

^a^Overall mean (SD): 5.38 (1.47).

^b^Overall mean (SD): 5.57 (1.40).

The digital skills that showed the highest performance among CP were information (mean 6.18, SD 0.98) and communication (mean 6.03, SD 0.97). Similarly, these same digital skills, but in reverse order, were considered the most important for cancer care (mean 6.45, SD 1.29; mean 6.34, SD 1.34, respectively). Additionally, patient empowerment digital skills achieved a similar level of importance (mean 6.32, SD 1.36). In contrast, the digital skill with the lowest performance and importance scores was content creation (mean 5.37, SD 1.58).

Digital communication (mean 5.94, SD 0.85) and information (mean 5.88, SD 0.96) skills exhibited the highest performance scores among NCP. In contrast, the skills considered most important for cancer care were those related to patient empowerment (mean 6.00, SD 1.15). In comparison, the digital skills of content creation and eHealth problem-solving demonstrated the lowest performance scores (mean 5.56, SD 1.03; mean 5.56, SD 1.09, respectively), with the former also showing the lowest importance score (mean 5.44, SD 1.03).

For PC, the digital skills in which they thought oncology health care professionals perform best were communication (mean 5.23) and information (mean 5.85). The digital skills considered most important were communication (mean 5.31, SD 1.32) and patient empowerment (mean 5.23, SD 1.36). In contrast, the lowest performance and importance scores were observed for the digital skills related to content creation (both: mean 4.15, SD 2.03).

### Discrepancy Analysis

Table 4 presents the results of the discrepancy analysis, defined as the mean difference between performance and importance for each of the digital skills. As shown, for CP, information digital skills exhibited the most positive discrepancy (mean difference 0.62). Conversely, safety (mean difference –0.87) and patient empowerment (mean difference –0.82) digital skills showed the most negative results. Regarding NCP, communication digital skills displayed the most positive discrepancy (mean difference 0.25), while safety digital skills had the most negative discrepancy (mean difference –0.56). For PC, the highest positive discrepancy was observed for information digital skills (mean difference 0.62), whereas the most negative discrepancy pertained to patient empowerment digital skills (mean difference –0.15).

**Table 4:** Discrepancy for clinical professionals (CP; n=38), nonclinical professionals (NCP; n=16), and patients and caregivers (PC; n=13).

Digital skills, by group			Discrepancy, mean difference		*F* (*df*)			*H*	
**Information**						1.13 (2)			3.58
	CP	–0.16							
	NCP	–0.06							
	PC	0.62							
**Communication**						1.25 (2)			6.50*
	CP	–0.42							
	NCP	0.25							
	PC	–0.08							
**Content creation**						0.65 (2)			1.26
	CP	–0.42							
	NCP	0.12							
	PC	0							
**Safety**						1.10 (2)			2.89
	CP	–0.87							
	NCP	–0.56							
	PC	0.07							
**eHealth problem-solving**						0.85 (2)			1.86
	CP	–0.65							
	NCP	–0.25							
	PC	0							
**Ethics**						1.24 (2)			2.19
	CP	–0.42							
	NCP	–0.25							
	PC	0.54							
**Patient empowerment**						0.70 (2)			2.76
	CP	–0.82							
	NCP	–0.44							
	PC	–0.15							

Notably, despite the small sample size, statistically significant differences were identified in the discrepancies associated with communication digital skills. Specifically, the discrepancy was positive for CP, negative for NCP, and nearly neutral for PC (–0.42 vs 0.25 vs –0.08; *H*= 6.50, *P*=.04.

### IPA Chart and Key Findings

**Figure 2:**
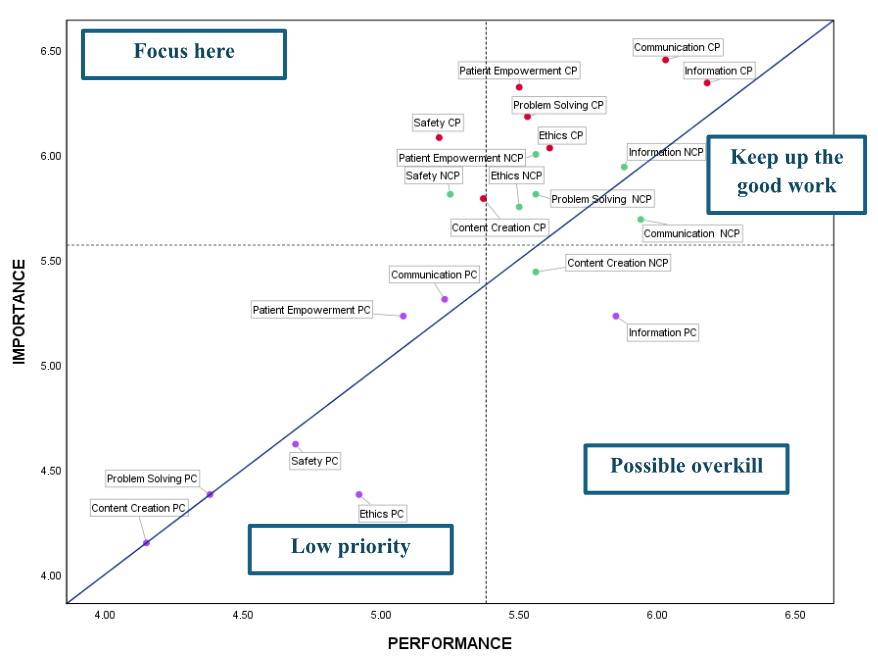
Importance-performance analysis (IPA) graph comparing clinical professionals (CP), nonclinical professionals (NCP), and patients and caregivers (PC).

As a result of these discrepancies, the IPA chart was developed (Figure 2). The axes of the graph were formed using the mean scores of performance (mean 5.38) and importance (mean 5.57), as shown in Table 2. Results were segmented for CP (in red), NCP (in green), and PC (in purple). This segmentation allowed for the identification of high-priority training needs, as well as areas where training is of low priority or may even represent a misallocation of resources. Two key findings emerged from the overall analysis.

First, both CP and NCP perceived a greater need for training compared with the skills PC attributed to them. This is evident, as most elements related to health care professionals are positioned above the diagonal, with some at a significant distance. In contrast, digital skills for PC are located on or near the diagonal, with a few even below it.

Second, all digital skills for CP fell within the “Focus here” quadrant, indicating that, although their prioritization may vary, all these skills require targeted training for this group. When evaluating each element individually, the IPA chart confirmed that patient empowerment and, particularly, security skills are the furthest from the diagonal and within the “Focus here” quadrant for both CP and NCP. Consequently, these represent the most critical digital skills requiring training.

For NCP, security and patient empowerment skills were similarly the top training priorities. Notably, communication skills met the expected importance levels (located in the “Keep up the good work” quadrant), whereas content creation skills were deemed redundant (falling into the “Possible overkill” quadrant).

In the case of PC, ethical skills fell into the “Low priority” quadrant, while information skills are in the “Possible overkill” quadrant. The remaining digital skills clustered near the diagonal, indicating a moderate level of alignment between their perceived importance and performance.

## Discussion

### Principal Findings

This study identified key training gaps and digital skill development priorities within the oncology health care workforce by applying IPA to a diverse panel of experts. The panel included 3 stakeholder profiles: CP, such as physicians and nurses; NCP, including managers, educators, and researchers; and PC.

A major finding was the assessment of 7 digital skills (information, communication, content creation, safety, eHealth problem-solving, ethical, and patient empowerment) and the specific training needs associated with each stakeholder group.

Notably, the prioritization of these competences differed across groups. For CP, the analysis revealed that all digital skills require further training, with safety and patient empowerment emerging as the highest priorities. NCP showed a comparable pattern, although they assigned less importance to content creation. None of the competencies was considered a low priority in this group.

In contrast, PC viewed information digital skills as having high performance but relatively low importance. The remaining competencies were positioned in the low performance–low importance quadrant, though communication and patient empowerment were perceived as the most important among them.

These findings underscore the need for tailored digital training strategies that reflect the differing perceptions and priorities of each stakeholder group.

### Comparison With Other Studies

Several previous studies have highlighted the widespread lack of digital literacy among health care professionals [56], which has led to the development of initiatives aimed at addressing this issue, particularly in the oncology field [38,57]. As Foadi and Varghese [58] suggested, although CP demonstrate a high level of proficiency with using specialized software for their daily tasks, they do not receive sufficient training on the basic principles of digital systems, which are essential for thriving in an ever-evolving digital health care environment [59]. In fact, Ramachandran et al [60] emphasized that, although health care professionals should demonstrate a general orientation toward digital skills, opportunities are created for specialized digital skills, particularly for the safe and equitable use of new technologies. Regarding cancer care, the study by Barbosa et al [61] identified digital safety skills as one of the 6 key domains for therapeutic radiographers and radiation therapists. Concerning patient empowerment, Navarro Martínez et al [62] emphasized a worrisome trend: Nurses, especially younger ones, exhibit limited or negligible use of technology to empower patients.

Incorporating the experience of health care service users is a central principle for the transition to digital health care with a patient-centered approach [63], making it essential to understand what PC expect regarding the digital skills of their oncology professionals. eHealth policies should be designed to consider the diverse perspectives of health professionals, patients, and caregivers. Furthermore, it is essential to bridge the digital divide for patients with cancer who have low digital literacy, enabling them to effectively use digital platforms designed by professionals [64]. In this regard, it is noteworthy that this study highlights that NCP perceived training in digital content creation skills as a potential misallocation of resources. Therefore, although these digital skills are important for enhancing cancer care, they should not be prioritized in training programs for this professional profile. The results suggest that enhancing digital information skills training within the oncology workforce may not be a pressing priority, as indicated by feedback from PC. However, this does not imply that patients with cancer are uninterested in receiving information on all aspects of the disease [65] nor that they are unwilling to embrace the potential of telecommunications to access relevant information [66]. In fact, the most necessary digital health function among patients with cancer and caregivers is information and education on symptom management following cancer treatment [67], and the use of digital health technology can be experienced as a person guiding them during their cancer treatment [68]. However, as previous studies noted, the interest of patients with chronic diseases in receiving health information through digital modalities is often hindered by educational and age-related gaps. In many cases, this must be preceded by social and instrumental support from health promoters [69].

This study has also yielded a significant finding when comparing results across different groups: CP, NCP, and PC. Digital communication skills were rated as highly important by all 3 groups. This is consistent with the fact that social support through digital tools provides significant benefits for both patients and caregivers during cancer treatment [70]. However, when analyzing discrepancies between CP, NCP, and PC, we found that the discrepancies between performance and importance were statistically significant. The results indicate that digital communication skills training is a priority for CP, somewhat unnecessary for NCP, and considered almost neutral by PC. First, NCP have limited direct interactions with patients, as their primary responsibilities center on the management and administration of services, which may account for the observed differences between CP and NCP. Accordingly, communication skills training for cancer care professionals who interact directly with patients has become a critical focus, using structured checklists to systematically assess oncologists’ behaviors during specialized doctor-patient consultations [71]. Second, the review by Henry et al [72] emphasized that digital communication skills are essential for CP to perform telehealth tasks, which may explain the priorities for training in this group. Third, although patients with cancer consider online platforms a preferred option for cancer follow-up consultations and delivering good news, they are not seen as suitable for initial visits or discussing bad news [73]. Therefore, PC may demonstrate a less favorable stance toward digital communication skills than CP. For instance, when oncology nurses and surgeons do not mention electronic patient-reported outcome measures, patients also refrain from discussing them [74].

Finally, it is worth mentioning that, in comparison, both CP and NCP are more critical of their digital skills training needs than PC. Overall, health care professionals recognize that they are not achieving a level of performance that aligns with the importance of these skills for cancer care. This finding, in which service providers perceive a worse outcome than users, has been observed in other studies using IPA [47]. This apparent “halo effect,” where PC may rate professionals’ digital skills more positively than professionals rate themselves, could reflect a high level of trust in health care providers. This is particularly relevant in the context of implementing new technologies and addressing the urgent need for digital skills training, as it suggests that end users may be more receptive to digital innovation than expected, potentially easing the path for adoption and integration of new tools in cancer care.

### Strengths and Limitations

This study has several strengths. It sought input from a sample of experts purposely selected by the TRANSiTION project consortium [38]. The partners belong to 14 countries from different geographic regions, with different types of organizations and occupational profiles. The partners have the personal email addresses of their members. Therefore, this ensured variability in the professional profiles of the participants, as well as different geogeographical backgrounds; additionally, patient involvement in the long-term improvement of HL is an essential requirement [25], making the inclusion of their perspective in this gap analysis a notable strength. Furthermore, although the use of the IPA method is well-documented in the literature, to the best of our knowledge, it has not been applied to evaluate training needs in digital skills for health care professionals, specifically in cancer care. Therefore, this study represents an innovative approach that could serve as a methodological foundation for future research. In addition, the survey probes aspects of cancer care that are not included in more general questionnaires. Moreover, the internal reliability of the instrument across different respondent profiles supports the incorporation of these skills in training design or scale development.

This study also has several limitations. First, the questionnaire did not rely on previously validated scales to assess digital skills. However, there is currently no gold standard for measuring digital skills among health care professionals, and the instrument was developed based on established digital competence frameworks to ensure alignment with the study’s specific objectives. Moreover, the aim of the project was not to validate a scale but to collect meaningful data to inform the design of a massive open online course (MOOC) [38]. Although participants from several EU countries were included, the use of a nonprobabilistic, purposive sampling strategy through consortium partners limits the representativeness of the sample and the generalizability of the findings beyond the context of the TRANSiTION network. Additionally, as the survey was available only in English, 9 potential participants were excluded due to insufficient language proficiency, which may have introduced selection bias. The administration of the survey during the summer months also contributed to a relatively low response rate within a limited population. Moreover, the use of self-reported data introduces the possibility of response bias; although prior research supports the validity of self-report measures [75], the results should be interpreted with appropriate caution. Finally, although IPA is a widely used tool to guide priority setting in training and service improvement [43,44,46,76], it has certain methodological limitations [77]. The technique relies heavily on mean values to allocate items to quadrants, which may not fully capture the distributional characteristics of the data. In addition, the cut-off points, typically overall means, are somewhat arbitrary, which can affect the robustness and interpretability of the results. This study did not include a sensitivity analysis to explore the impact of alternative quadrant definitions, which could have helped mitigate this limitation. This decision was primarily influenced by the exploratory nature of the research and the limited sample size. Future research should address these aspects to improve the reliability of IPA-based prioritization.

### Impact on Organizations and Health

There is a growing body of literature that demonstrates the pivotal role of HL for achieving better health outcomes and higher quality of care [78]. Crucially, HL is modifiable, and improving HL is increasingly recognized as a way of improving outcomes, including in Europe’s Beating Cancer Plan. Therefore, the concept has rapidly gained an emerging strategic role in several governing bodies and cancer organizations, although more comprehensive implementation of interventions and strategies is still needed.

The information that cancer patients need to know for their diagnosis and treatment is indeed complicated. It includes a new language of health terminology, understanding consents for complex treatments and procedures, attending appointments at the right time and place, and seeking help appropriately and in a timely manner. Equally, citizens should have the necessary skills to interpret information and make appropriate decisions for cancer screening (eg, high-risk group of citizens) and prevention (eg, lifestyle behaviors). Competencies in HL and communication can significantly contribute to reducing barriers related to HL and to improving the quality of health care and health outcomes for patients [78]. However, studies have shown that health professionals tend to overestimate the HL of patients and citizens and lack adequate competence to compensate for it. Therefore, preparing staff to respond to patients’ HL is seen as a responsibility of health care organizations, which should be incorporated into their training programs.

Cancer care systems need to adapt to technological advancements by providing online health materials that are evidence-based, quality-controlled, reliable, and both culturally and linguistically appropriate. Therefore, the incorporation and management of digital technologies that facilitate interactions between health care professionals and patients seem essential in current and future training programs. It should be noted that the digital skills of CP involved in cancer care are multifaceted, and all of them are essential for providing high-quality cancer care [34]. Therefore, the findings of this study support the need to implement comprehensive training programs for CP that address the main digital skills cited in the literature [37].

It is worth mentioning that, although patient empowerment is a vague concept, it has been increasingly applied in cancer care over the past decade [33]. The accumulated evidence suggests that shared decision-making and the use of interactive digital tools lead to positive outcomes for cancer patients [79,80].

In addition, the future European Health Data Space, which aims to provide a coherent, reliable, and efficient system for the exchange and reuse of health data in research, innovation, policy-making, and regulation, will require a greater mastery of digital security skills for both CP and NCP [81].

The importance given by the European Union to digital skills training has already been outlined. Based on expert input, our consortium believes it is essential that countries incorporate DHL training at the earliest stages of education for health professionals and health managers and develop programs focusing on digital skills in oncology [29]—not only in the university setting but also in continuing education—including hands-on training through internships, clinical rotations, and simulation exercises and promoting interdisciplinary collaboration.

The extension and adaptation of competencies to the health environment represent an example to follow. The involvement so far of 60 countries worldwide and 1300 participants will facilitate this impact, supported by multiple meetings with European stakeholders [38] and diffusion through scientific societies and partners’ universities and national health services.

Finally, it points to research gaps for new scientific projects in the fields of social sciences and citizen science.

### Conclusions

This study conducted a gap analysis using the IPA to assess the digital skills of health care professionals in oncology and identified areas where further training is needed. The results highlight the necessity of developing comprehensive training programs for CP. Additionally, it underscores the critical importance of digital safety and patient empowerment skills for both PC and NCP. The study incorporates the perspectives of PC, who prioritize different training needs for health care professionals, placing comparatively less emphasis on digital information and ethical skills. These findings provide a knowledge base for designing training programs and eHealth policies, promoting a holistic approach that integrates the perspectives of the various stakeholders involved in digital cancer care.

### Policy Summary

Health care professionals acknowledge that their digital skills require enhancement across all areas. Specifically, doctors and nurses need additional training in digital problem-solving, communication, and, above all, skills linked to patient safety and empowerment. These priorities align with the perspectives of PC, who emphasize the critical need for health care providers to strengthen their digital communication and patient empowerment capabilities.

## Appendix

Multimedia Appendix 1 Clinical professional and nonclinical professional survey.

Multimedia Appendix 2 Additional analysis of training needs.

## Abbreviations

CP: clinical professionals
DHL: digital health literacy
HL: health literacy
IPA: importance-performance analysis
MOOC: massive open online course
NCP: nonclinical professionals
PC: patients and caregivers
TEHDAS: Towards European Health Data Space
