# Supplementary material for: Training Gaps in Digital Skills for the Cancer Health Care Workforce Based on Insights From Clinical Professionals, Nonclinical Professionals, and Patients and Caregivers: Qualitative Study
Source: JMIR Med Educ. 2025 Oct 8;11:e78490. doi: 10.2196/78490 (PMC12547342; doi:10.2196/78490)
Supplement: Multimedia Appendix 1 [file mededu_v11i1e78490_app1.docx]

Appendix I.

CP and NCP Survey

We would like to invite you to participate in a survey for the research activity carried out in the framework of the EU-funded TRANSiTION project (id: 101101261), which started in 2023 and will continue until 2025.

**WHAT IS THE PROJECT ABOUT?**

It aims to provide digital skills training for healthcare workers, focusing on the needs of clinical professionals, non-clinical professionals, patients and their informal carers for cancer prevention, diagnosis, treatment, and survivorship. The proposal aims to improve the digital competences of healthcare professionals and support their safe and effective use of existing digital tools, while increasing their readiness and willingness to adopt new ones. The training is expected to improve and re-skill healthcare staff in terms of use of digital solutions and digital readiness, but also to support a faster transition towards more efficient, cost-effective, and patient-centred healthcare models.

Clinical professionals and non-clinical professionals must increasingly combine their knowledge of clinical practice with their knowledge of technology and change management to ensure that digital technology fits the reality of care practice, ensure buy-in from frontline healthcare workers, and manage the cultural change needed to drive learning in healthcare organisations.

**PURPOSE OF THE SURVEY**

This survey is part of WP3 of the TRANSiTION project and aims to address the current training needs and knowledge of healthcare workers, non-healthcare workers, cancer patients and their caregivers. Your participation is particularly important to us, as it will provide us with essential information to guide the training programmes that will be developed in WP4.

**WHAT DO YOU NEED TO KNOW?**

We remind you that the answers shared are **CONFIDENTIAL** and will be anonymised in the elaboration of deliverables and scientific contributions. Your collaboration is **VOLUNTARY**. There are no right or wrong answers, better or worse. What we are interested in is the perspective and opinion of all those involved. All information provided will only be used as described in the project purpose.

As this is an international project, you should be aware that by agreeing to participate in the study, you are also giving your consent that the information you provide may be transferred to another country as part of the research collaboration and its publication. Of course, the project coordinator will ensure the security of your personal data in compliance with current EU legislation in terms of data protection.

**APPROVAL**

The project has been reviewed by the Pontevedra-Vigo-Ourense Research Ethics Committee (ref: 2023/309). In accordance with the General Data Protection Regulation, the WP3 Coordinator, Servizo Galego de Saúde (SERGAS), is responsible for ensuring that the processing of your personal health data has a lawful basis.

**WITHDRAW PARTICIPATION**

You can withdraw your consent at any time and without any reason. If you have any questions about the project, you can contact the WP3 coordinators.

**BLOCK I: CONSENT TO PARTICIPATE**

If you wish to participate, you must sign the declaration of consent below.

I declare that I am aware of the terms of this informed consent, the aims of the research, the forms of participation, the costs and risks involved, as well as access to information and the safeguarding of information produced in the study. I acknowledge that the information I provide during this research is strictly confidential and anonymous. Furthermore, it will be used for scientific dissemination purposes only. I have been informed that I may ask questions about the project at any time and that I may withdraw from the project at any time, without having to give explanations or suffer any consequences for such a decision.

**I agree to participate in this survey.**

**□ YES □ NO**

**BLOCK II:** **SOCIODEMOGRAPHIC VARIABLES**

To begin with, we would like you to answer the following questions.

**A1. Gender:**

| Male | Female | Another |
| --- | --- | --- |
| 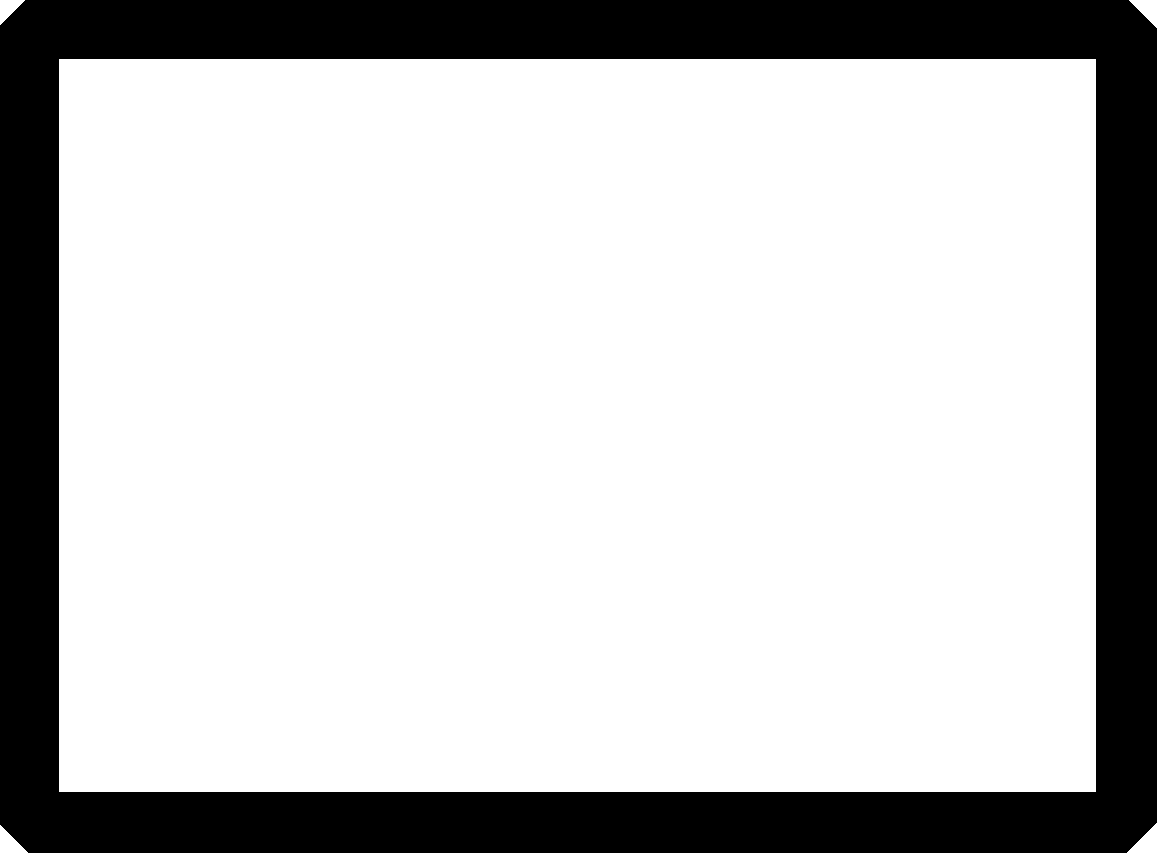 | 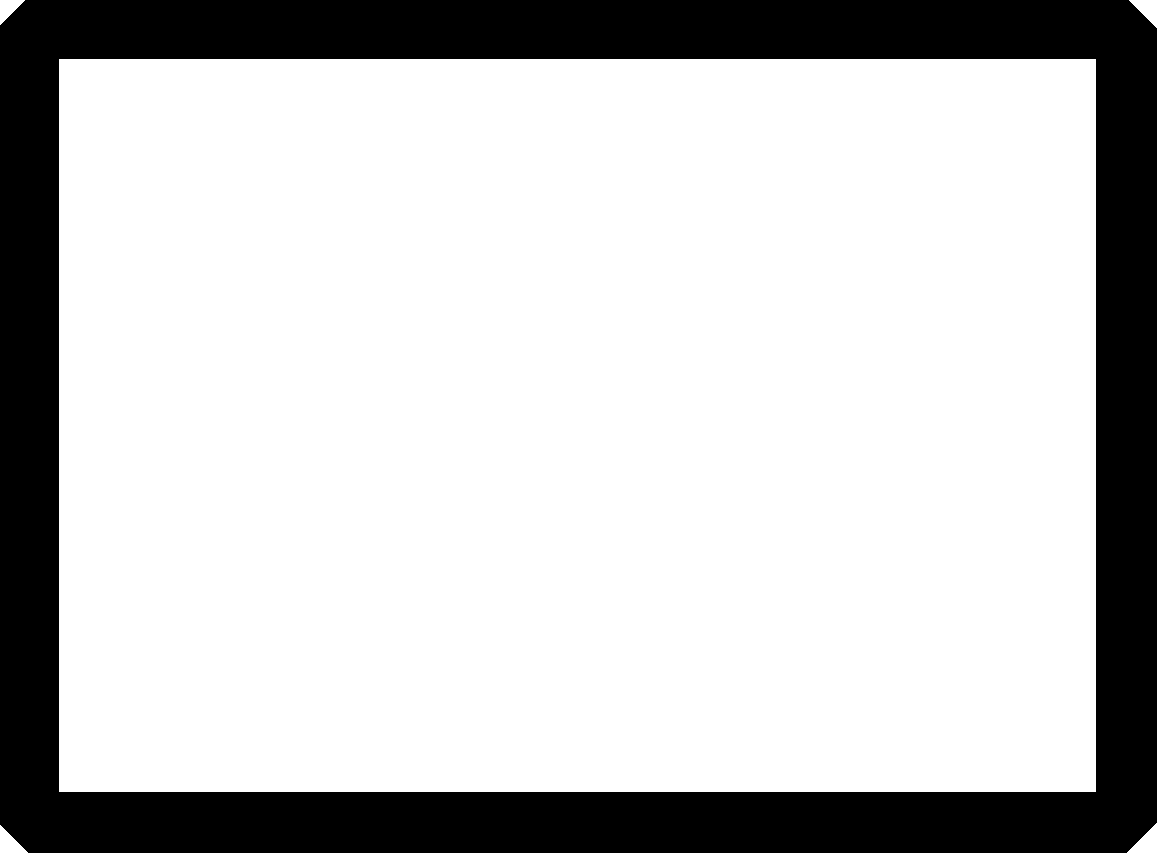 | 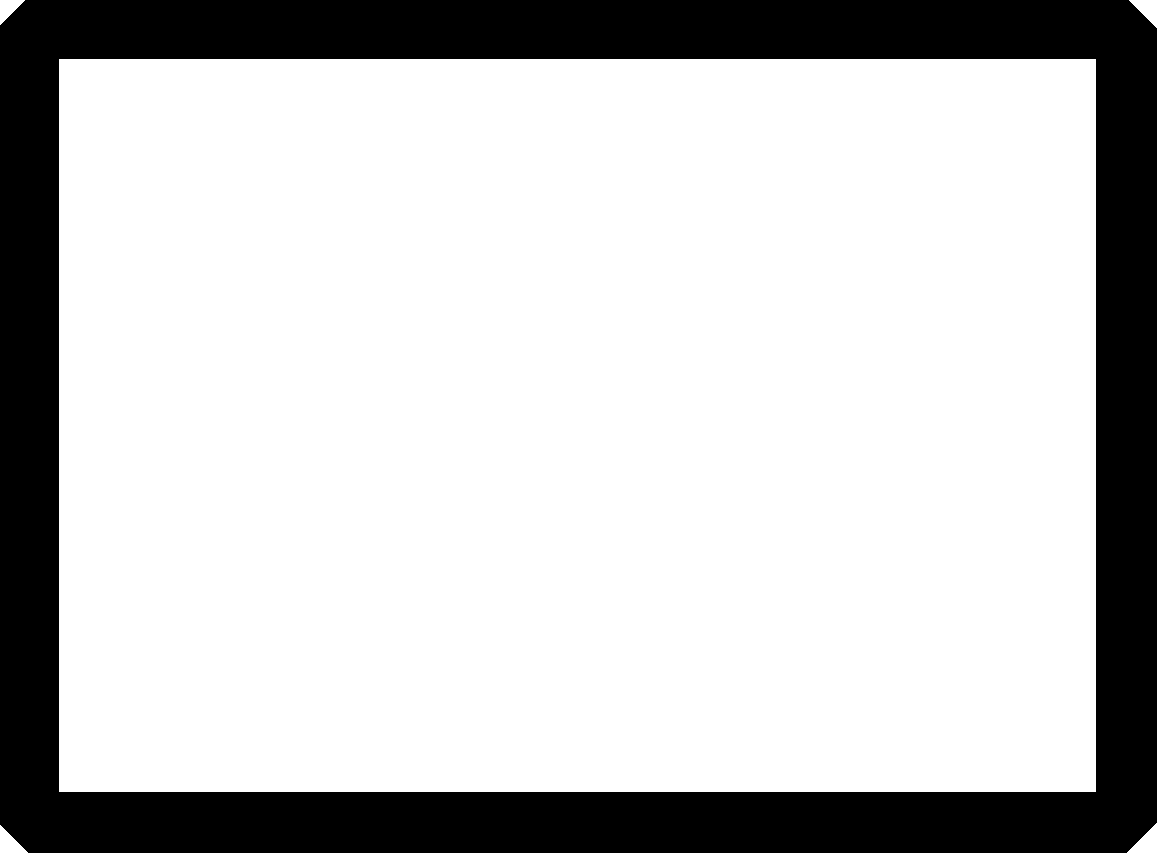 |

**A2. Age group:**

| 18-30 years | 31-45 years | 46-60 years | 61 years or more |
| --- | --- | --- | --- |
| 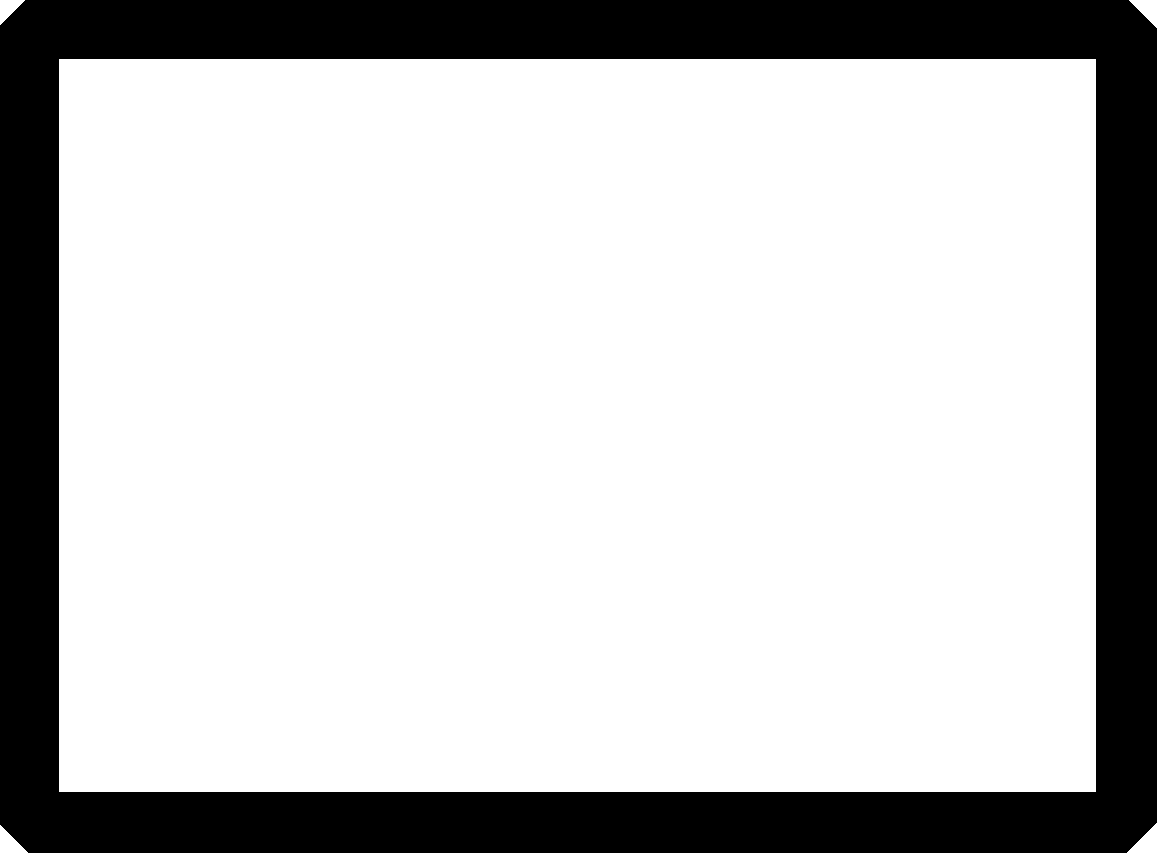 | 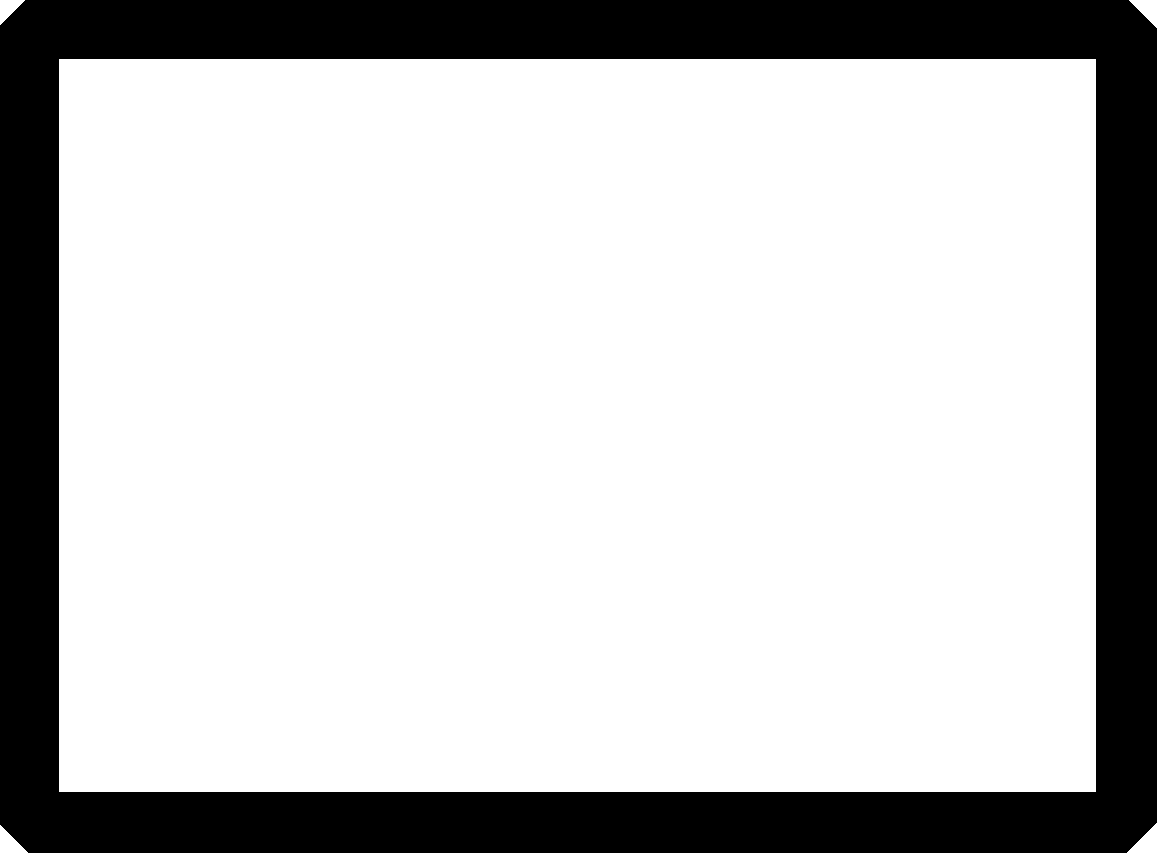 | 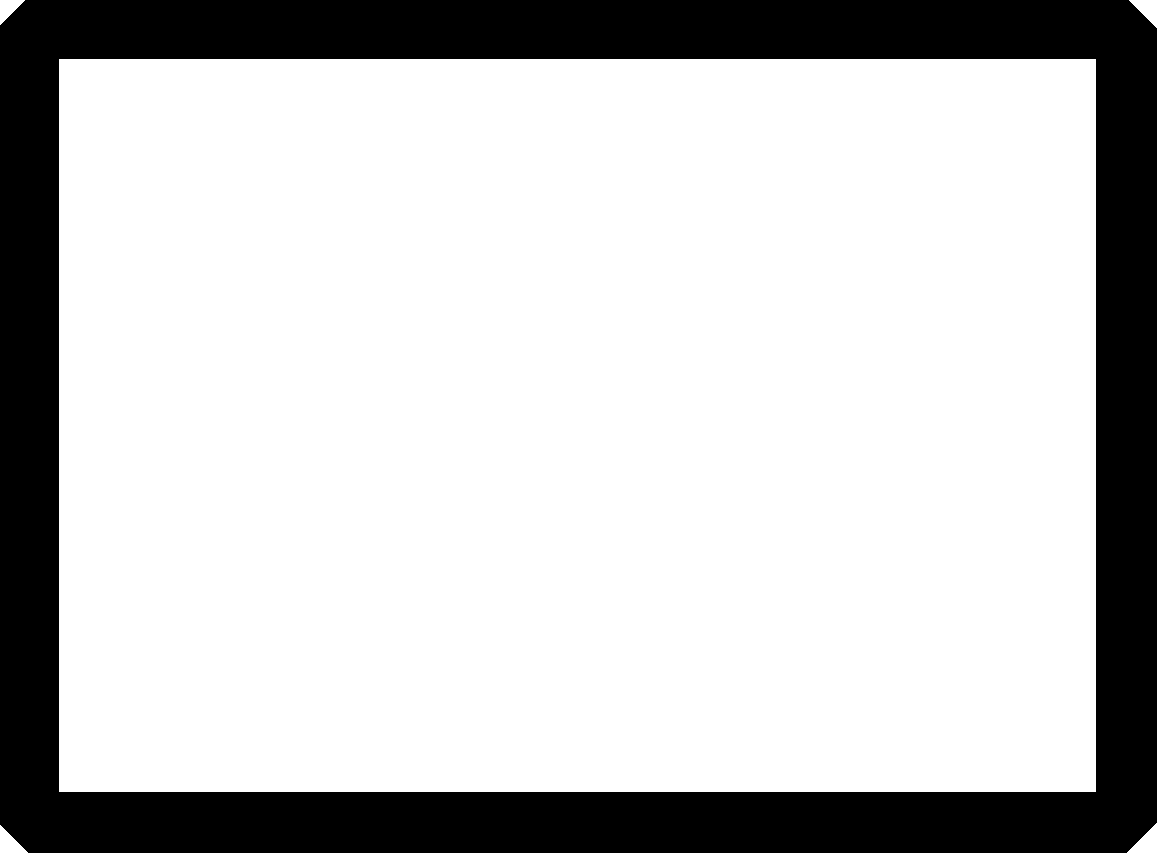 | 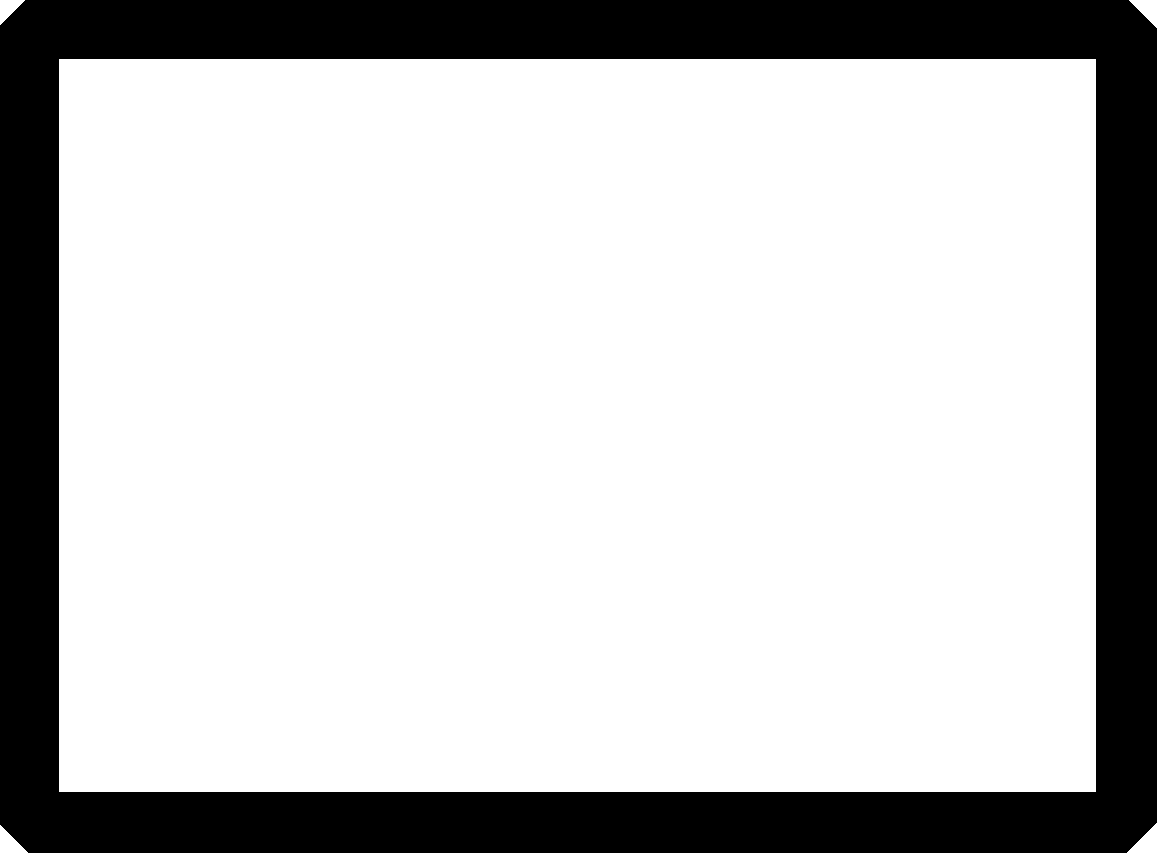 |
| **A3. Country of residence: ____________________________________________** |  |  |  |

| **A4.** How large is the population of your municipality/city where you usually live? |
| --- |
| 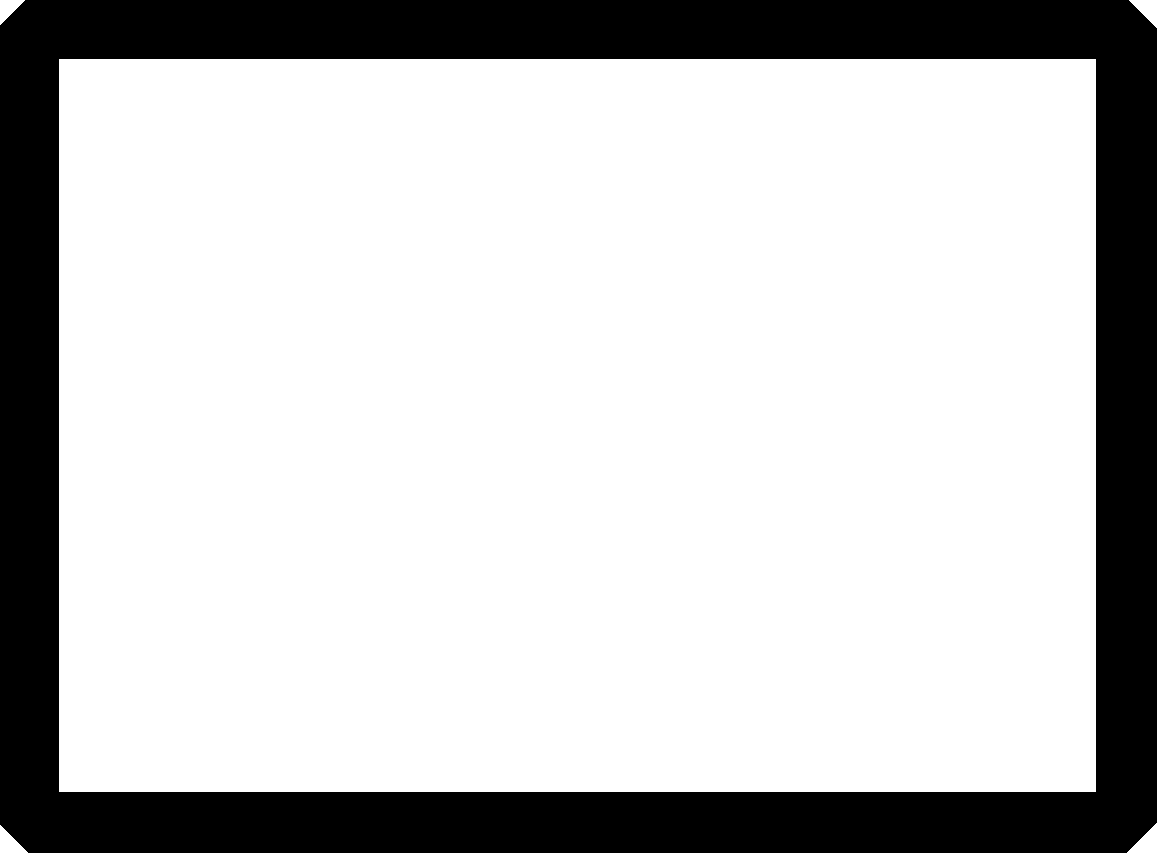 <50,000 population |
| 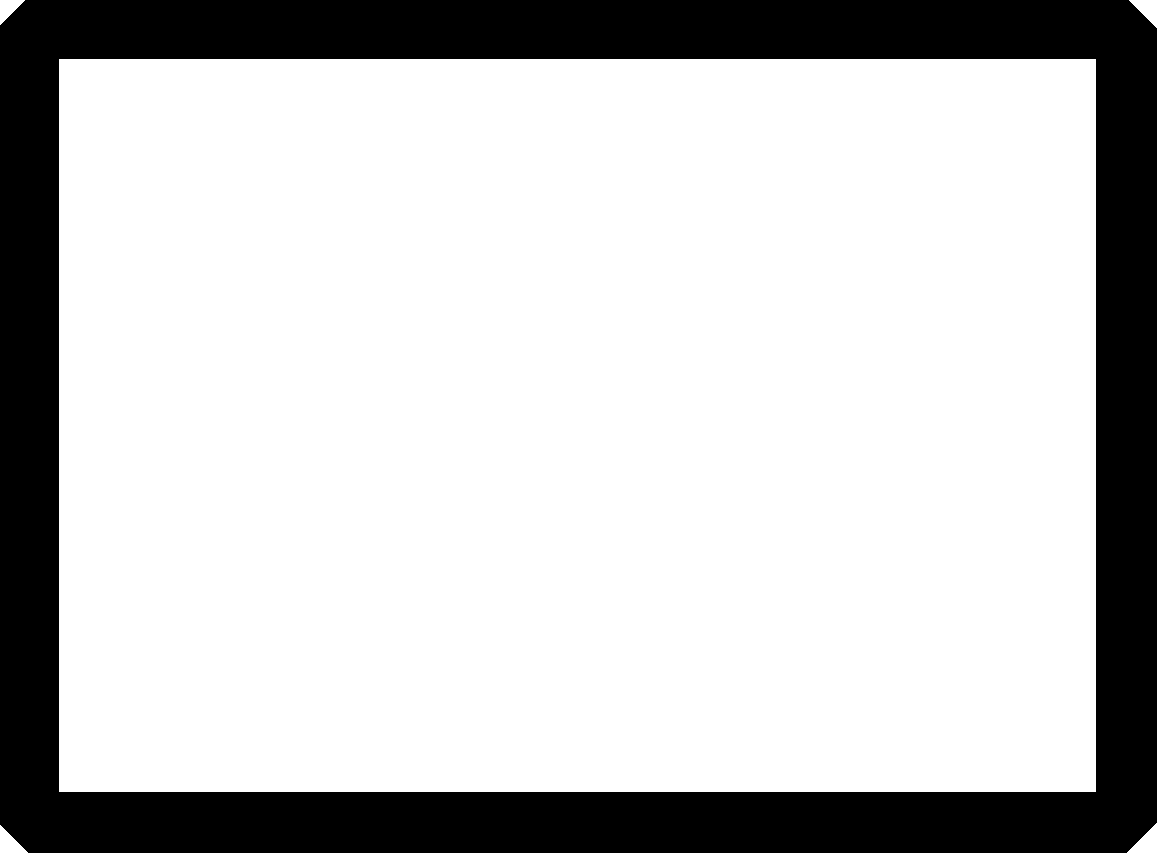 50,001-100,000 population |
| 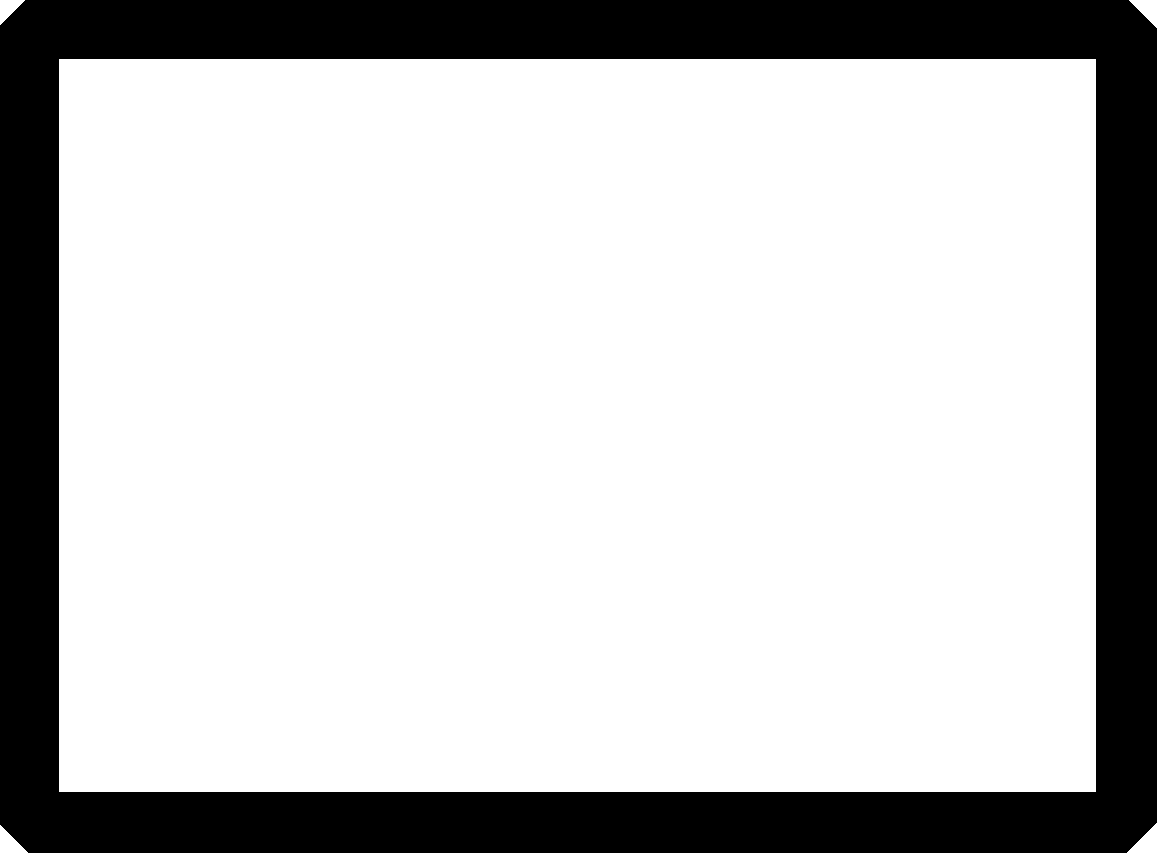 >50,000 population |

**A5. Group:**

| Clinical Professional | Non-clinical Professional |
| --- | --- |
| 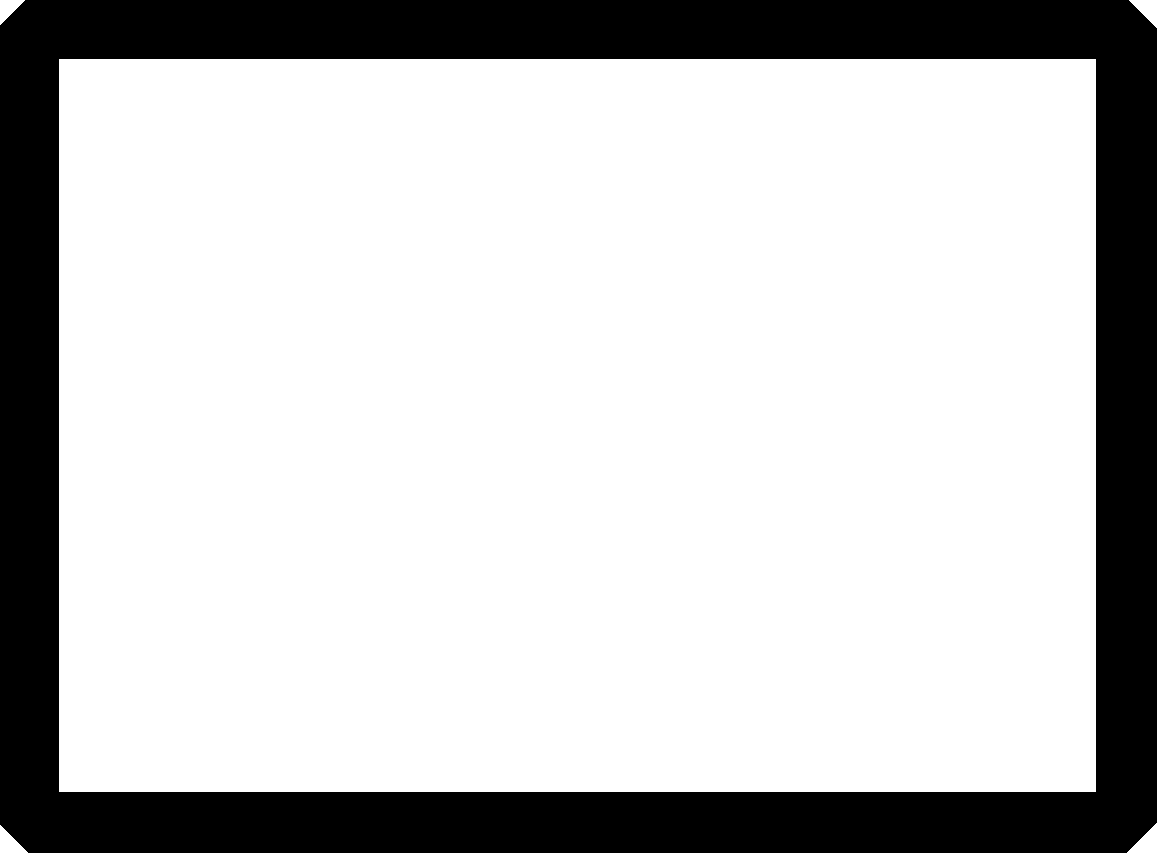 | 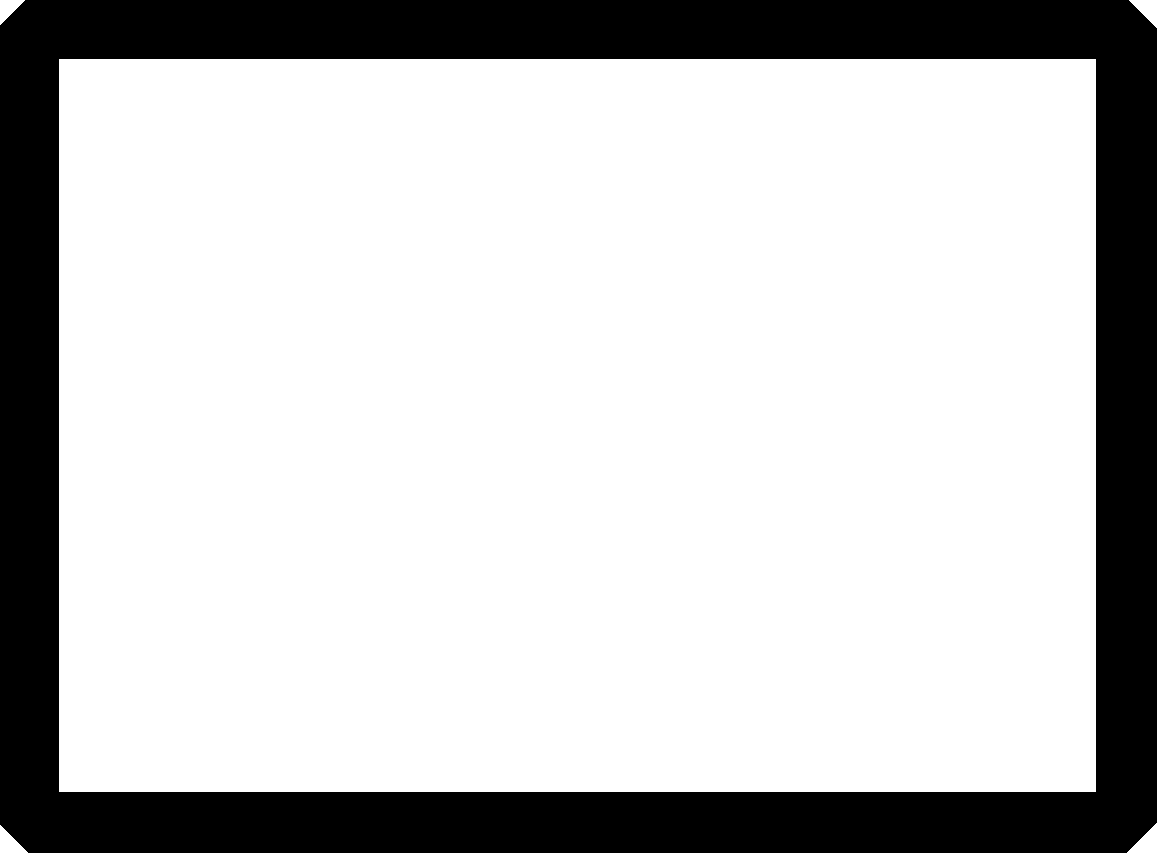 |

| **A6.** In what type of organisation do you work? |
| --- |
| 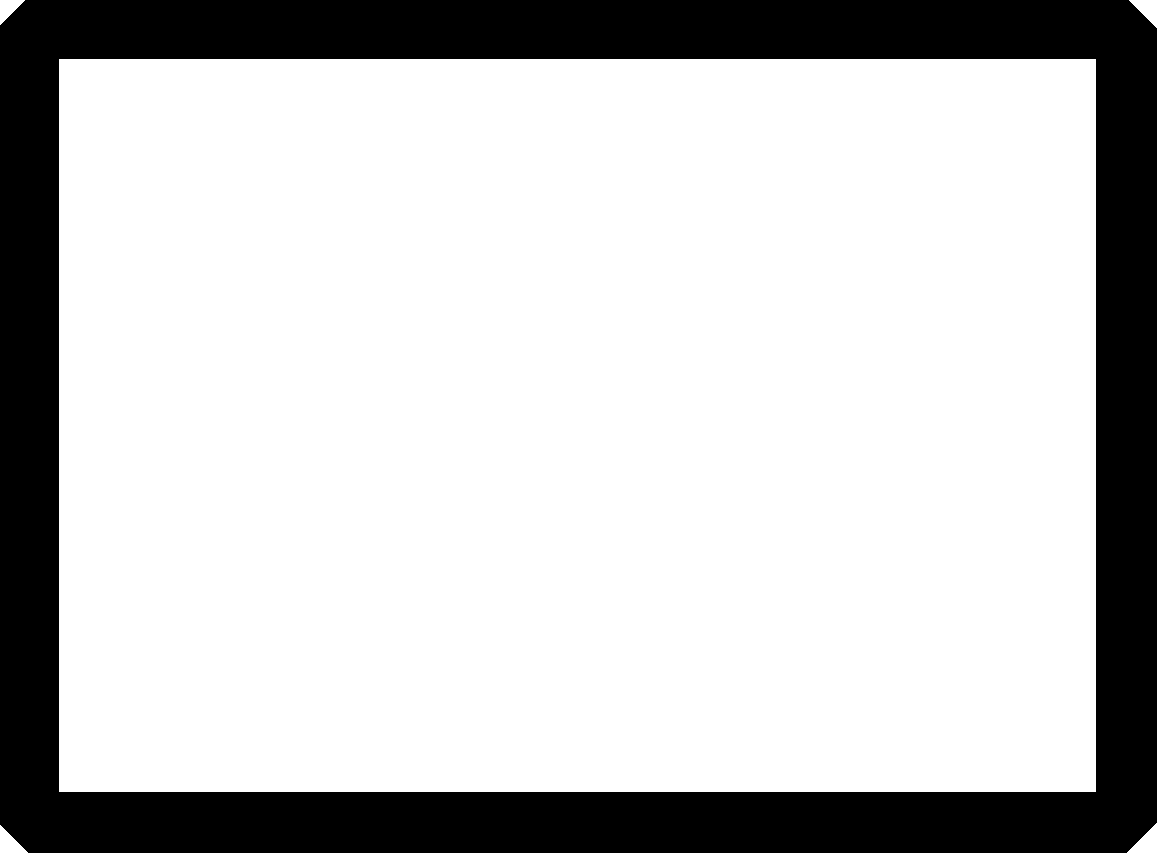 Hospital |
| 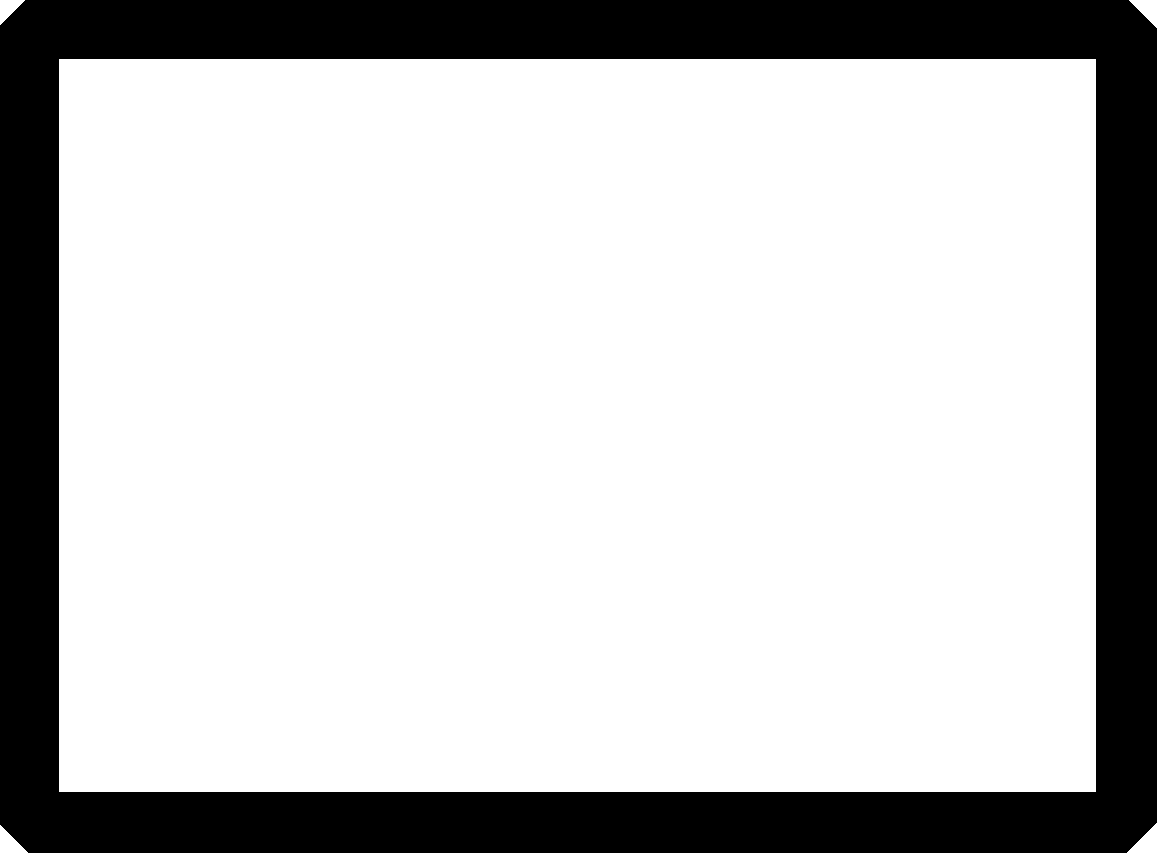 Primary care (health centres, outpatient clinics...) |
| 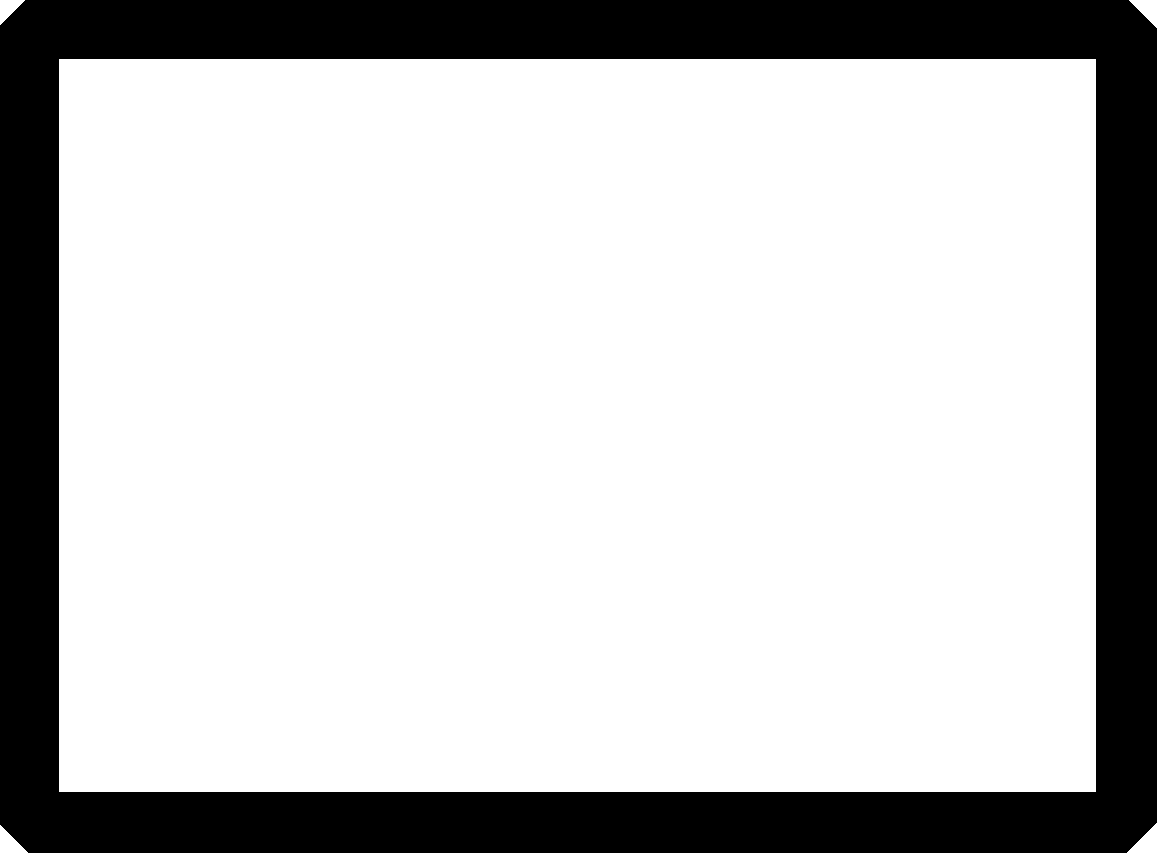 Research Centre |
| 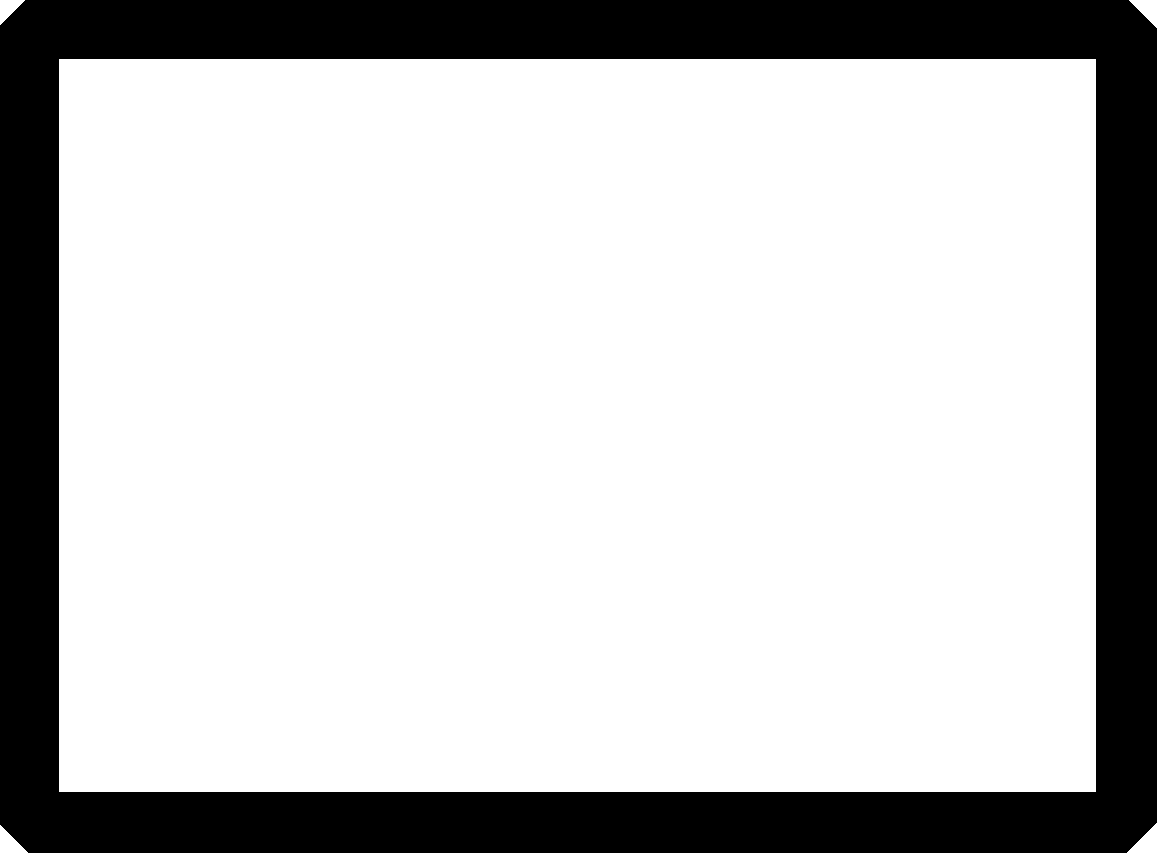 University |
| 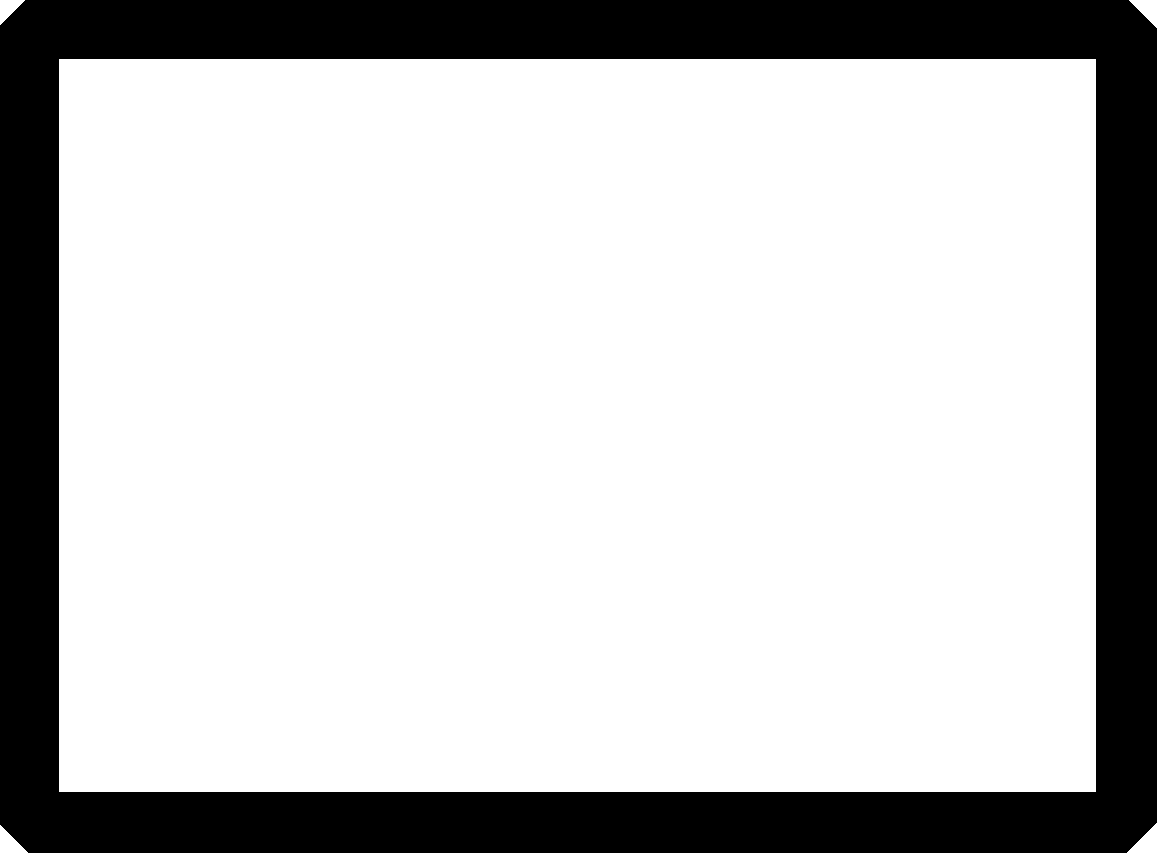 Other |

| **A7.** What is its ownership? |
| --- |
| 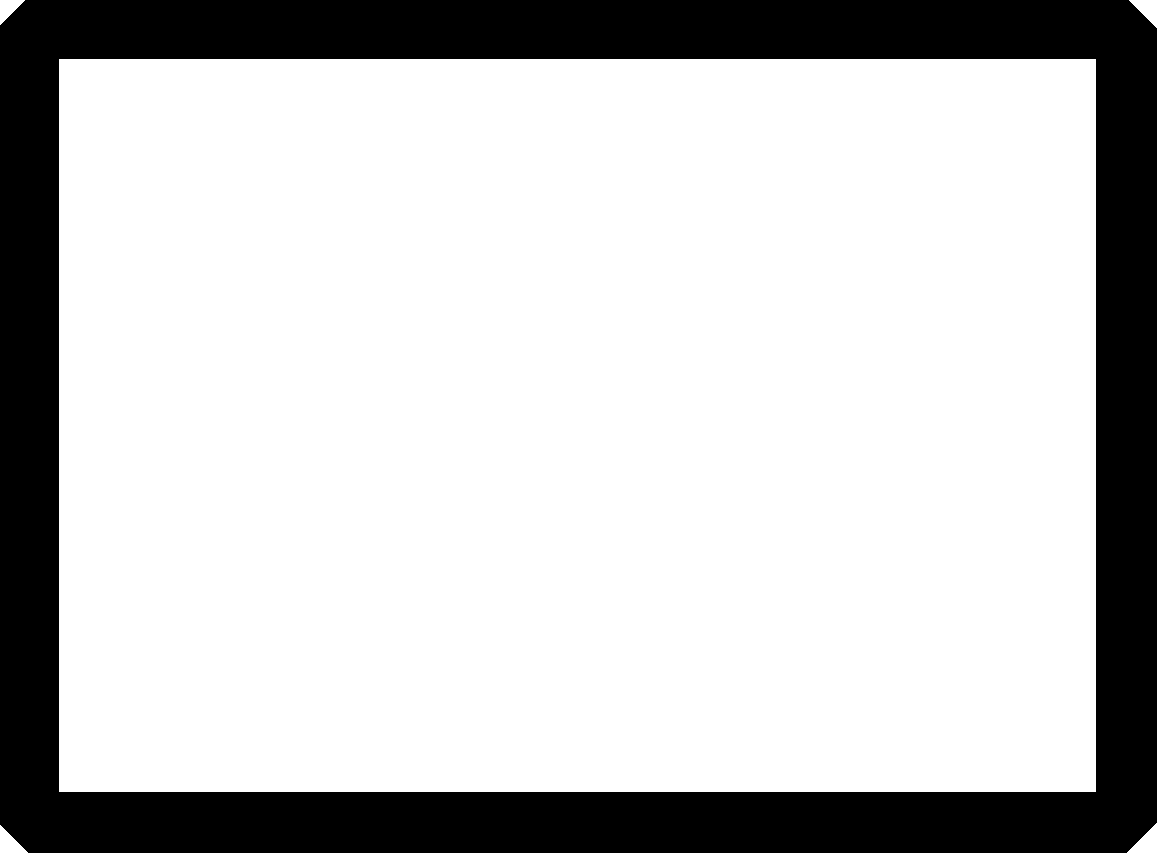 Public owned |
| 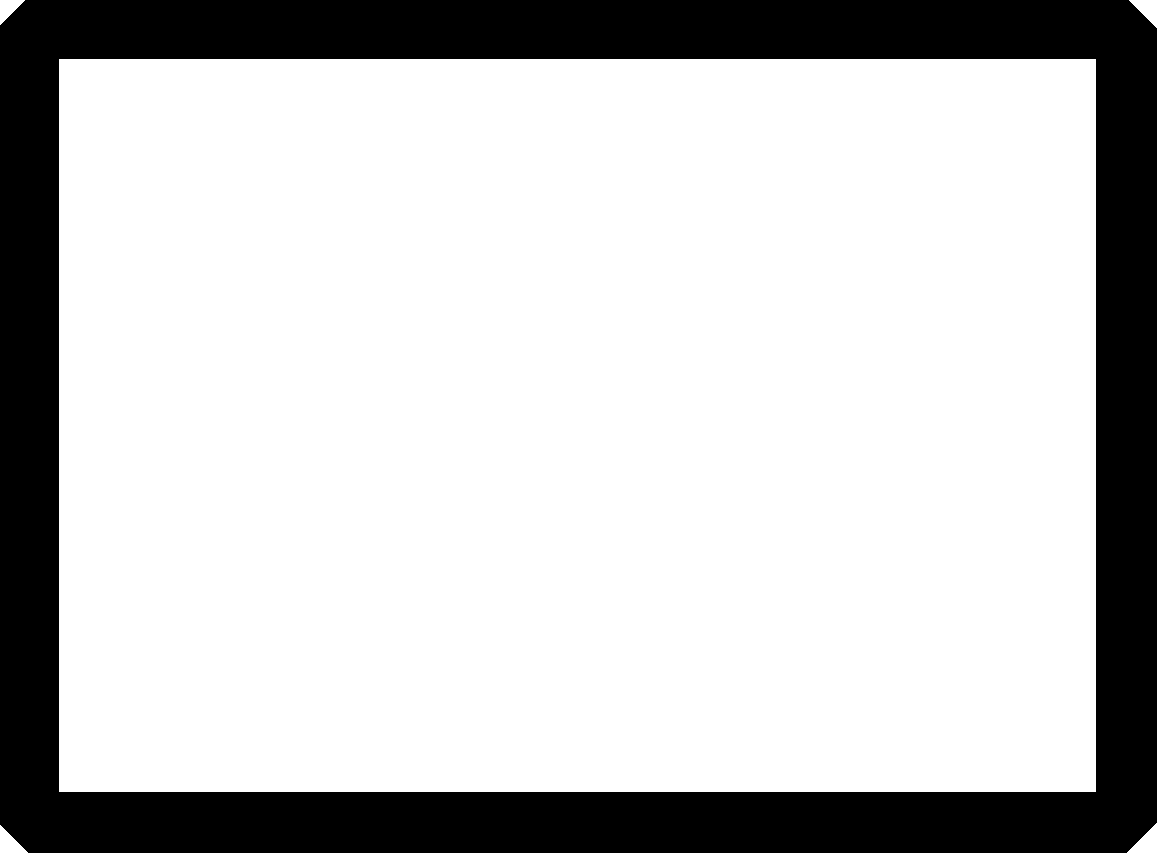 Privately owned – subsidized |
| 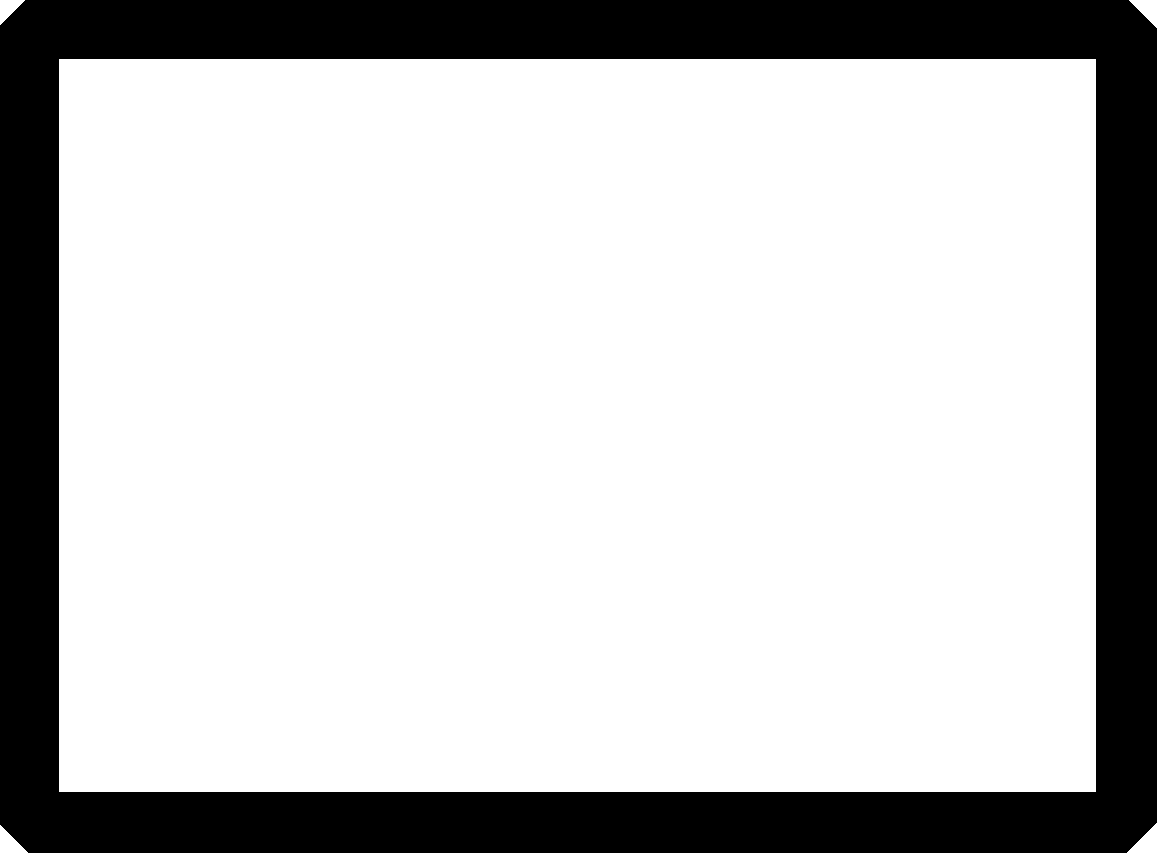 Privately owned – not subsidized |
| 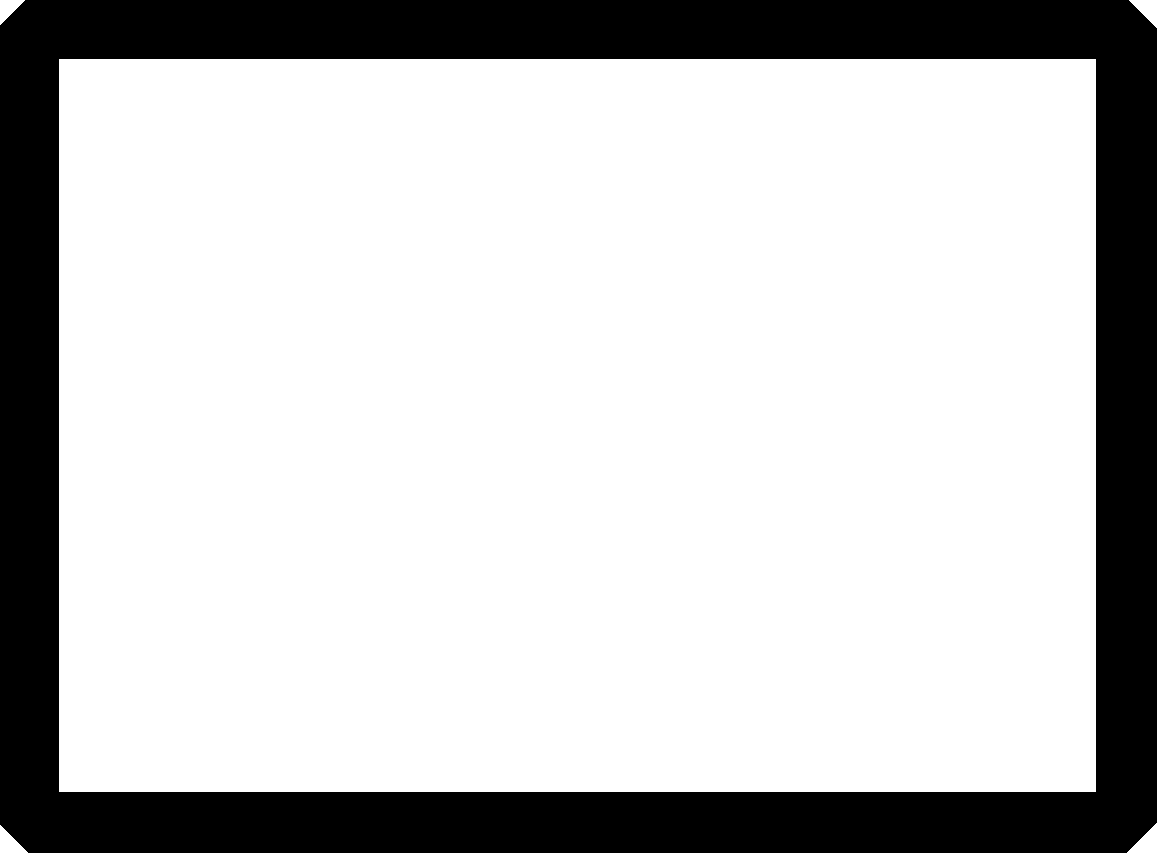 I don't know |

| **A8.**  Have you received prior training in digital competencies/skills? |
| --- |
| 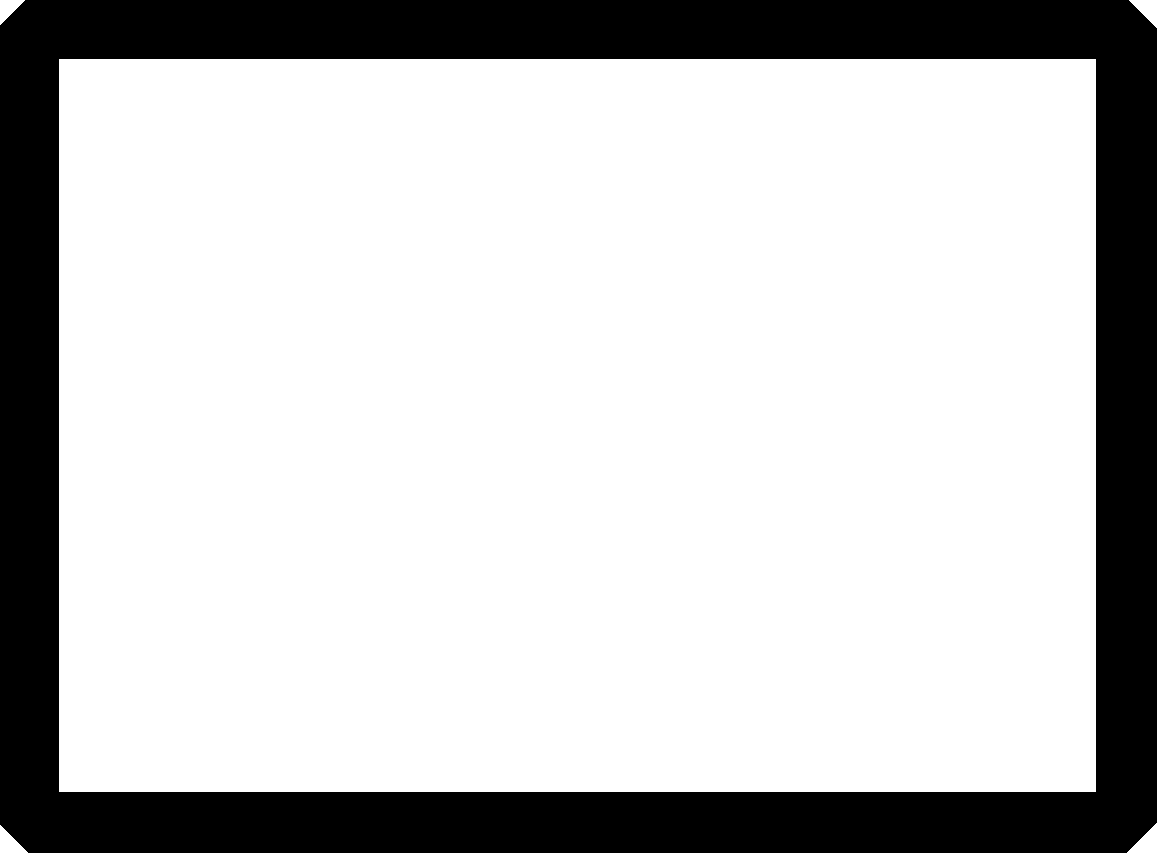 Yes |
| 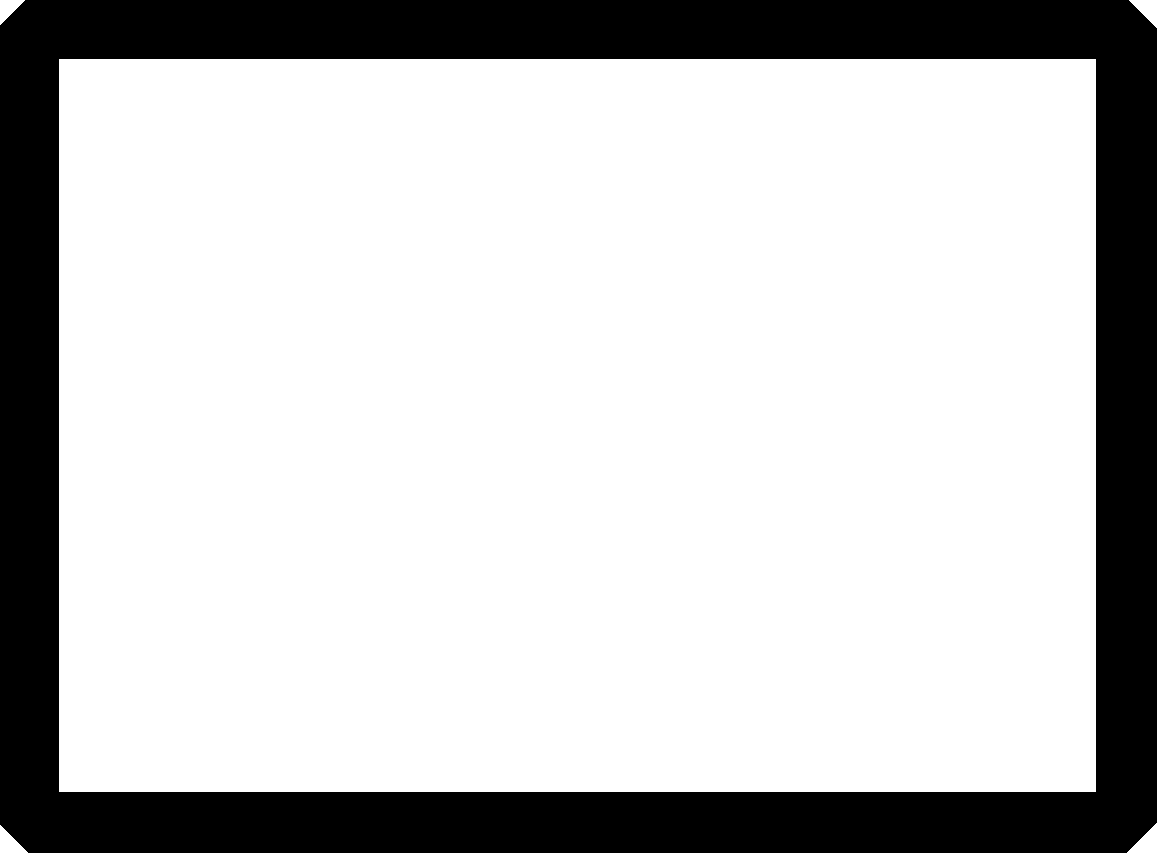 No |

**BLOCK III: TRAINING NEEDS**

**B1.** Digital skills in Information. To what extent is training in the following digital information skills necessary for cancer care? Please indicate your opinion on a scale from 1 to 7 (1 being *Absolutely not necessary* and 7 being *Absolutely necessary*).

|  | **Absolutely not necessary**  **[1]** | **[2]** | **[3]** | **[4]** | **[5]** | **[6]** | **Absolutely necessary**  **[7]** |
| --- | --- | --- | --- | --- | --- | --- | --- |
| **B1a.** Search for information on the Internet using a search engine (e.g., Google, Bing, Yahoo!...). | 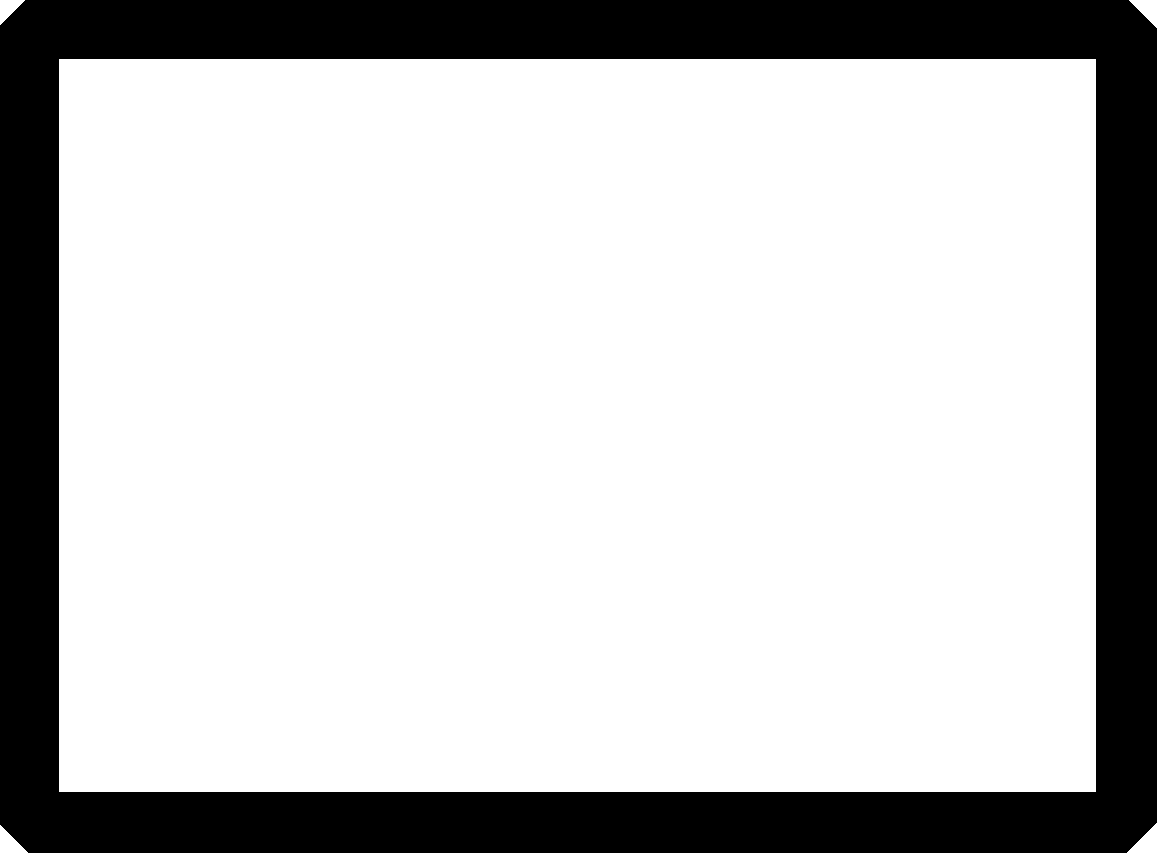 | 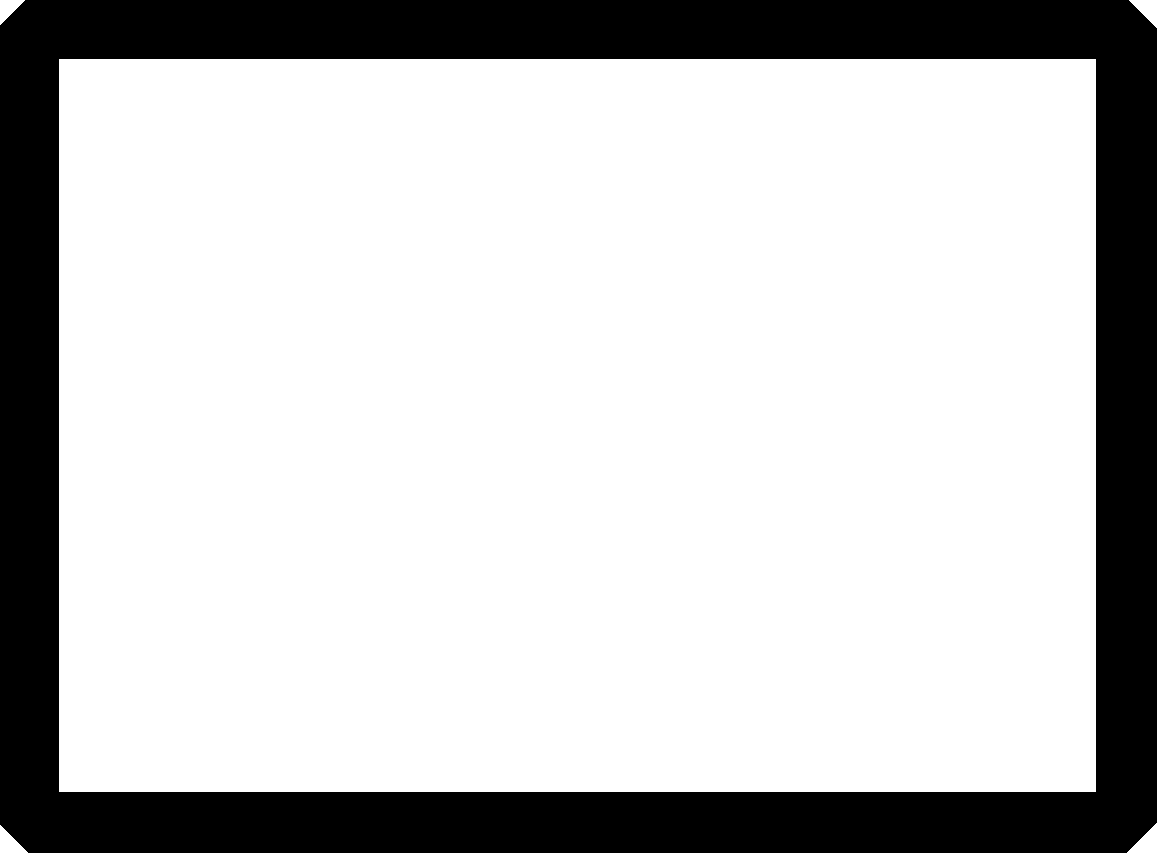 | 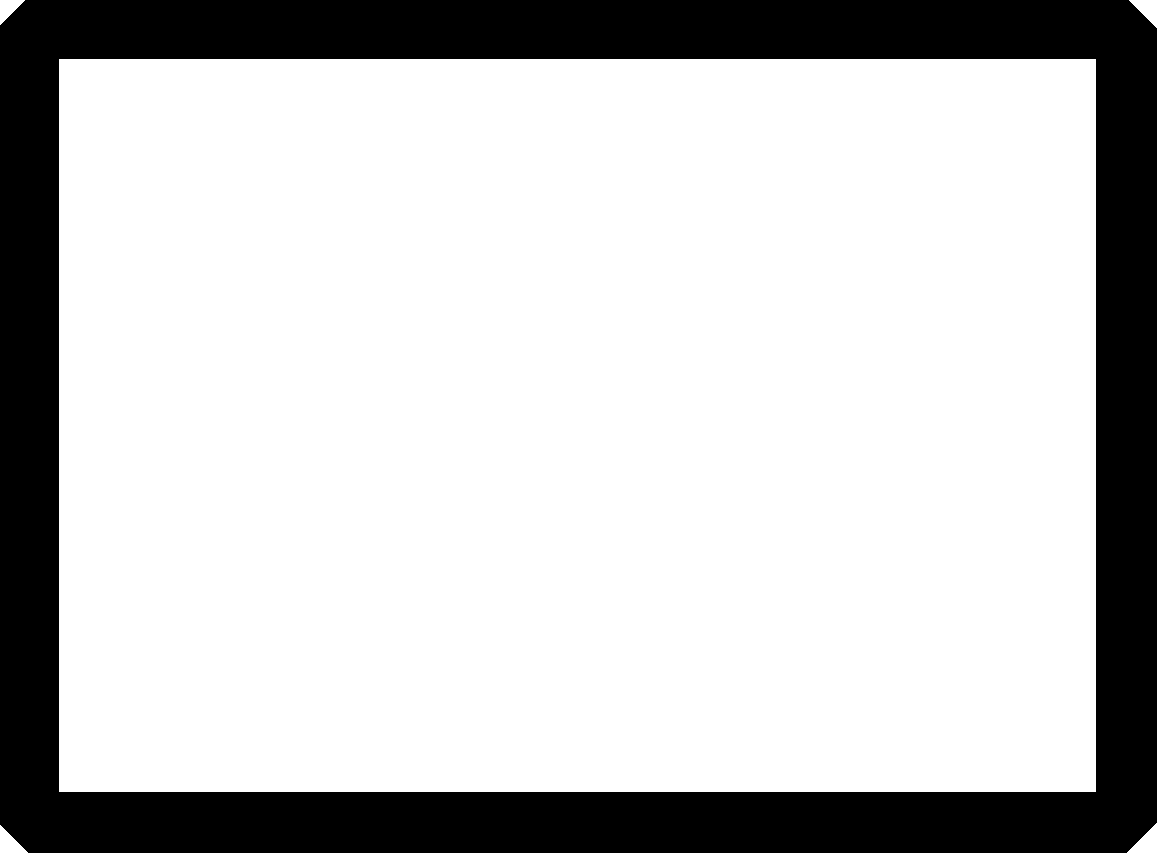 | 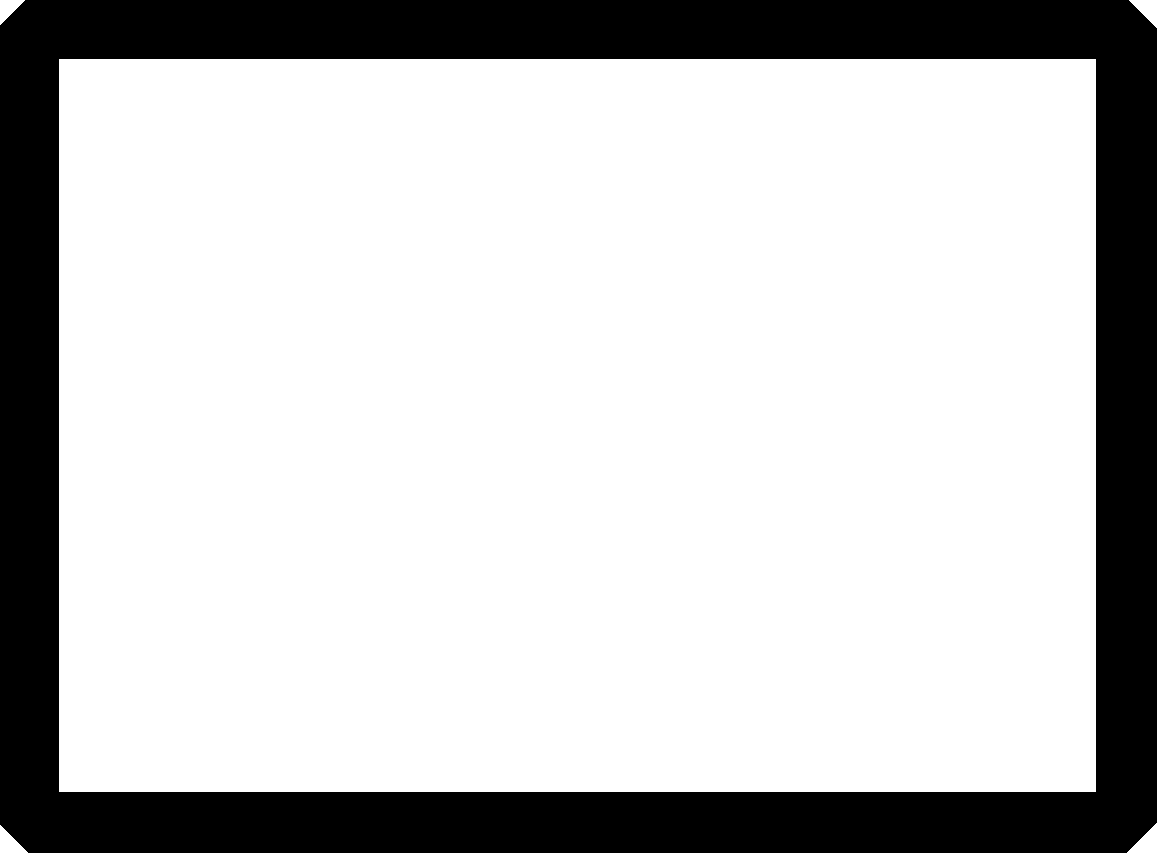 | 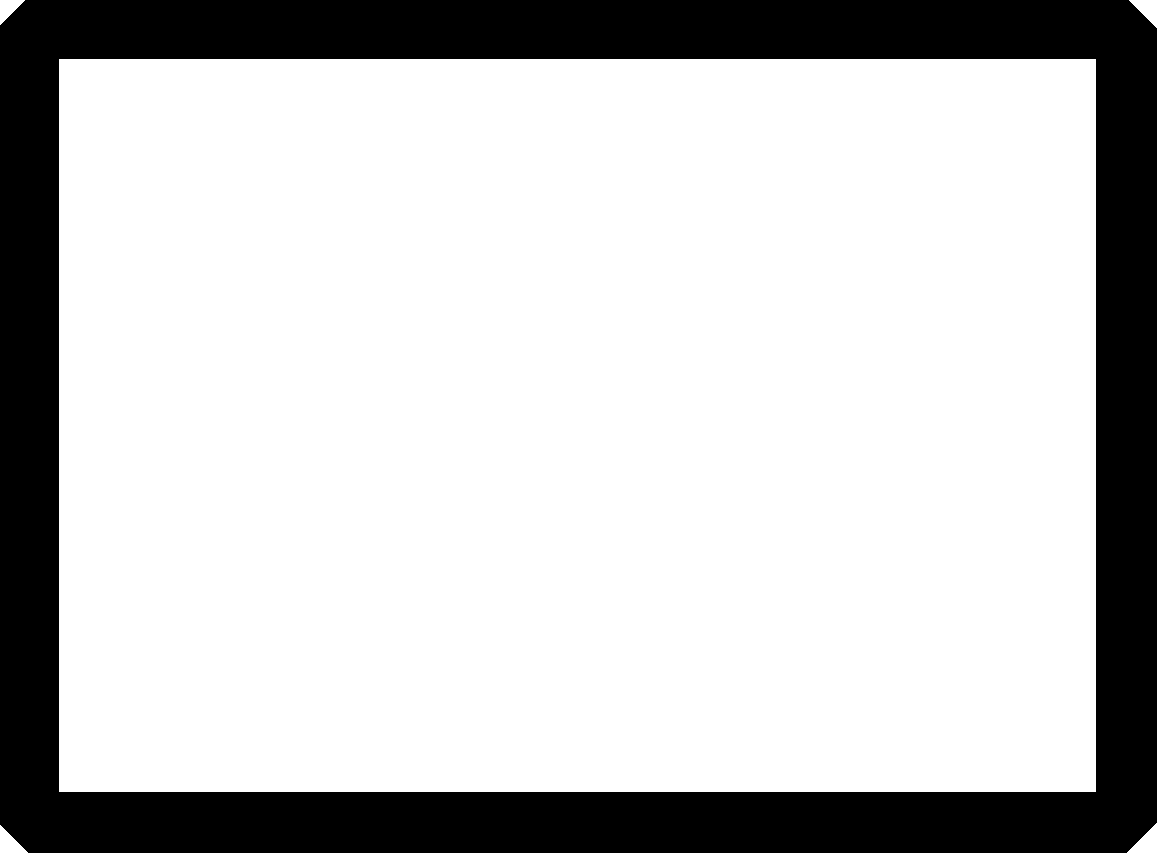 | 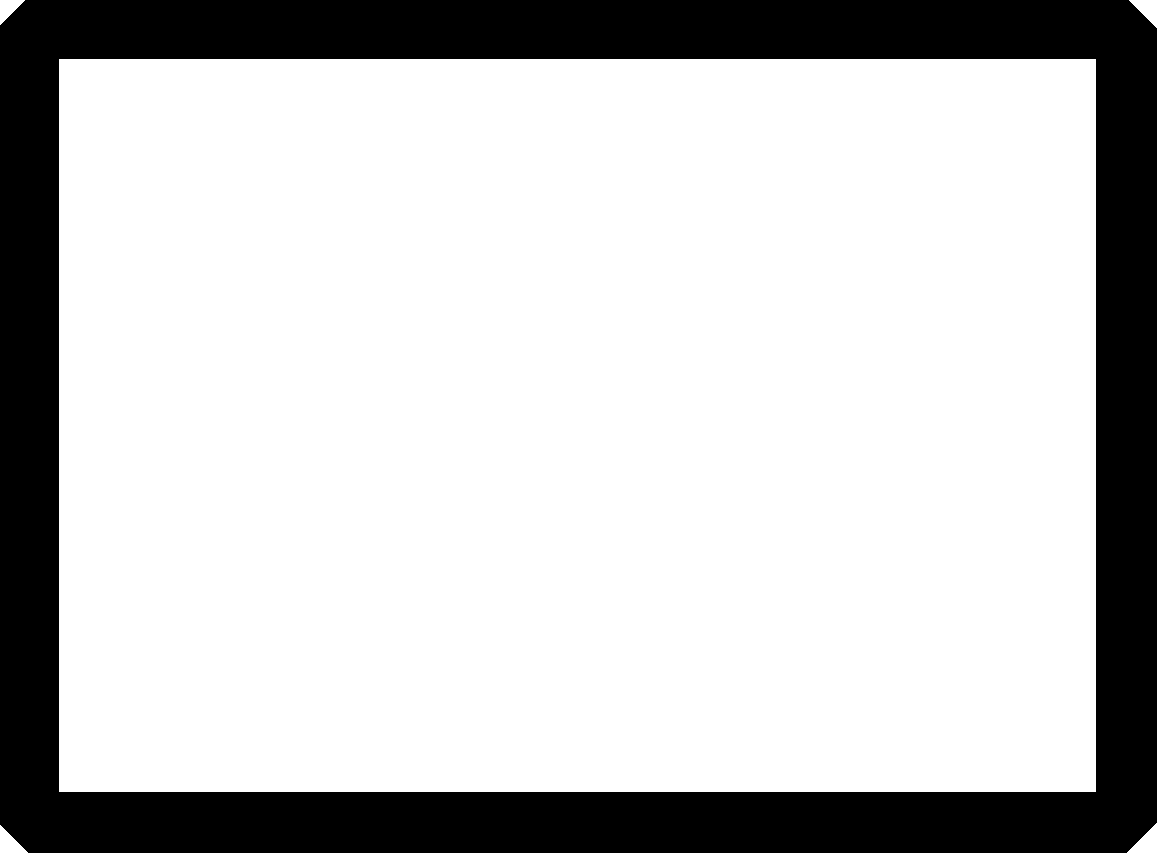 | 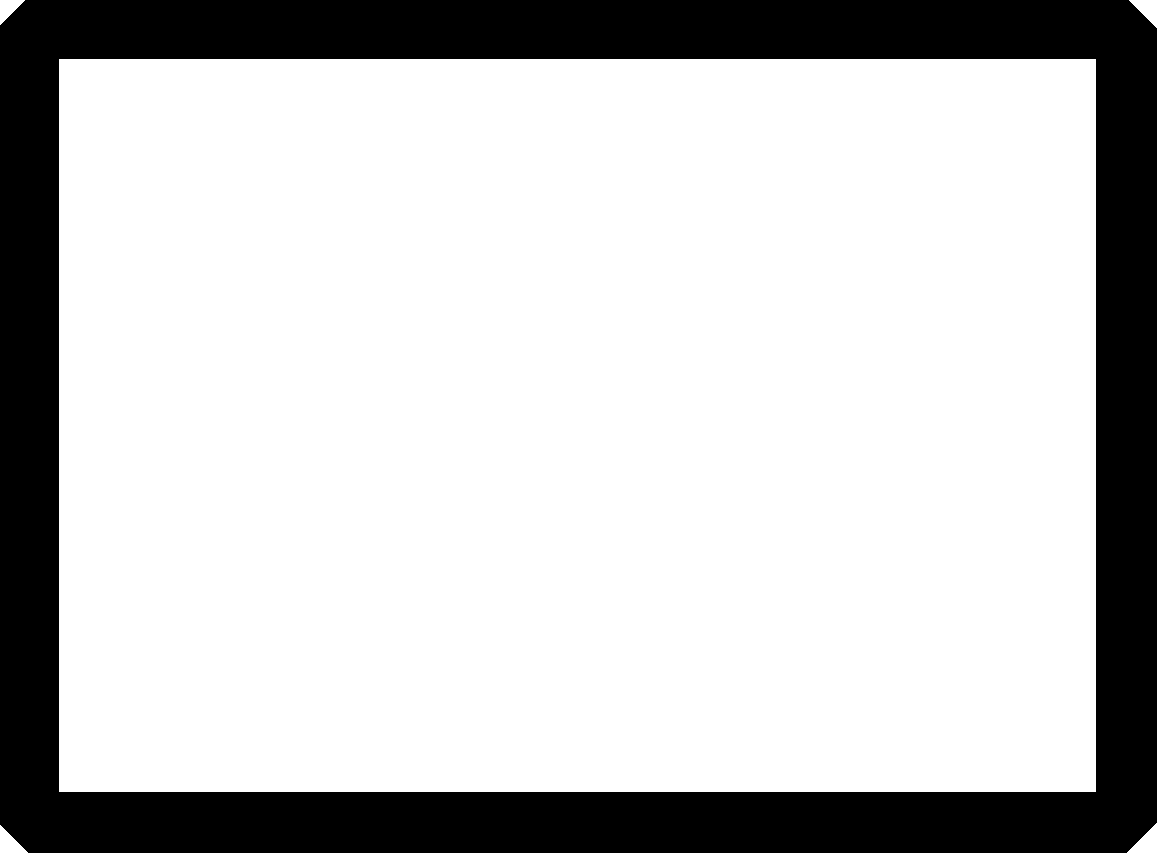 |
| **B1b.** Differentiate between reliable and unreliable online information. | 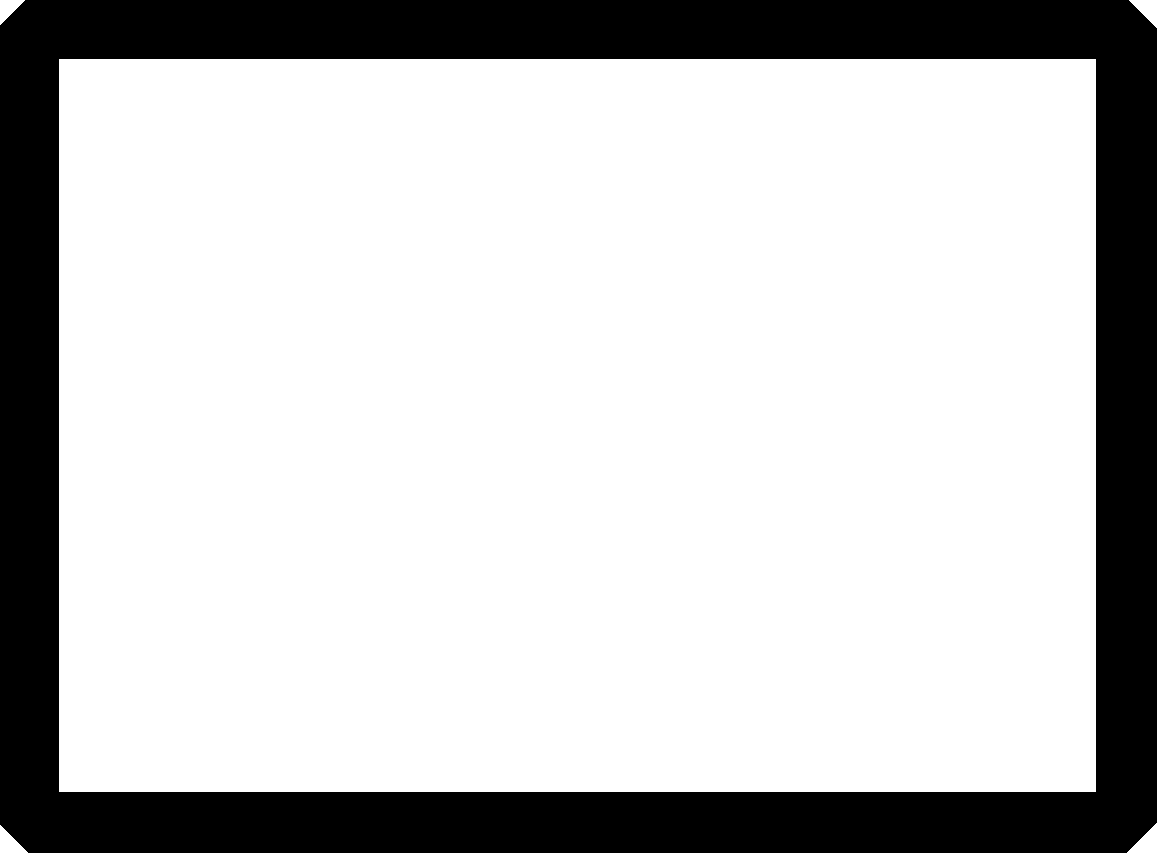 | 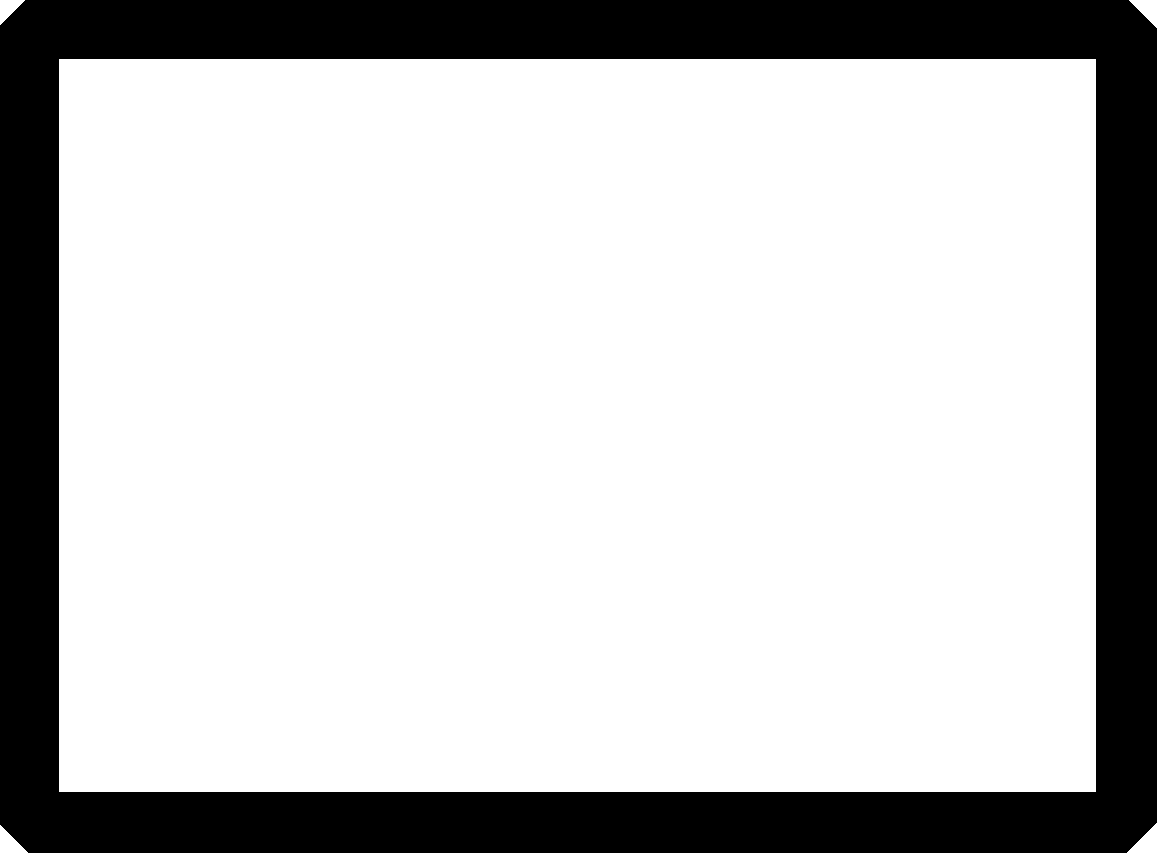 | 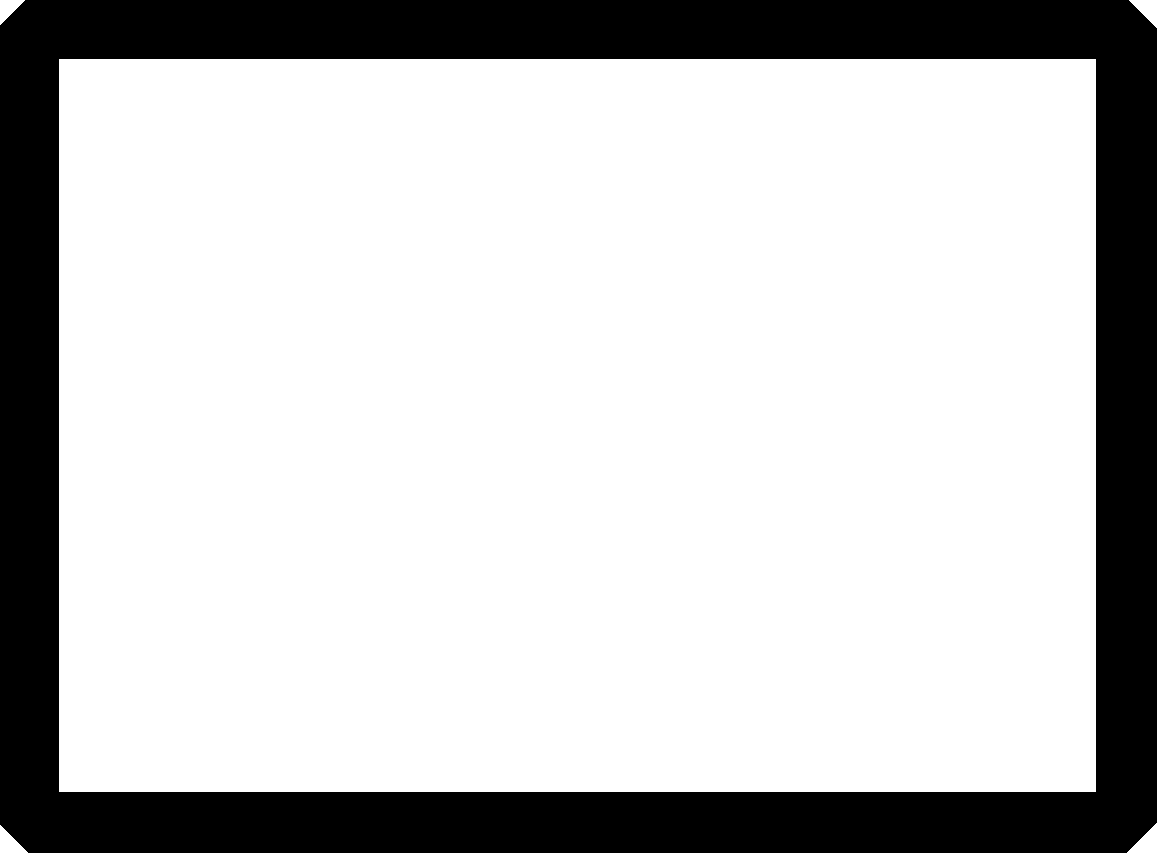 | 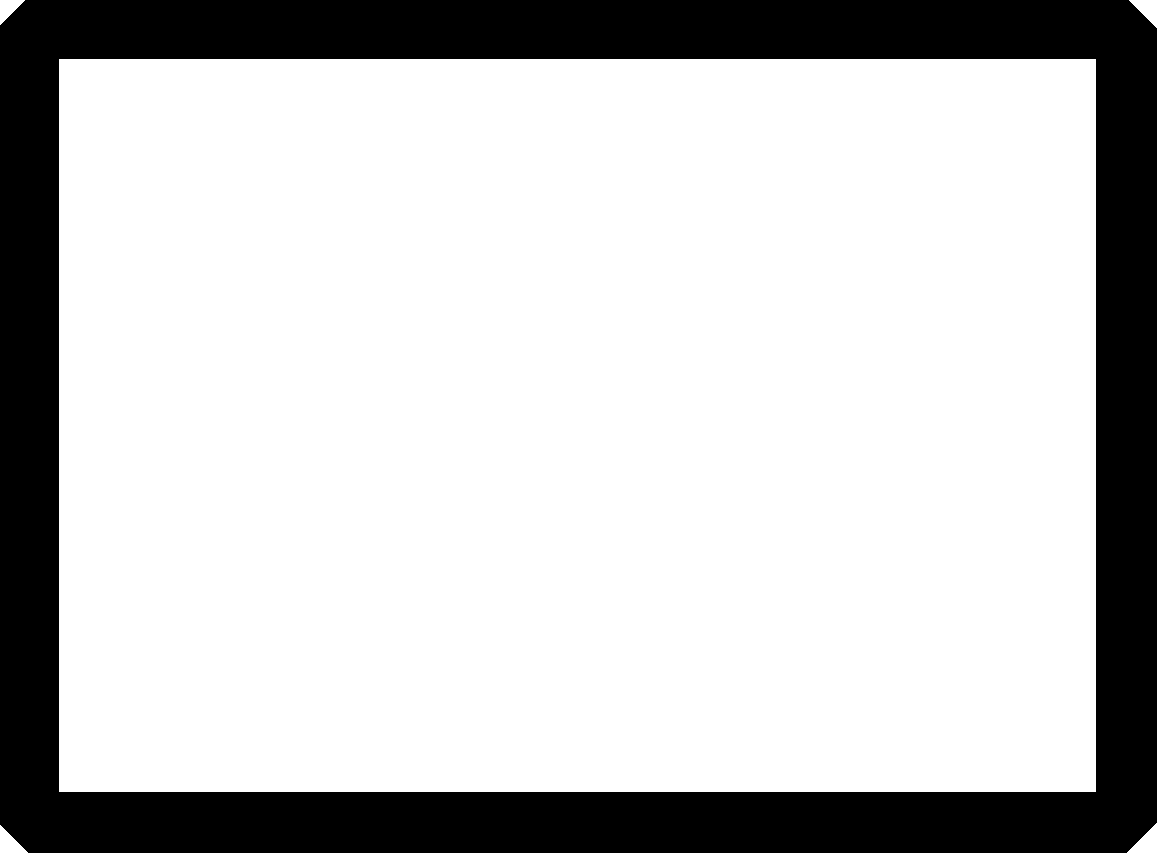 | 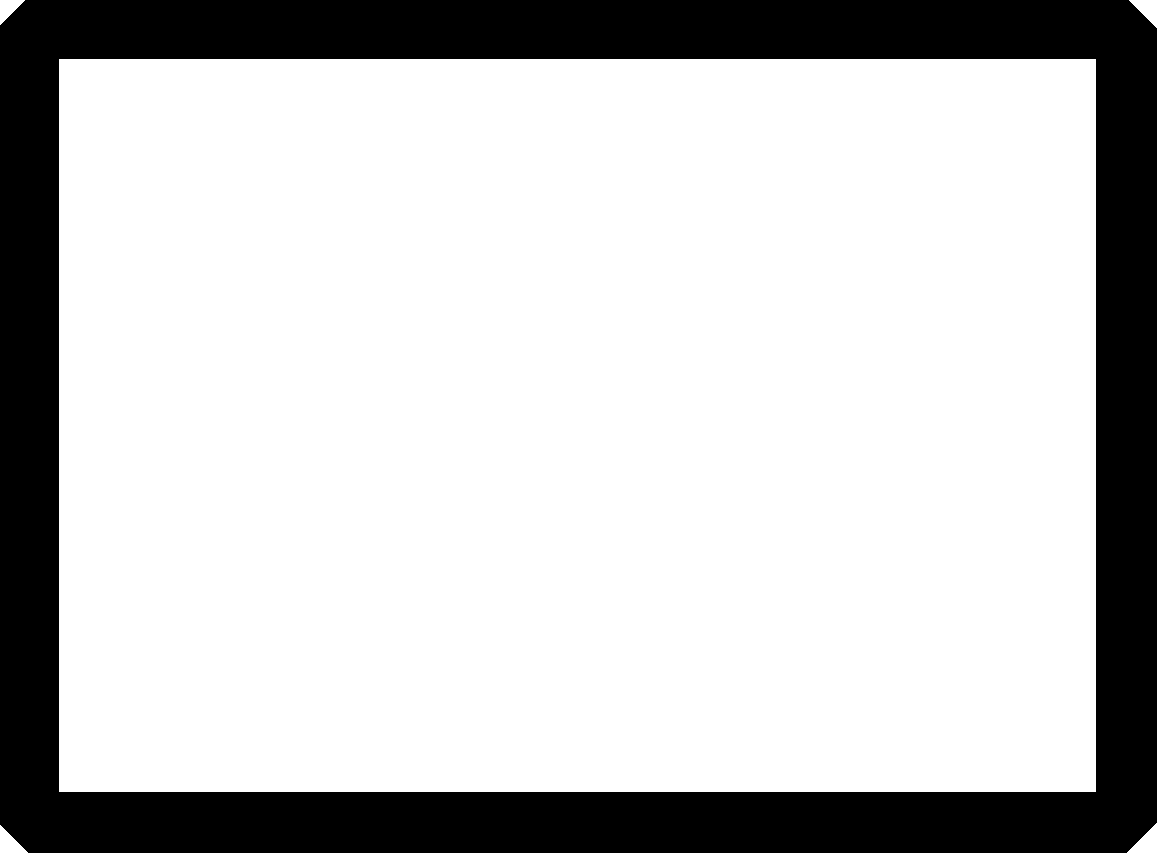 | 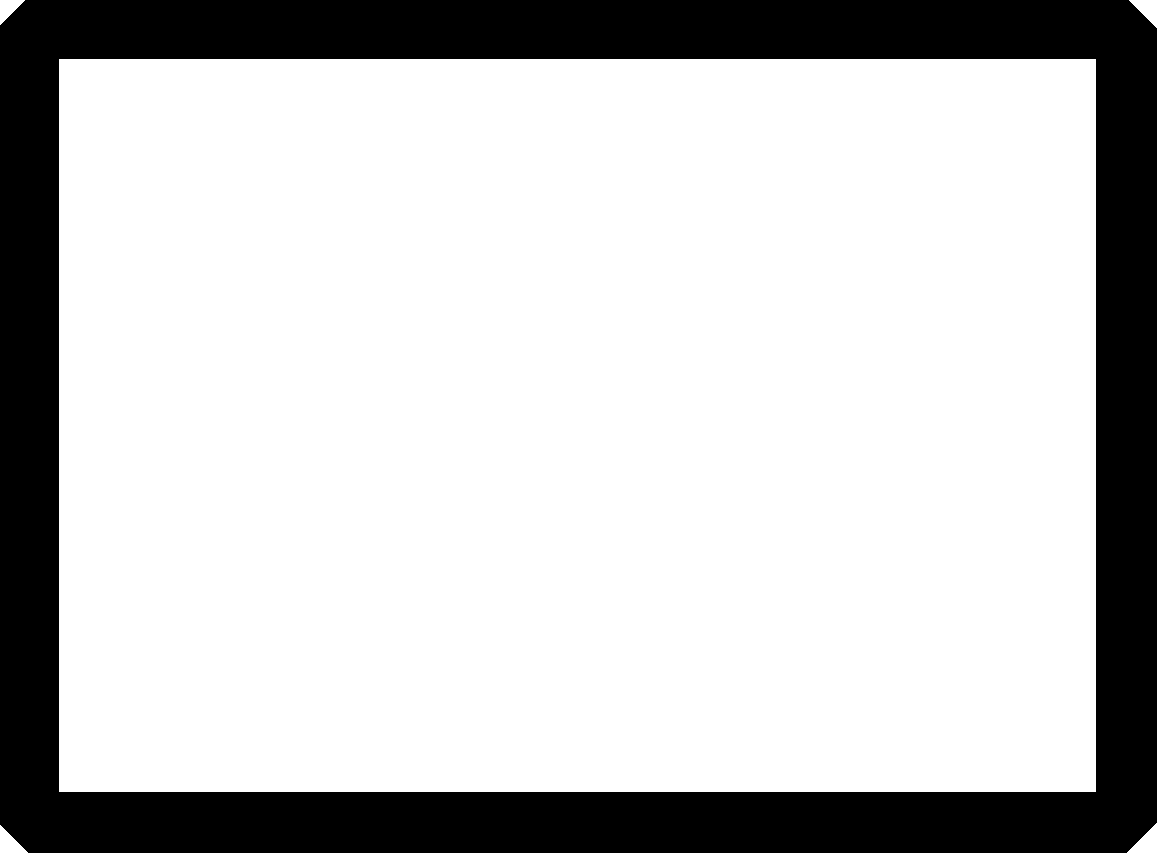 | 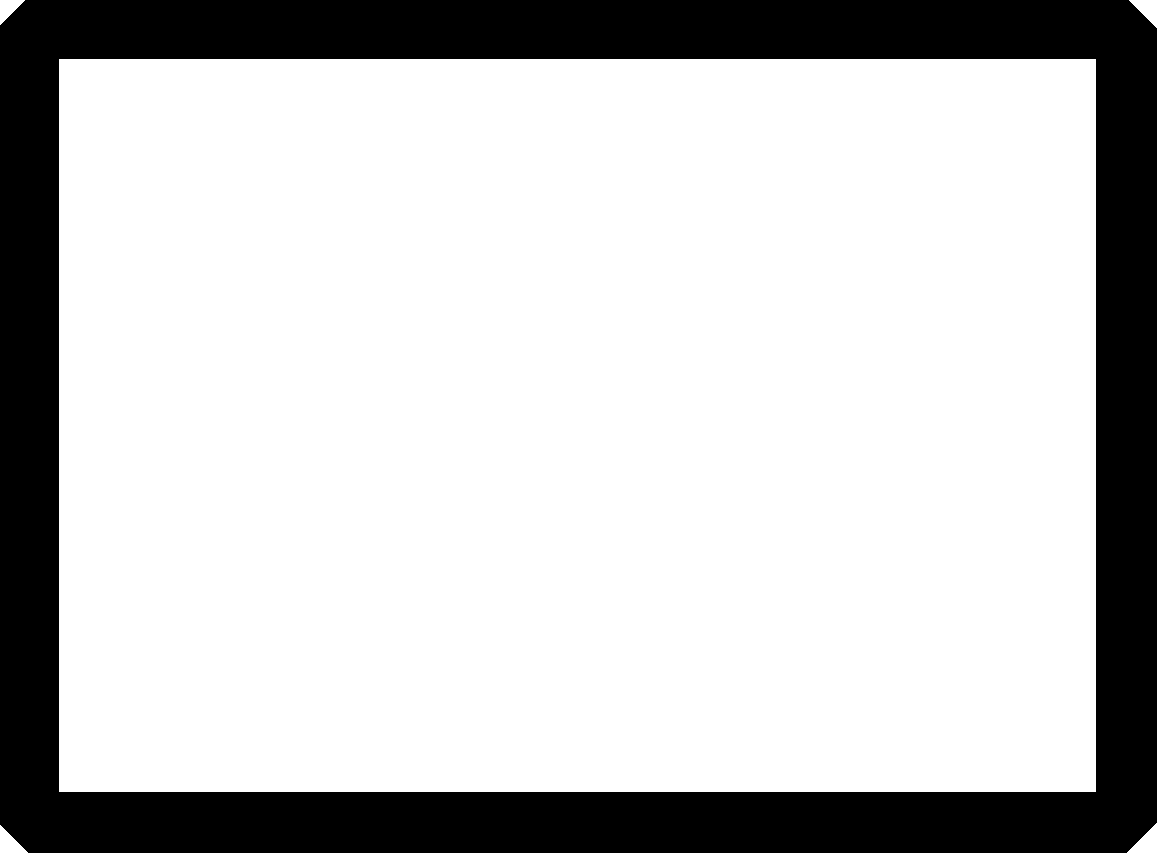 |
| **B1c.** Save or store files or content (e.g., text, images, music, videos, web pages) and retrieve them once saved or stored. | 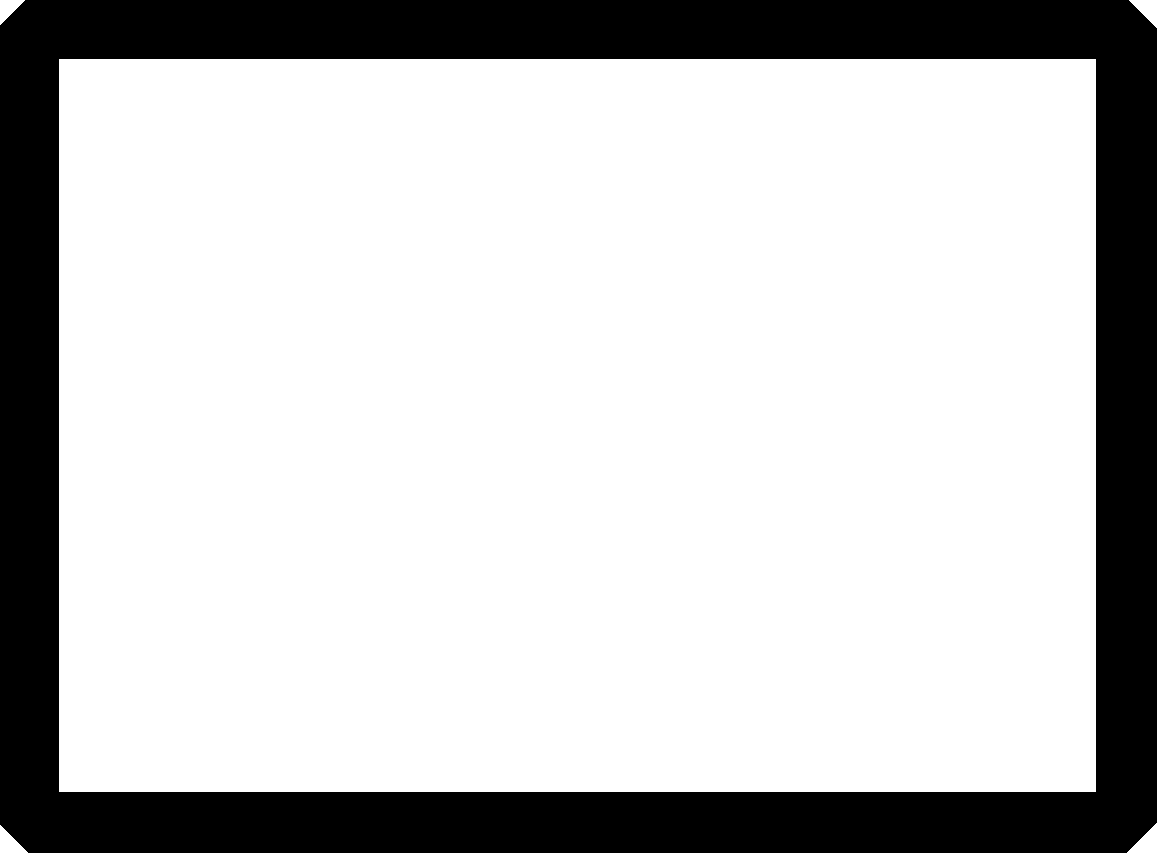 | 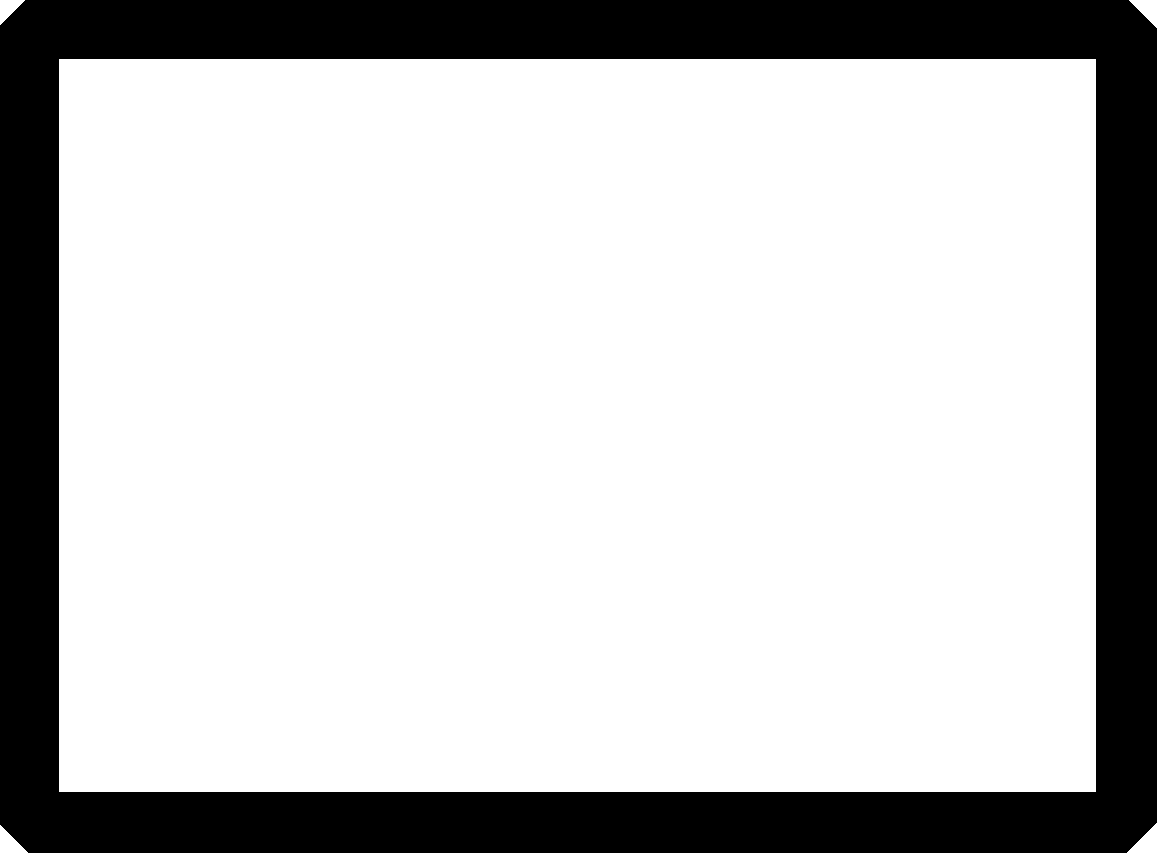 | 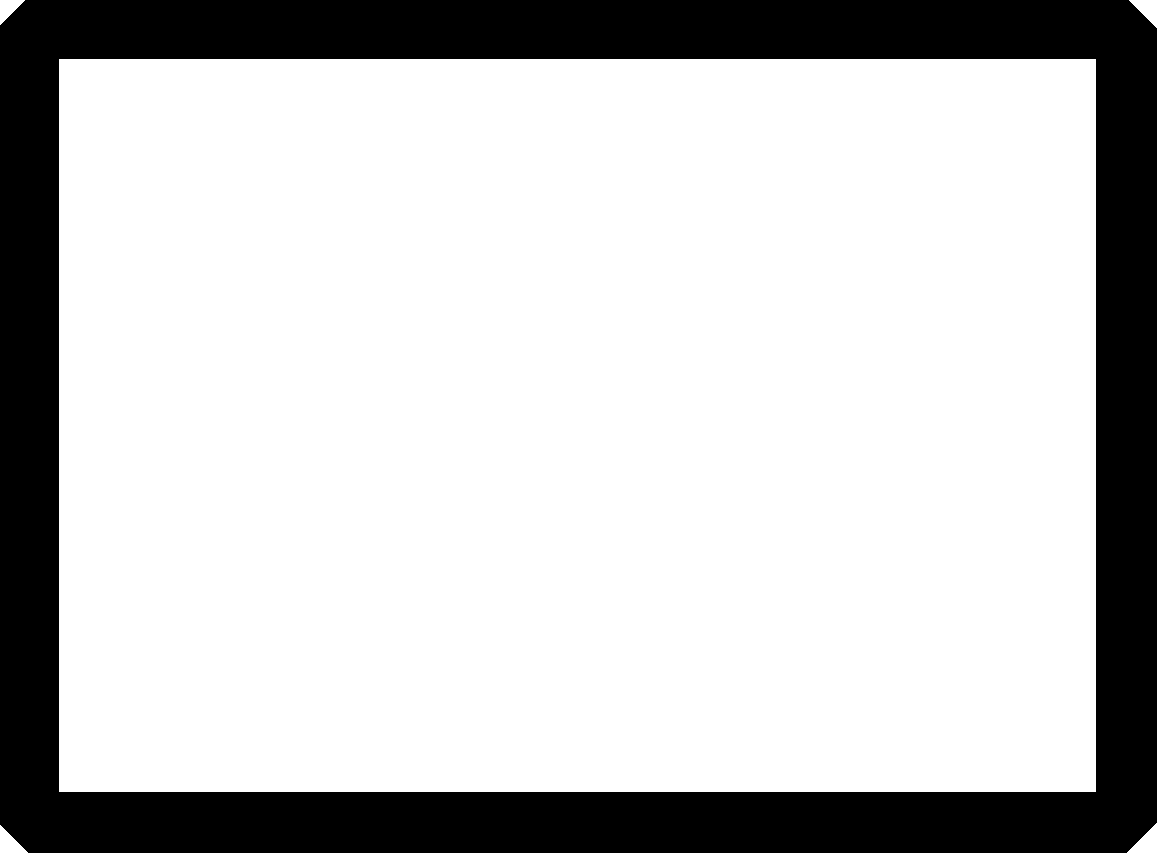 | 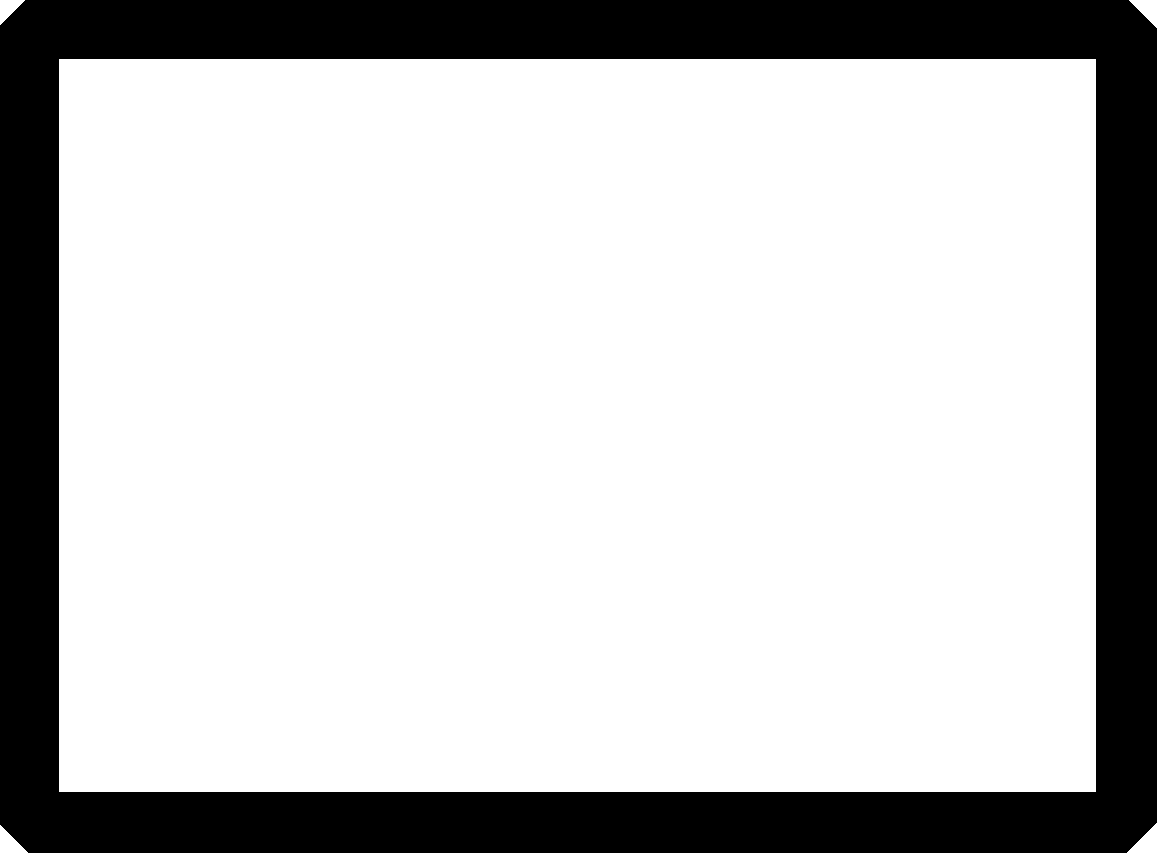 | 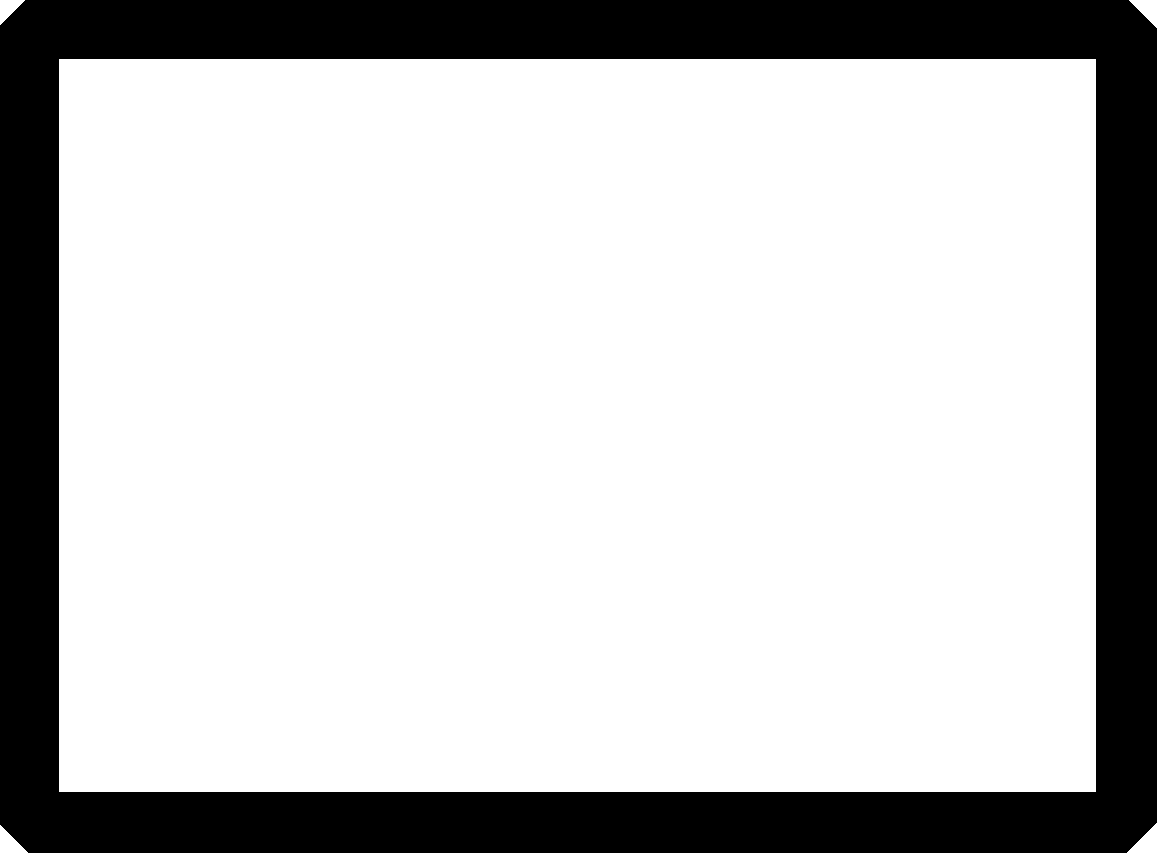 | 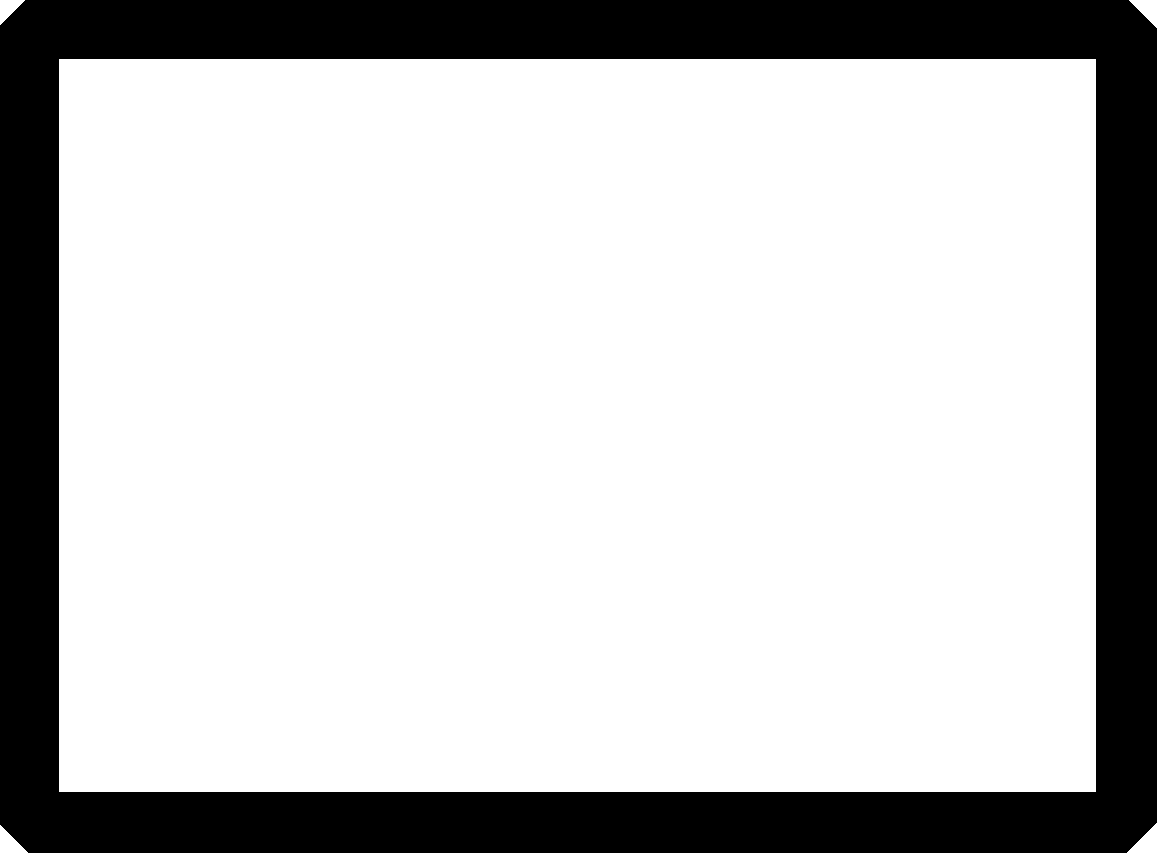 | 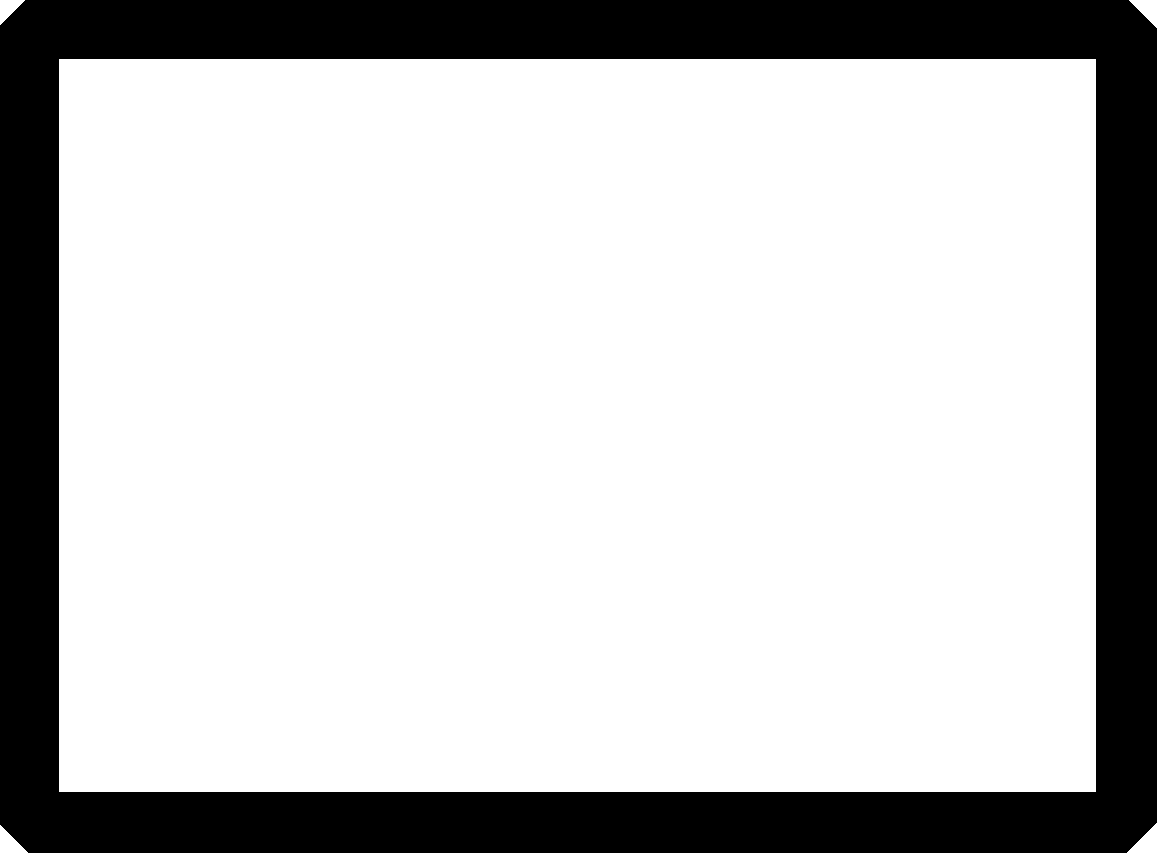 |
| **B1d.** Use the electronic health record for the storage of health data. | 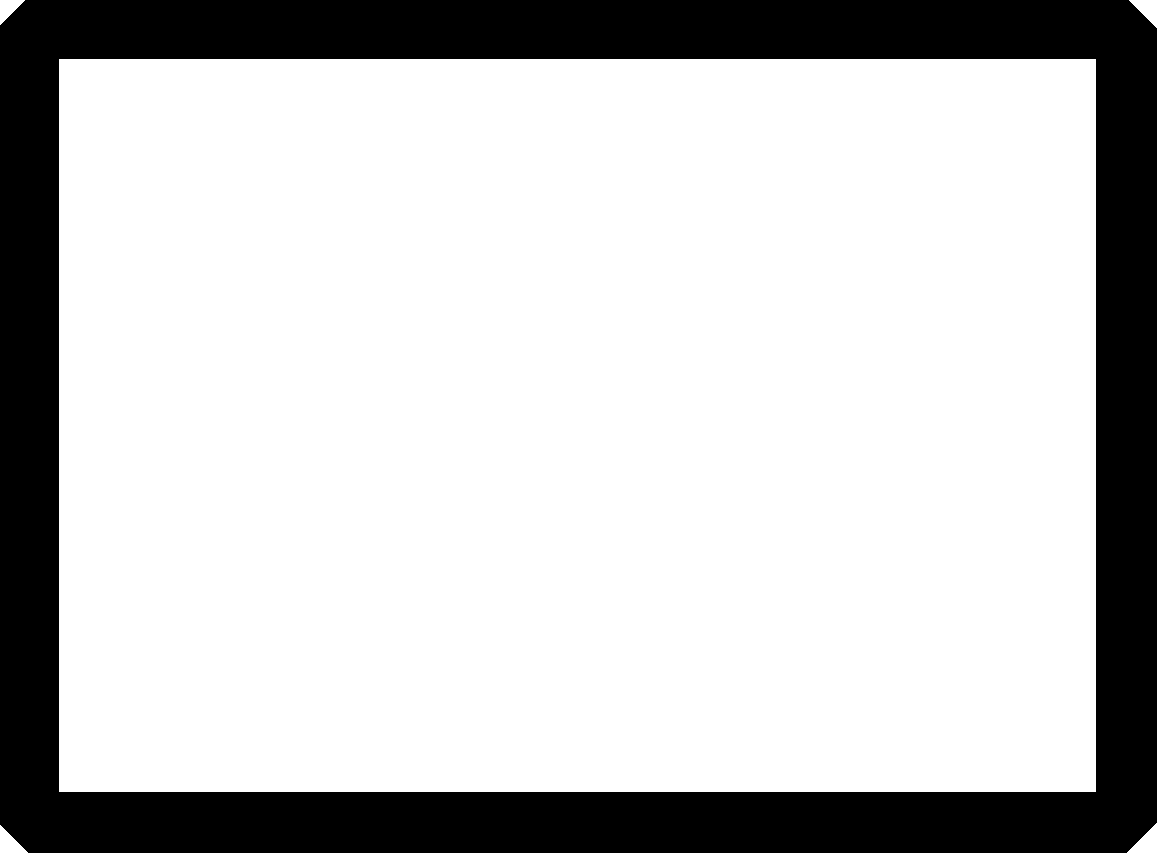 | 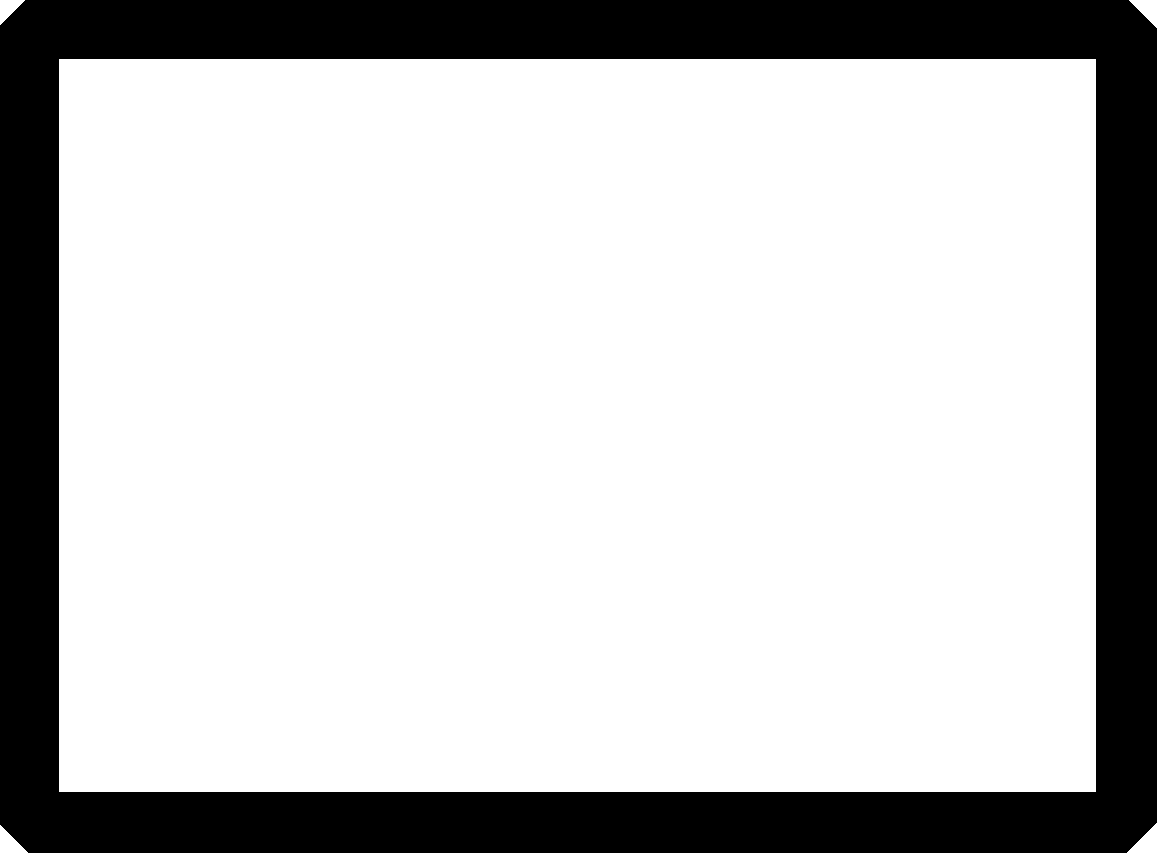 | 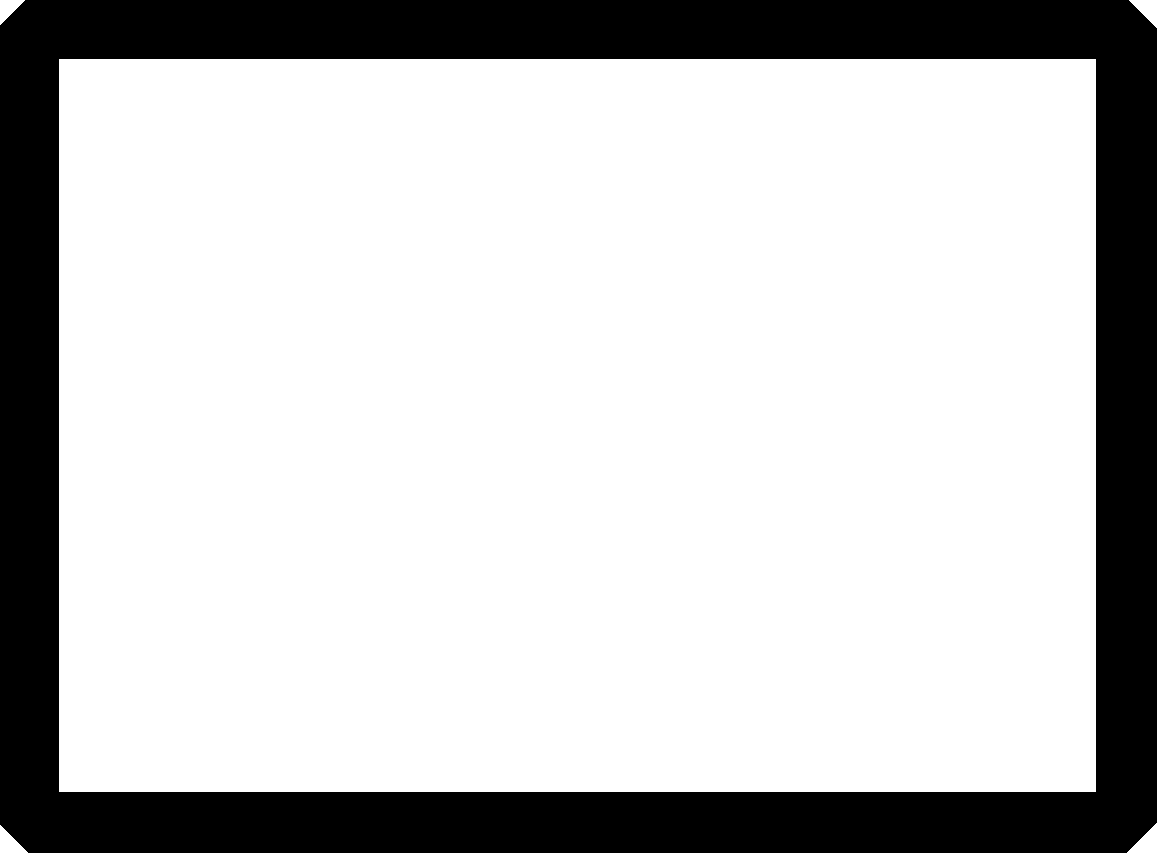 | 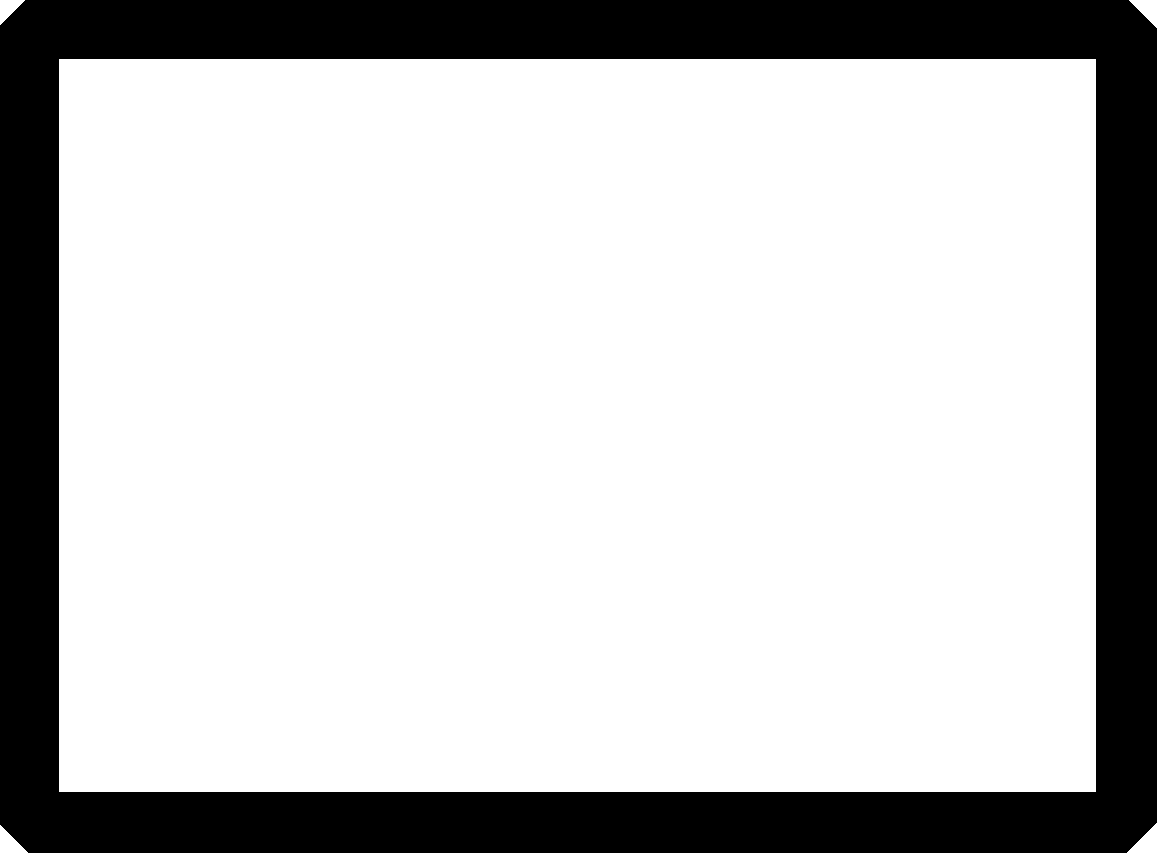 | 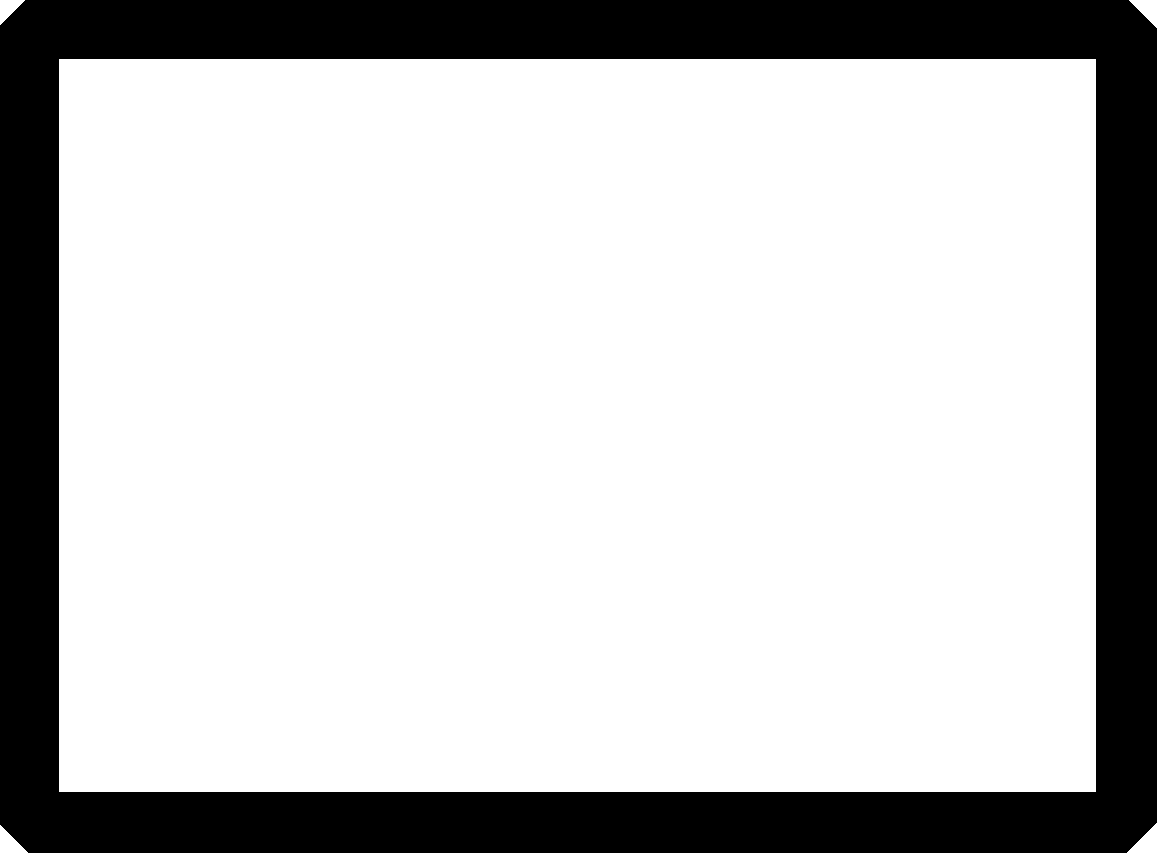 | 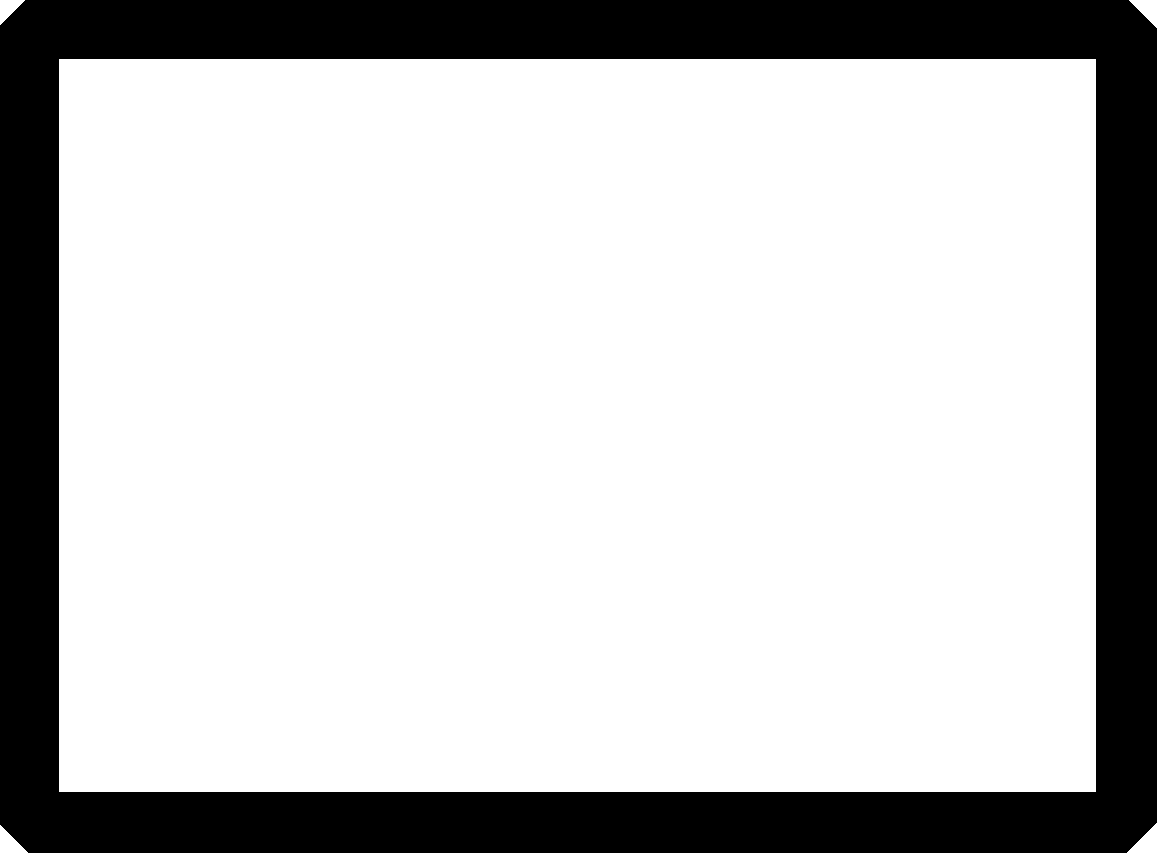 | 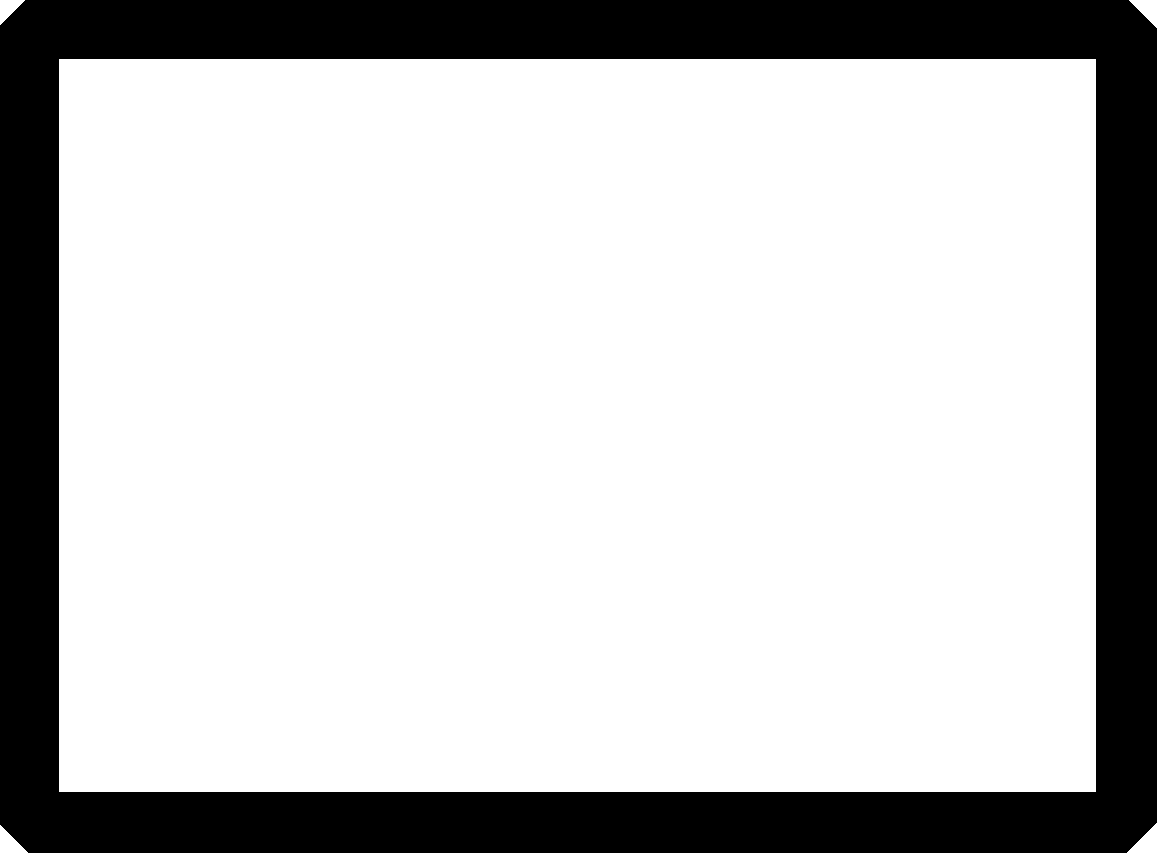 |
| **B1e.** Identify the advantages, disadvantages, risks and opportunities of data exchange through electronic systems. | 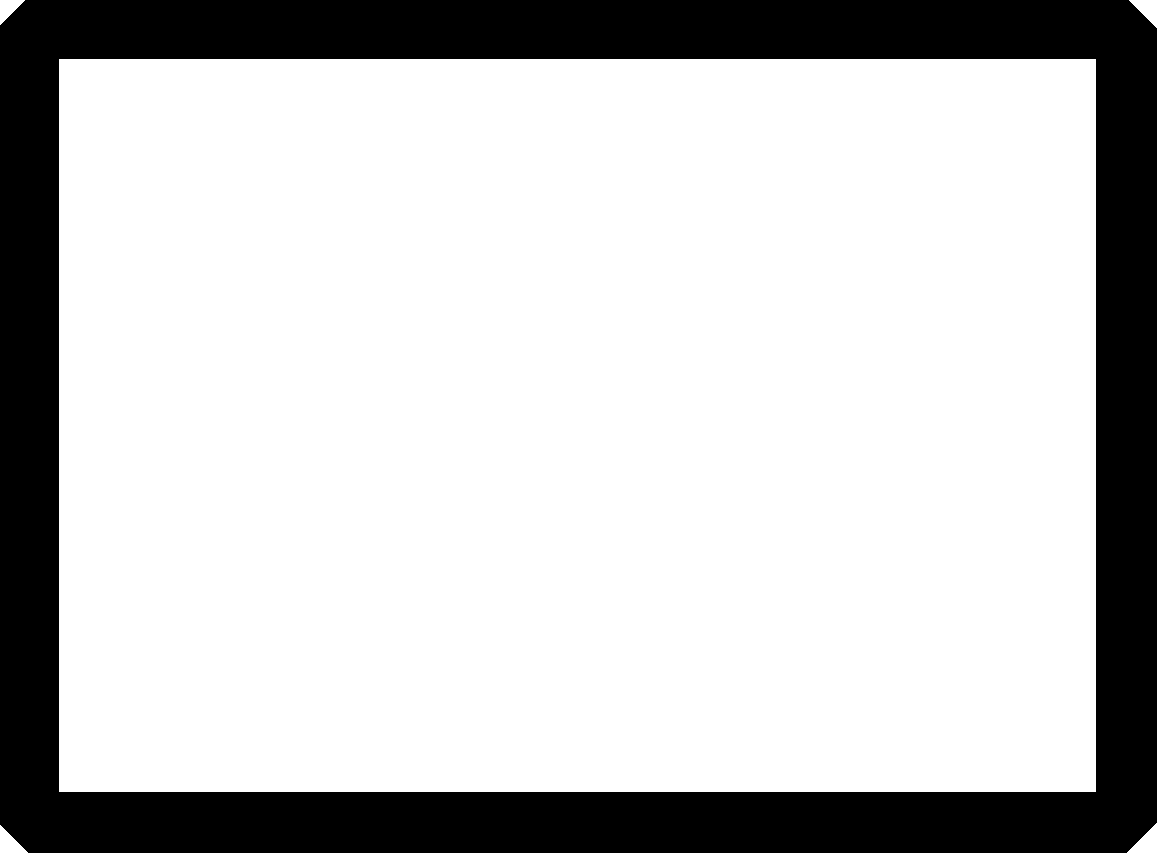 | 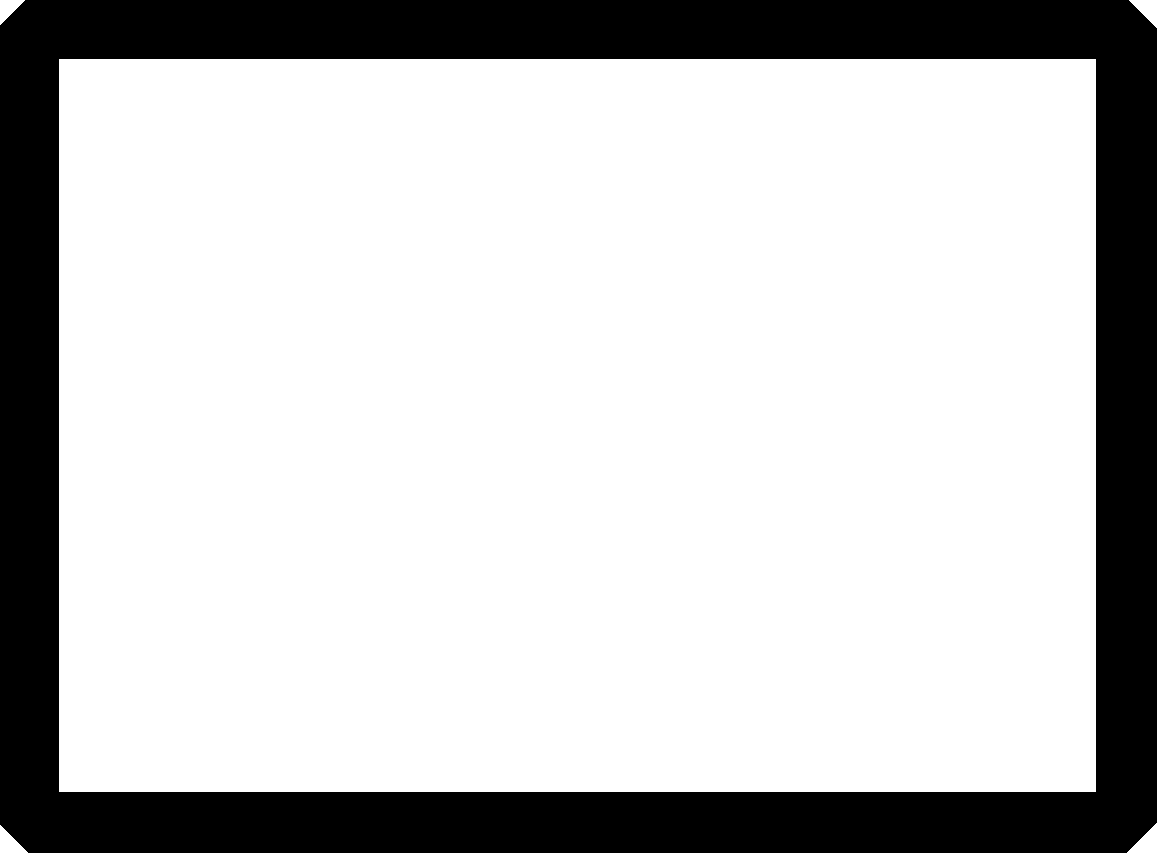 | 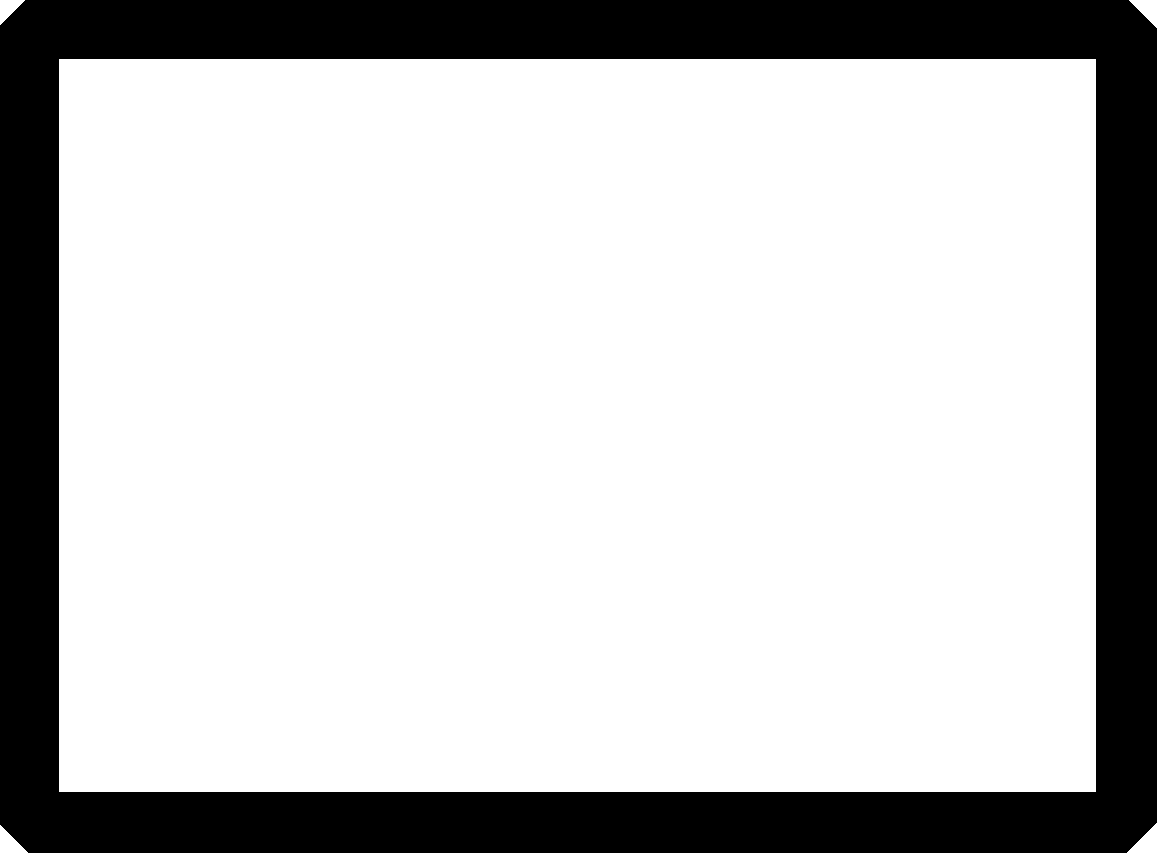 | 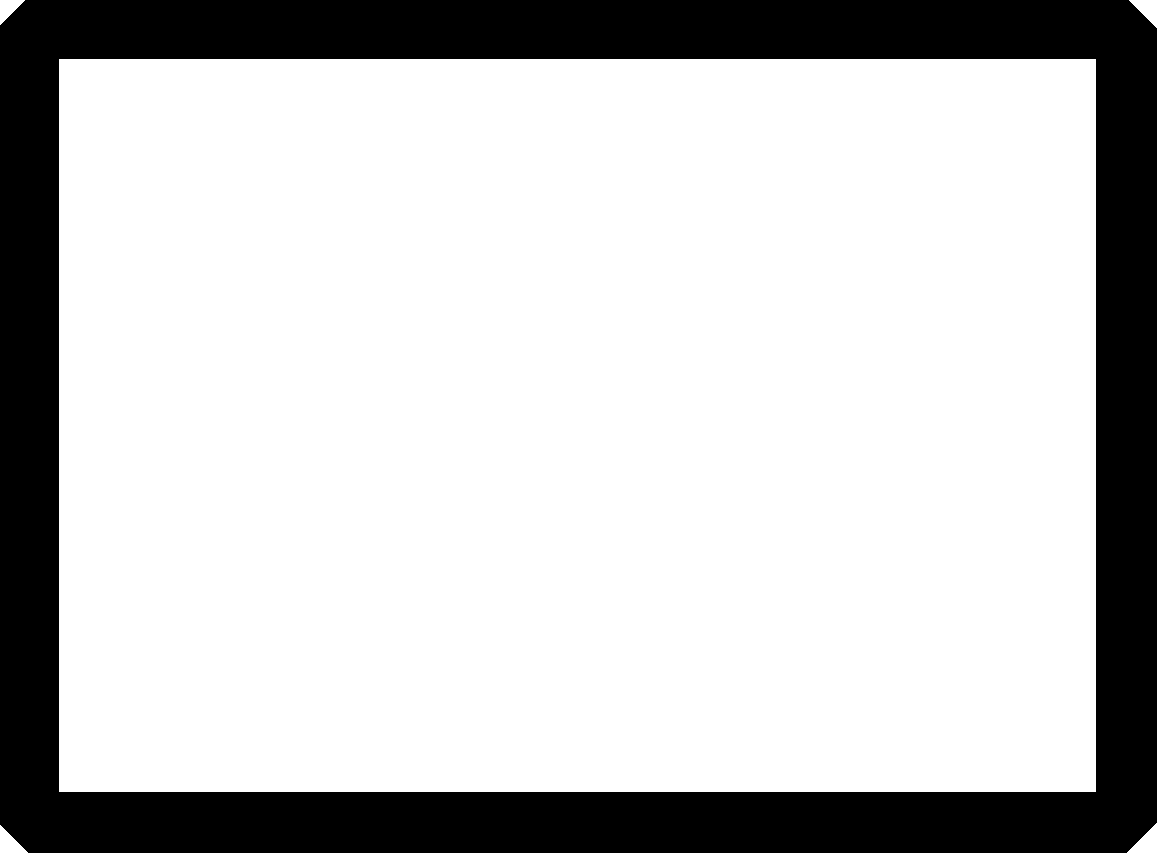 | 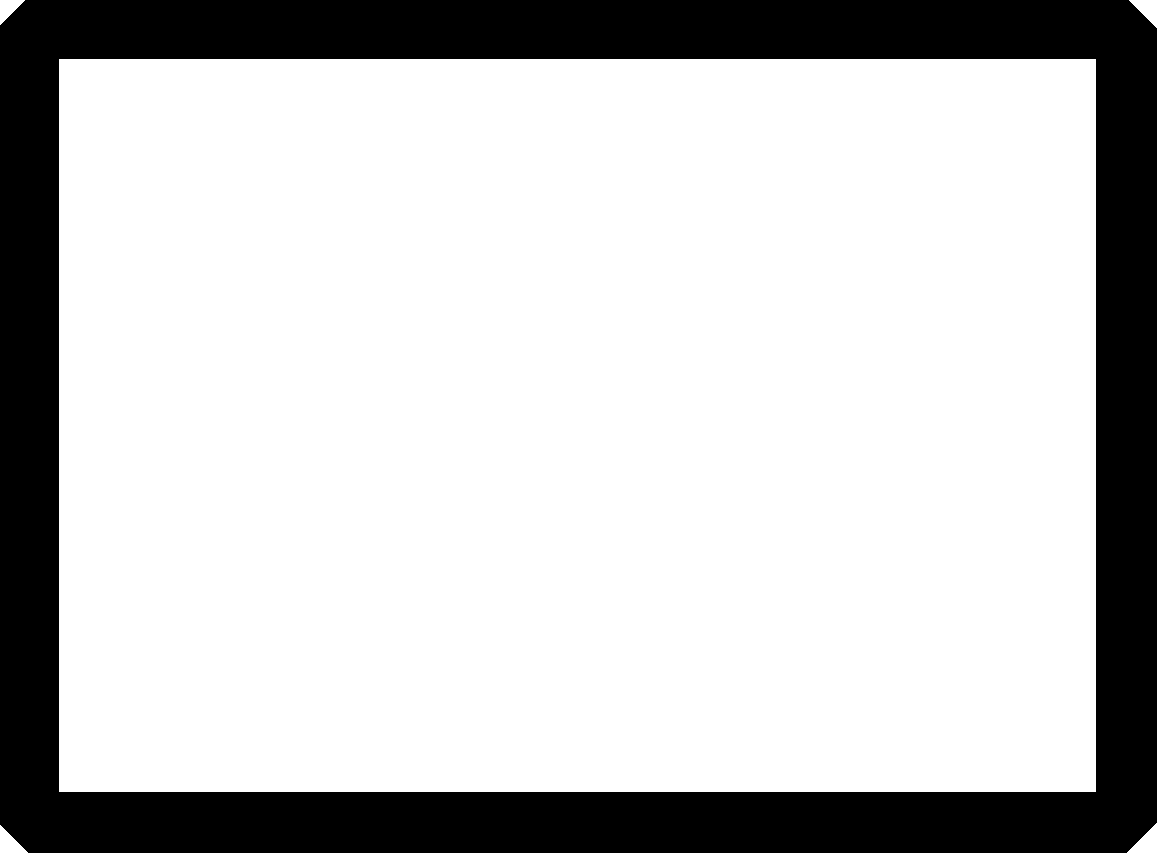 | 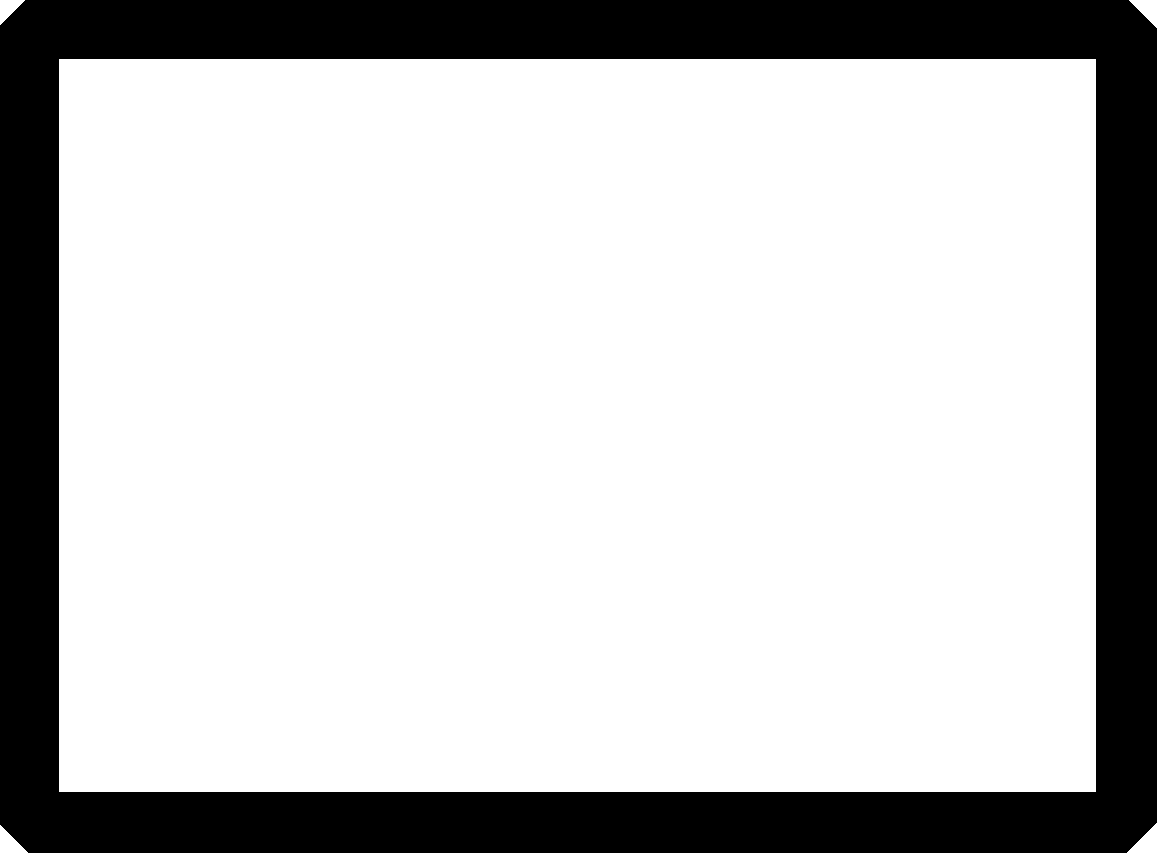 | 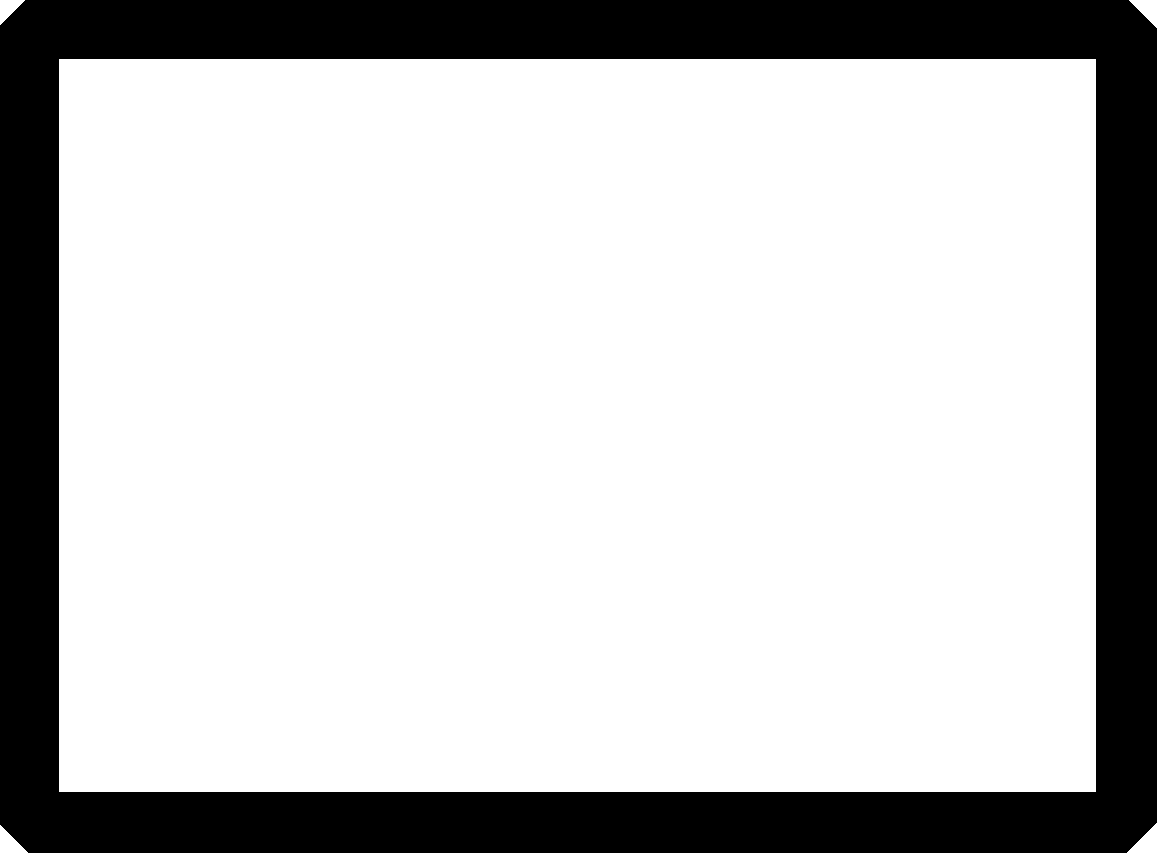 |

**B2.** Digital skills in Communication. To what extent is training in the following digital communication skills necessary for cancer care? Please indicate your opinion on a scale from 1 to 7 (1 being *Absolutely not necessary* and 7 being *Absolutely necessary*).

|  | **Absolutely not necessary**  **[1]** | **[2]** | **[3]** | **[4]** | **[5]** | **[6]** | **Absolutely necessary**  **[7]** |
| --- | --- | --- | --- | --- | --- | --- | --- |
| **B2a.** Communicate with others using mobile phone, voice over IP (e.g., Skype), email or chat, using basic functions (e.g., voice messaging, SMS, sending and receiving email, text exchange). | 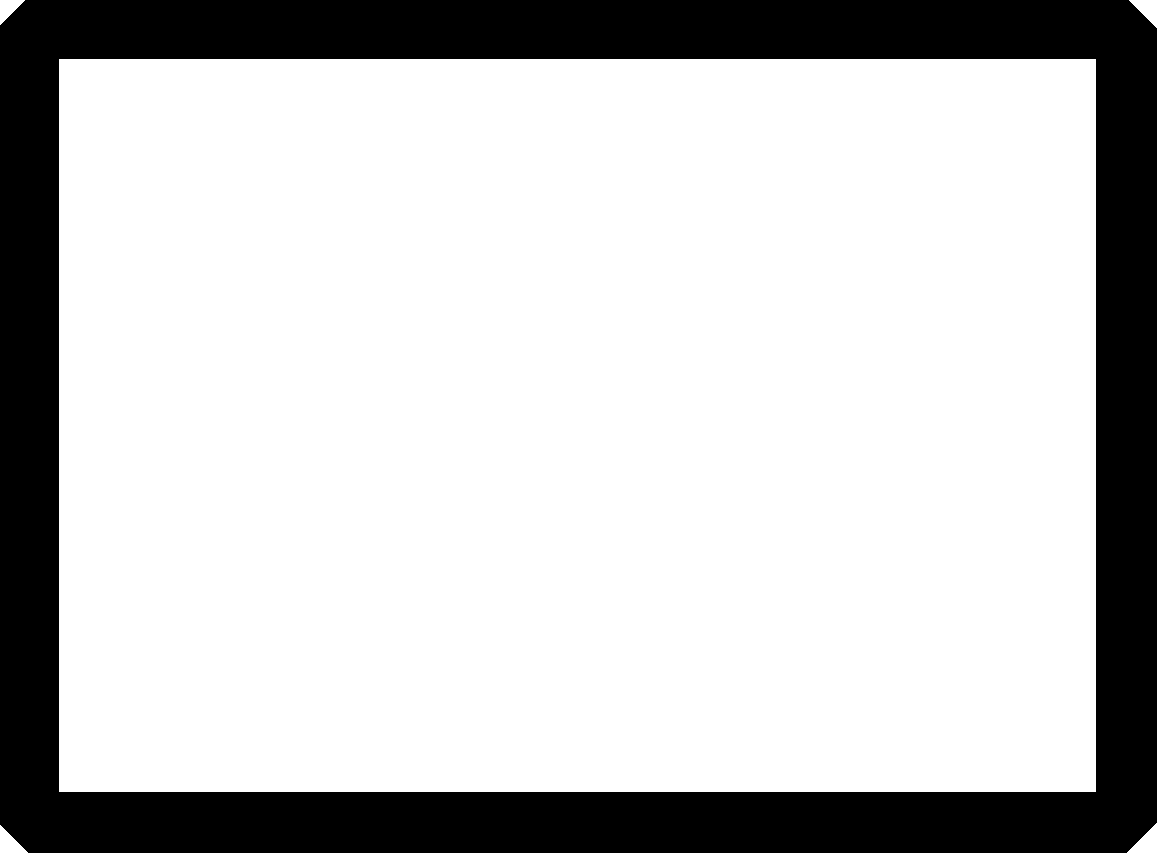 | 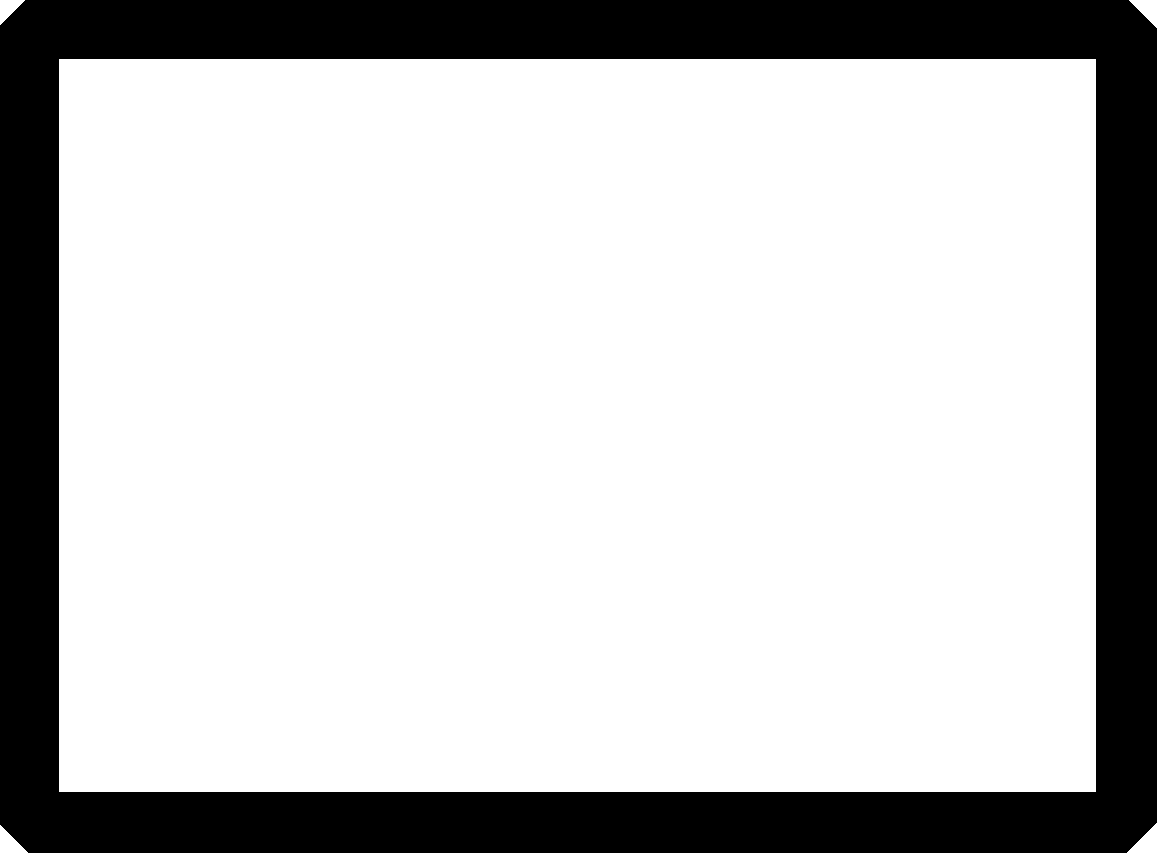 | 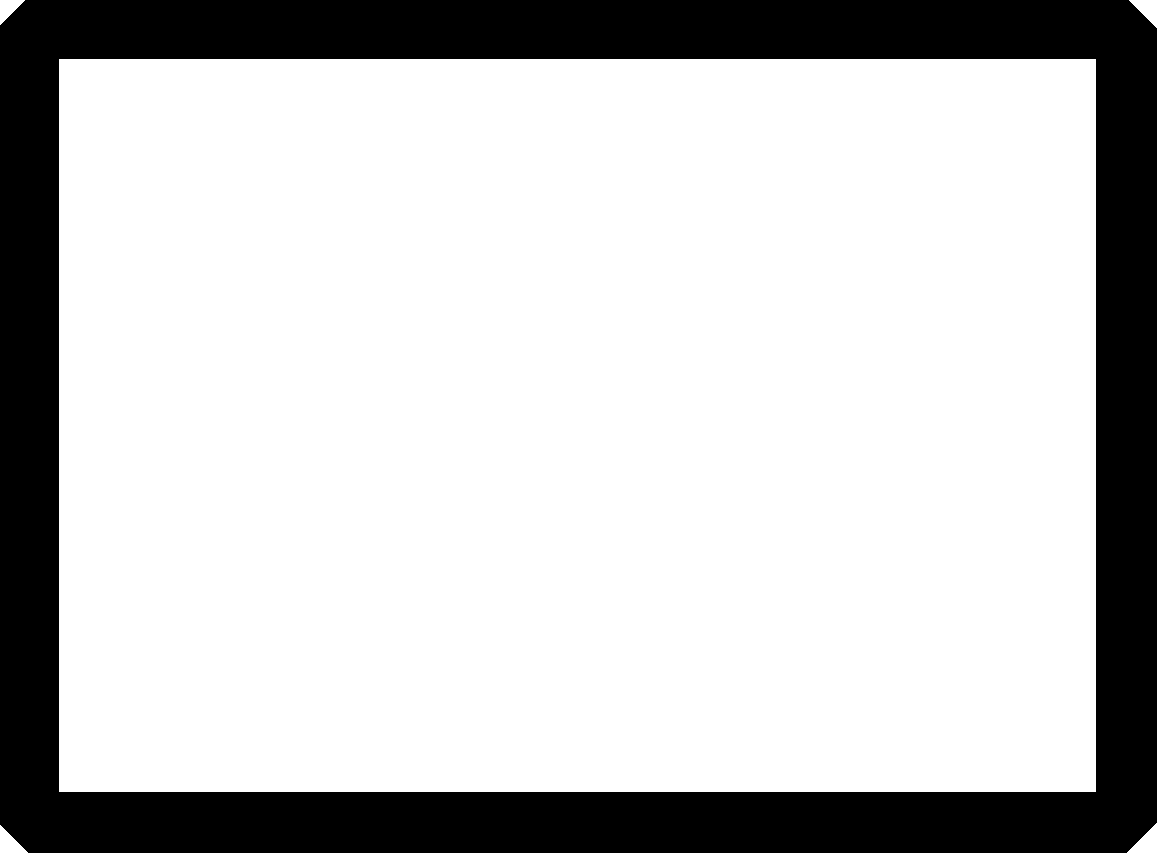 | 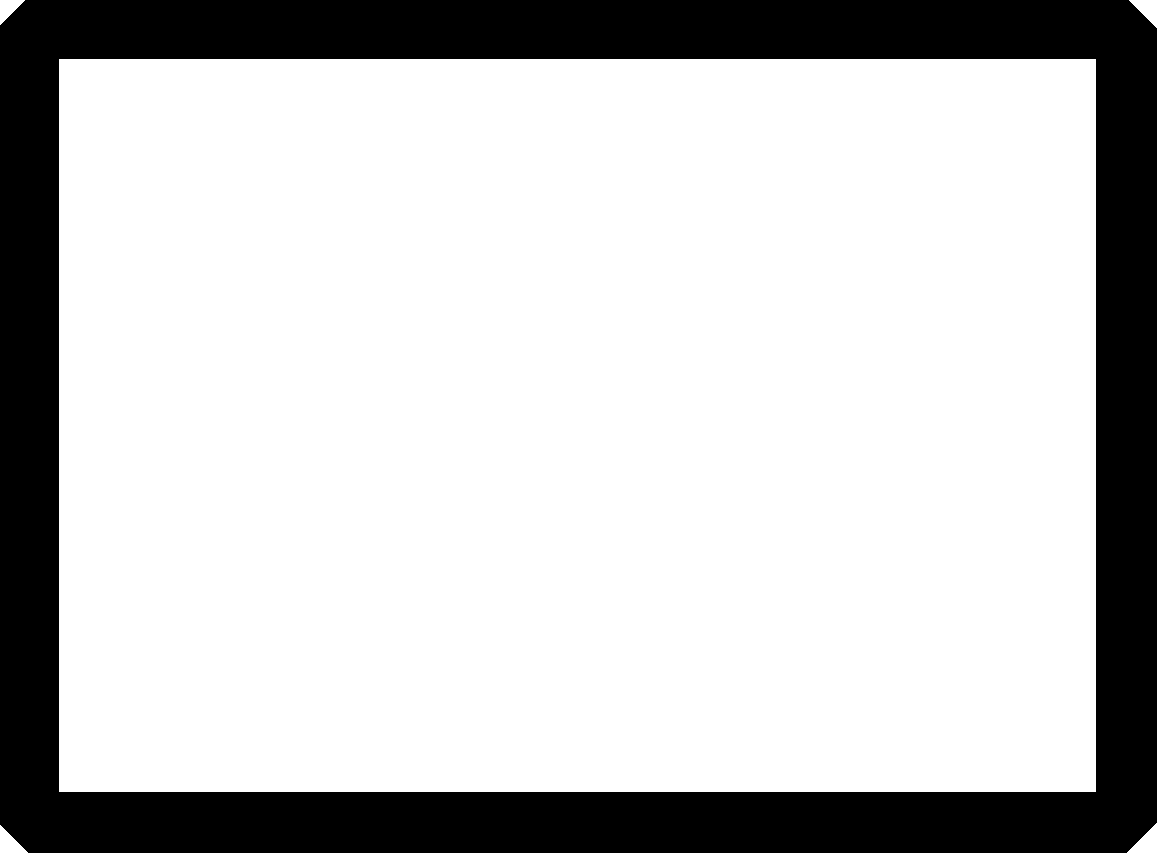 | 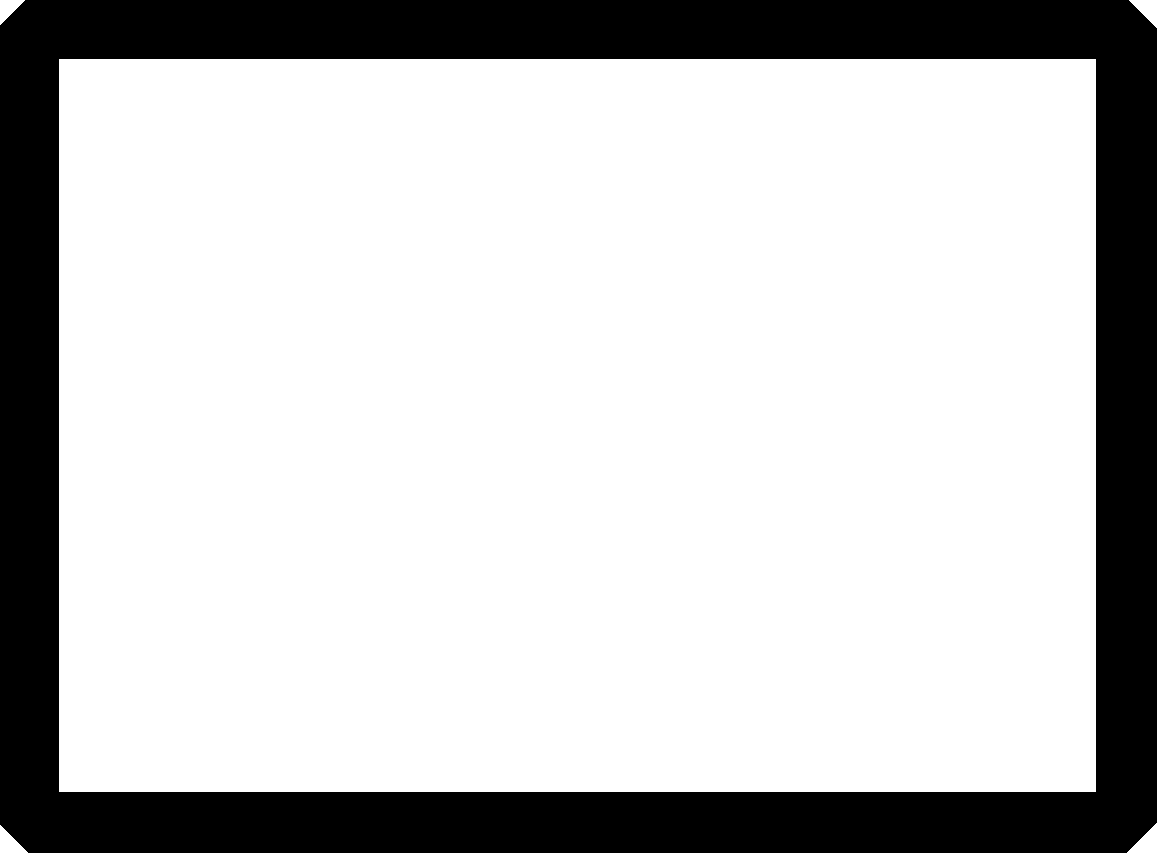 | 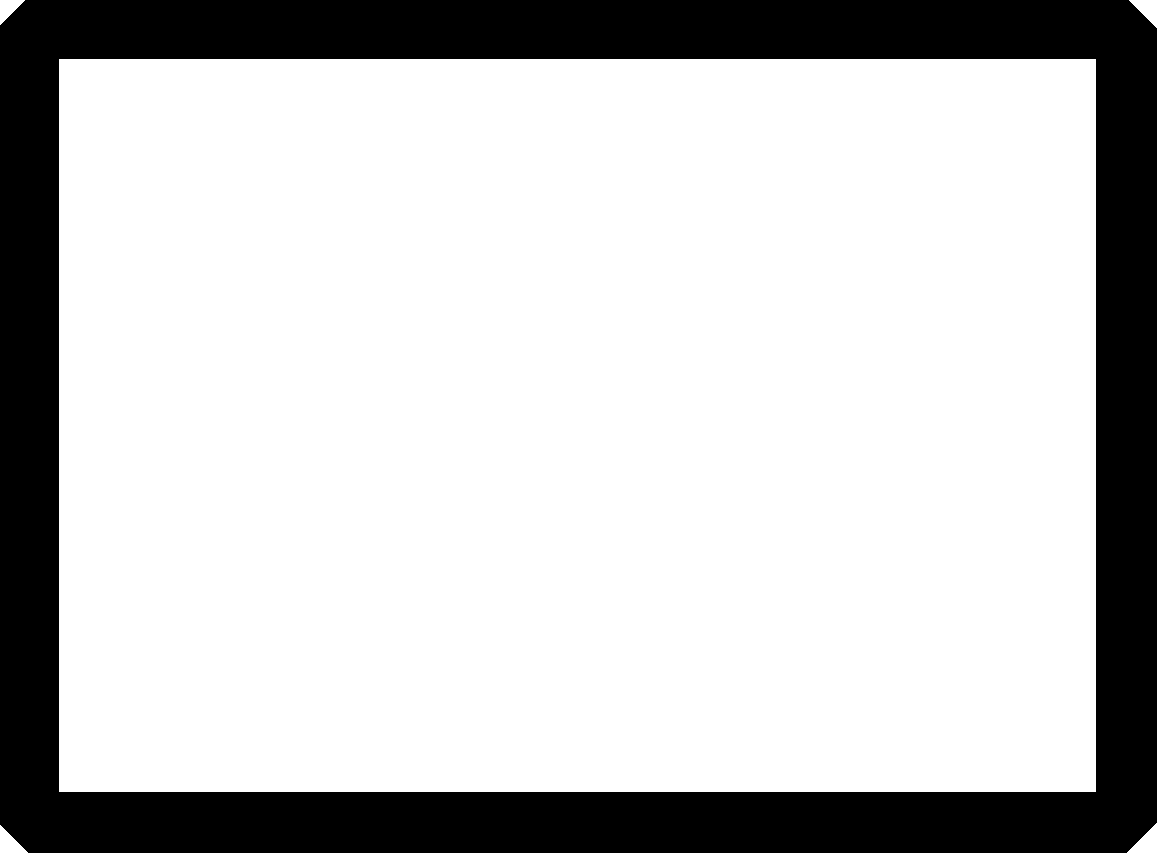 | 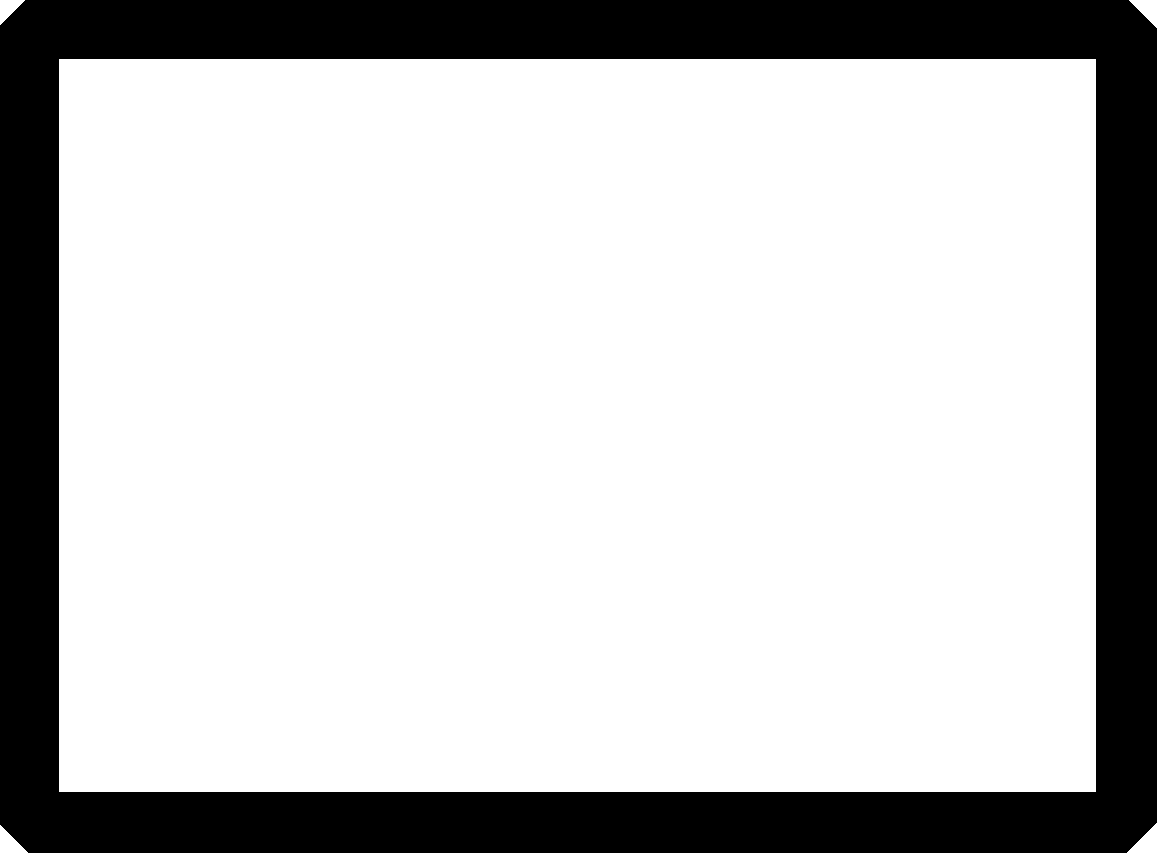 |
| **B2b.** Share files and content with simple tools. | 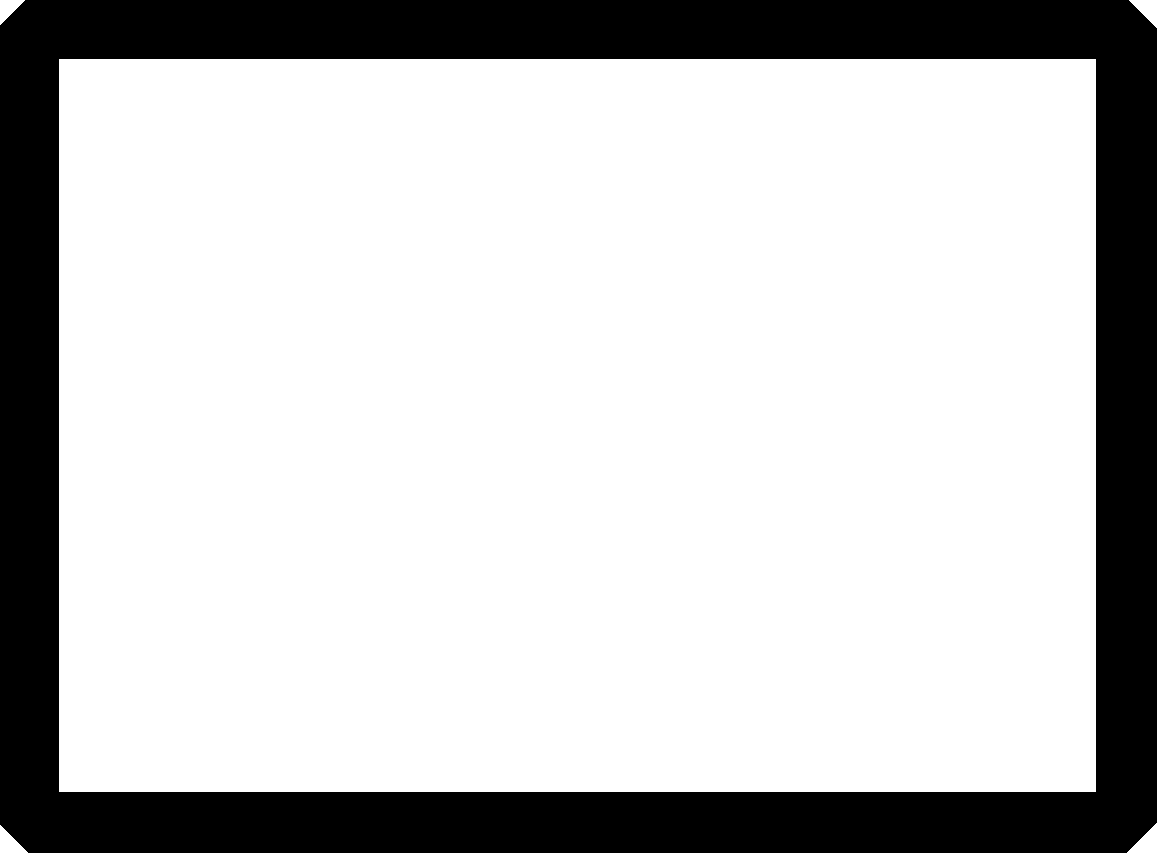 | 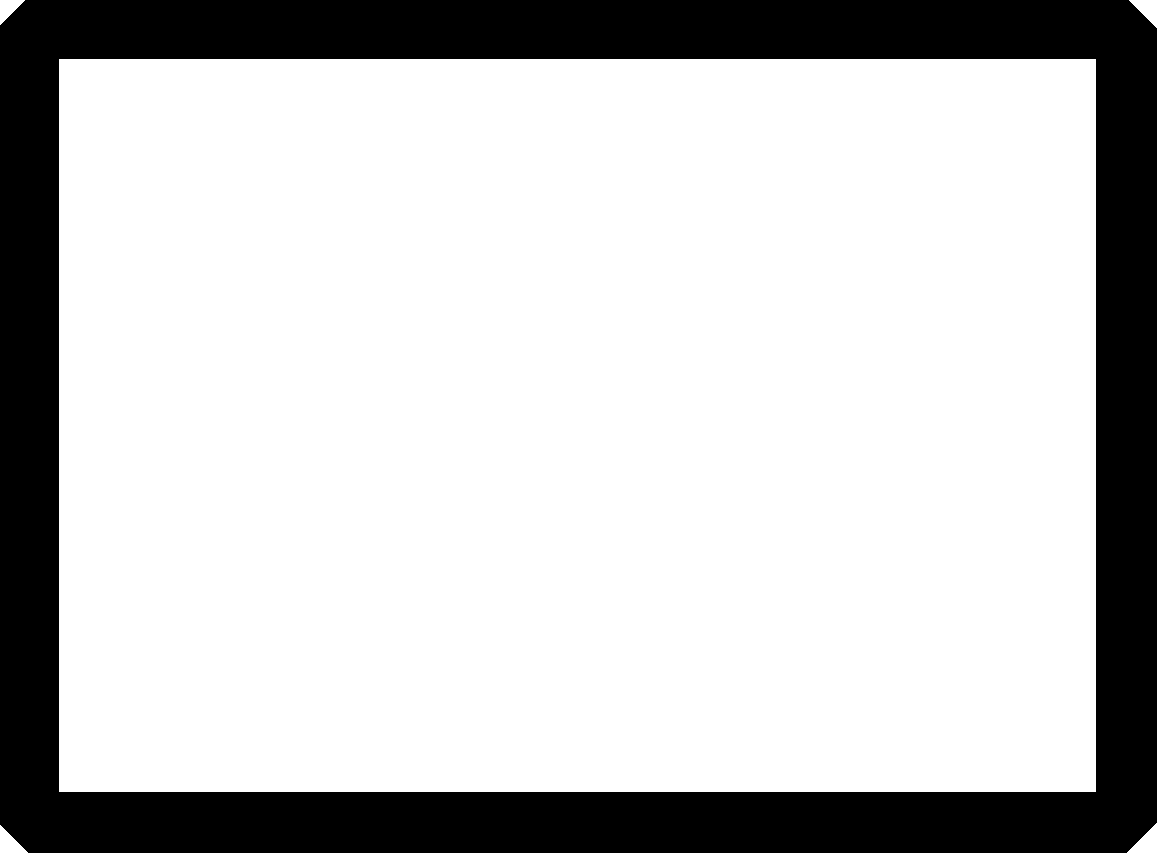 | 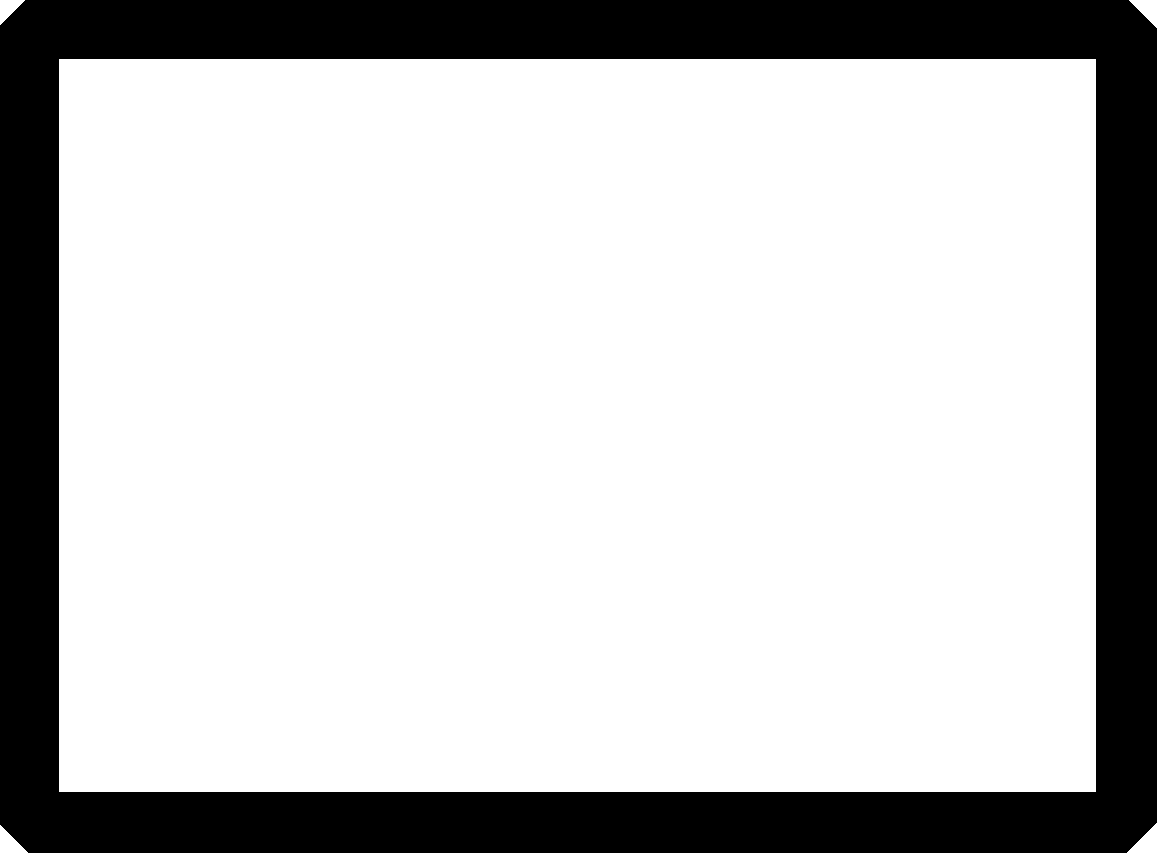 | 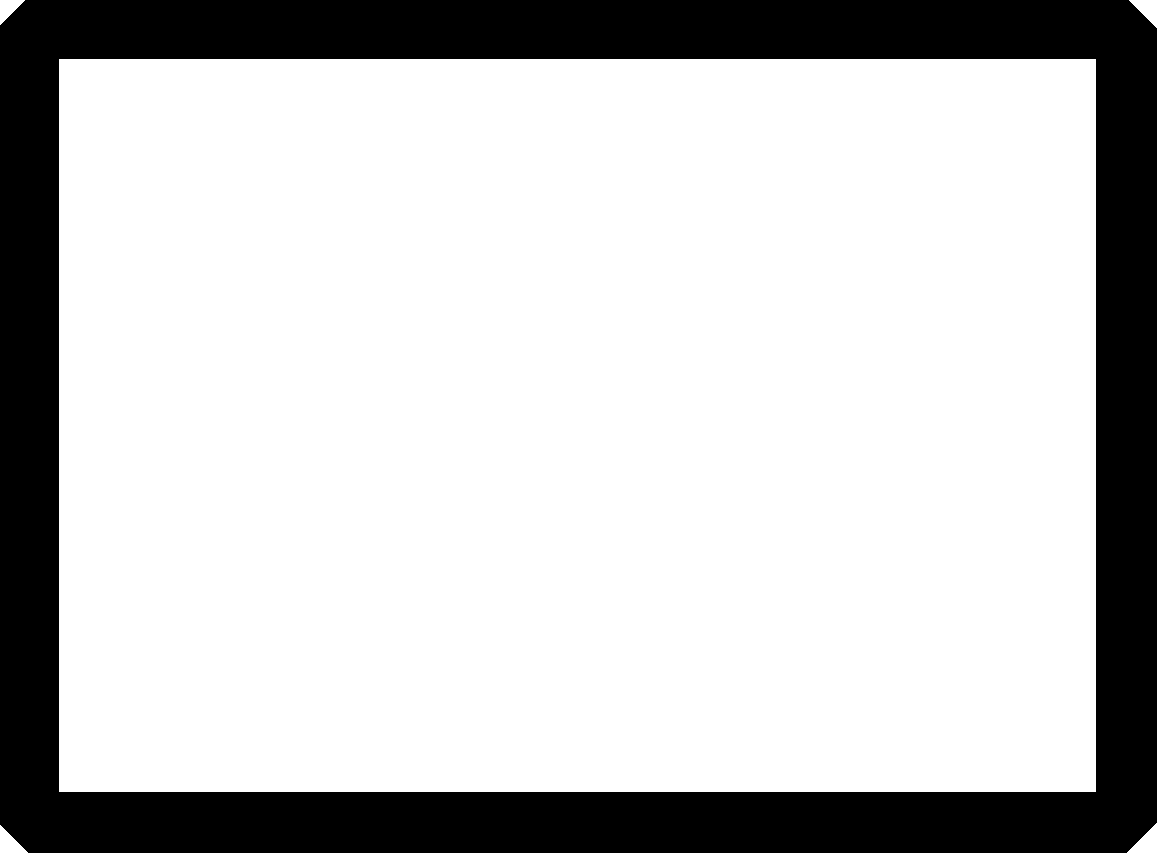 | 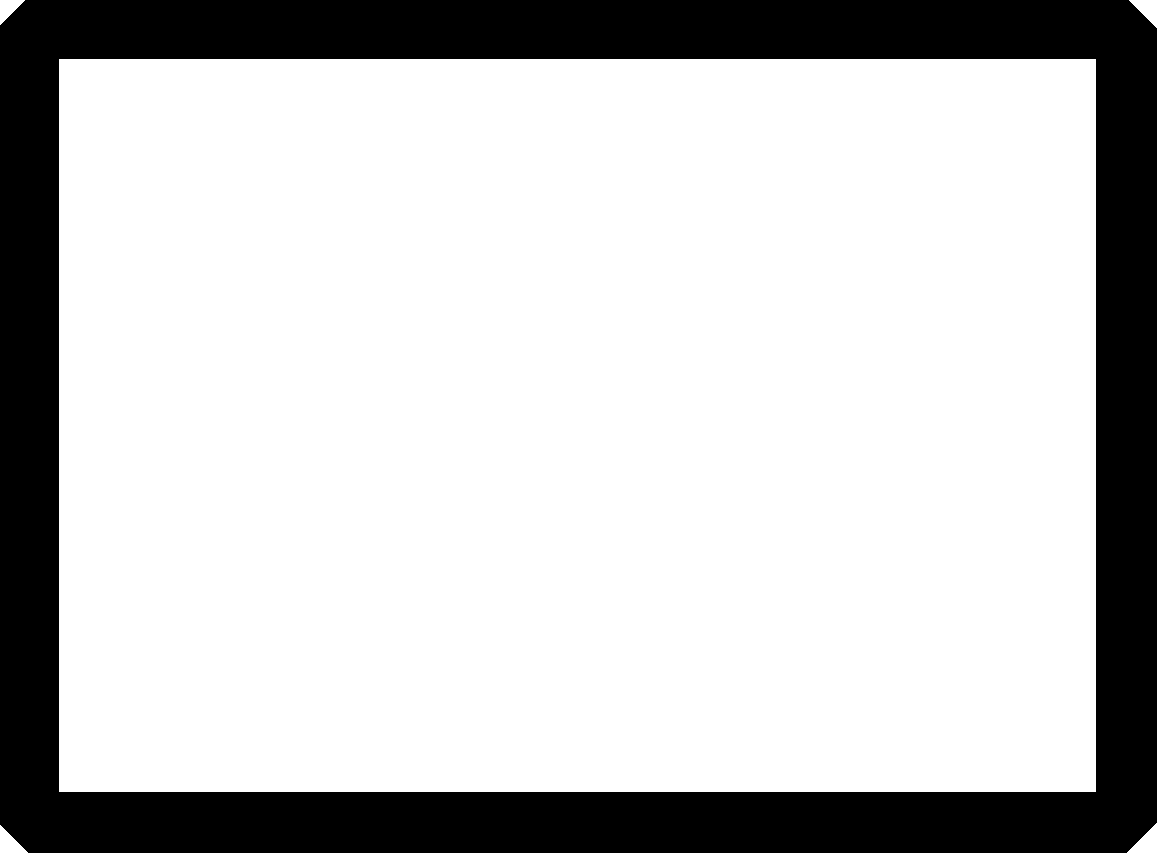 | 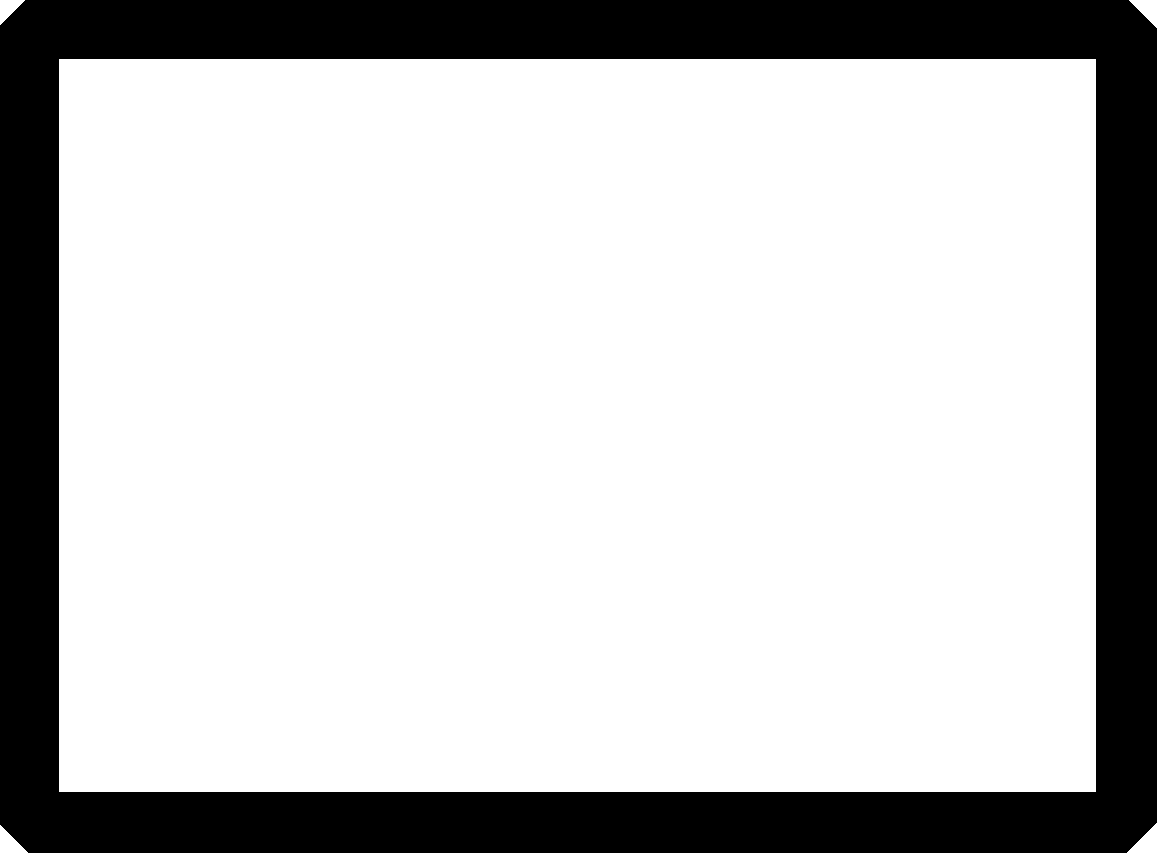 | 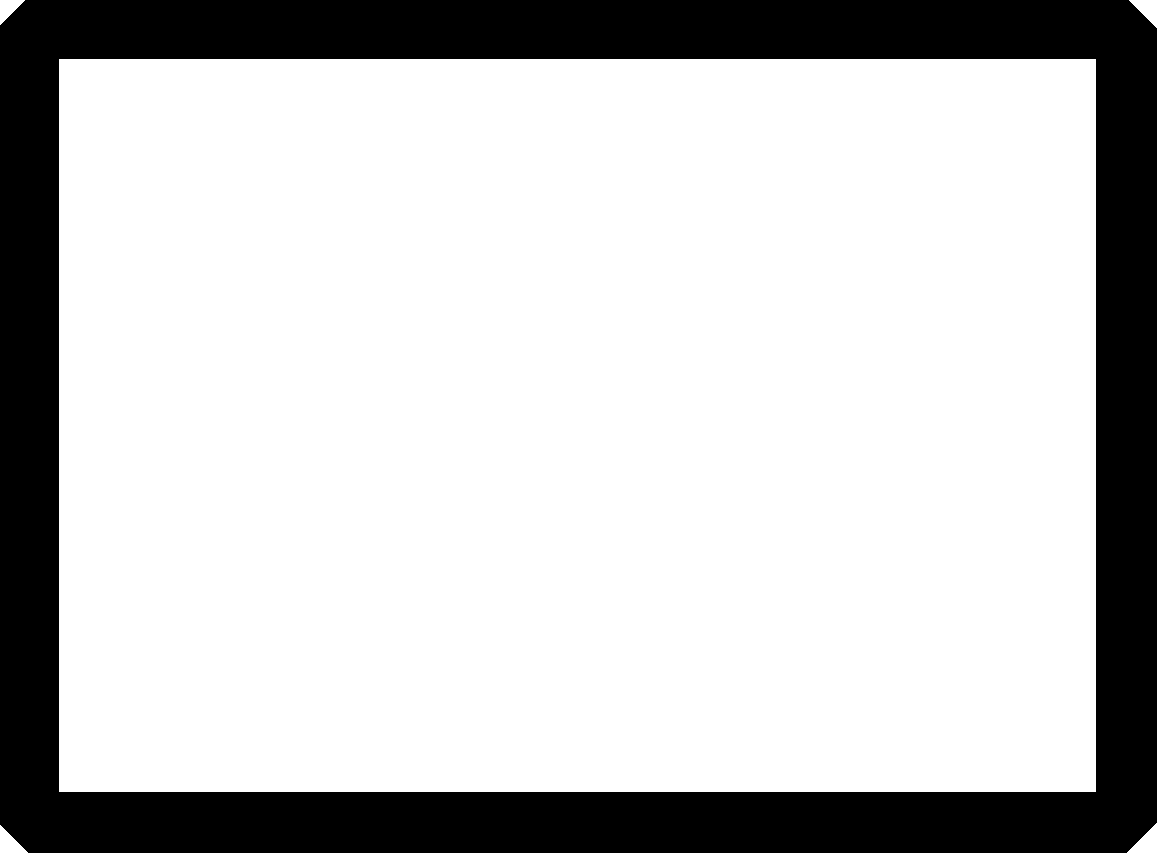 |
| **B2c.** Use digital technologies to interact with services (e.g., governments, banks, hospitals). | 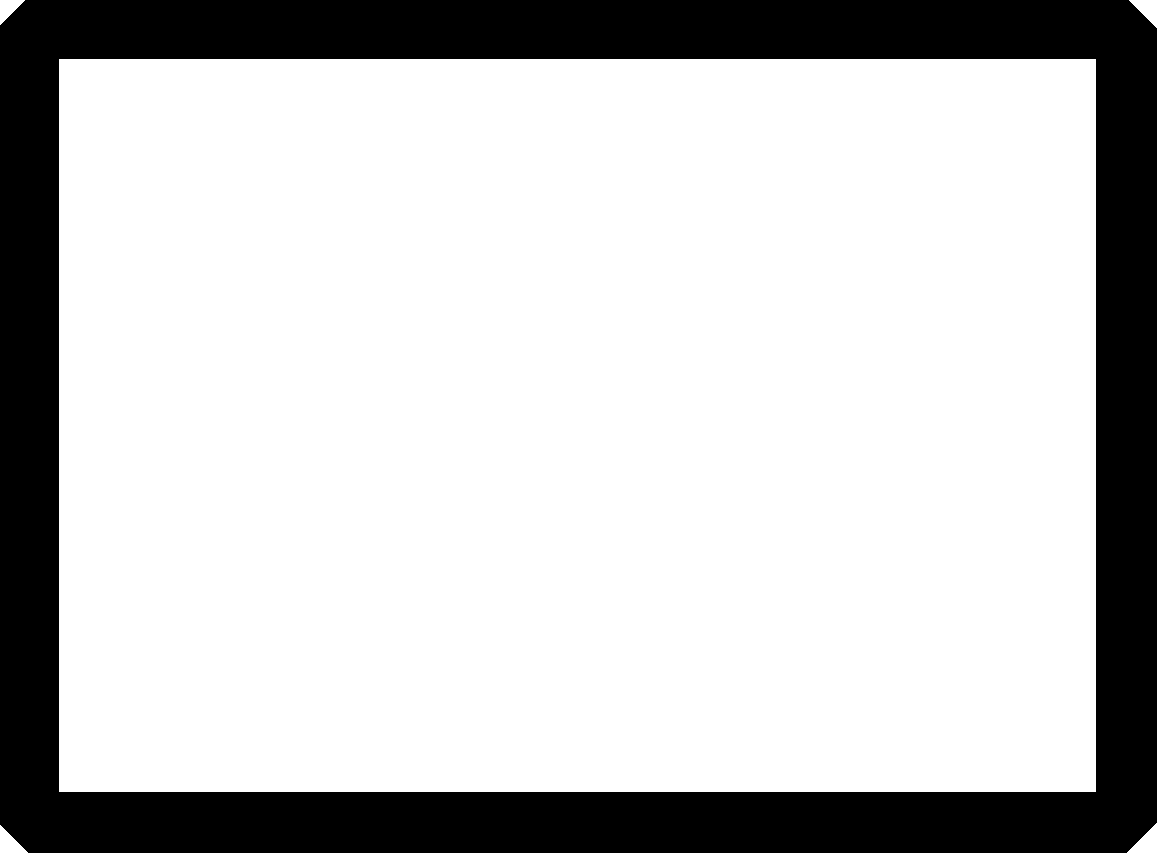 | 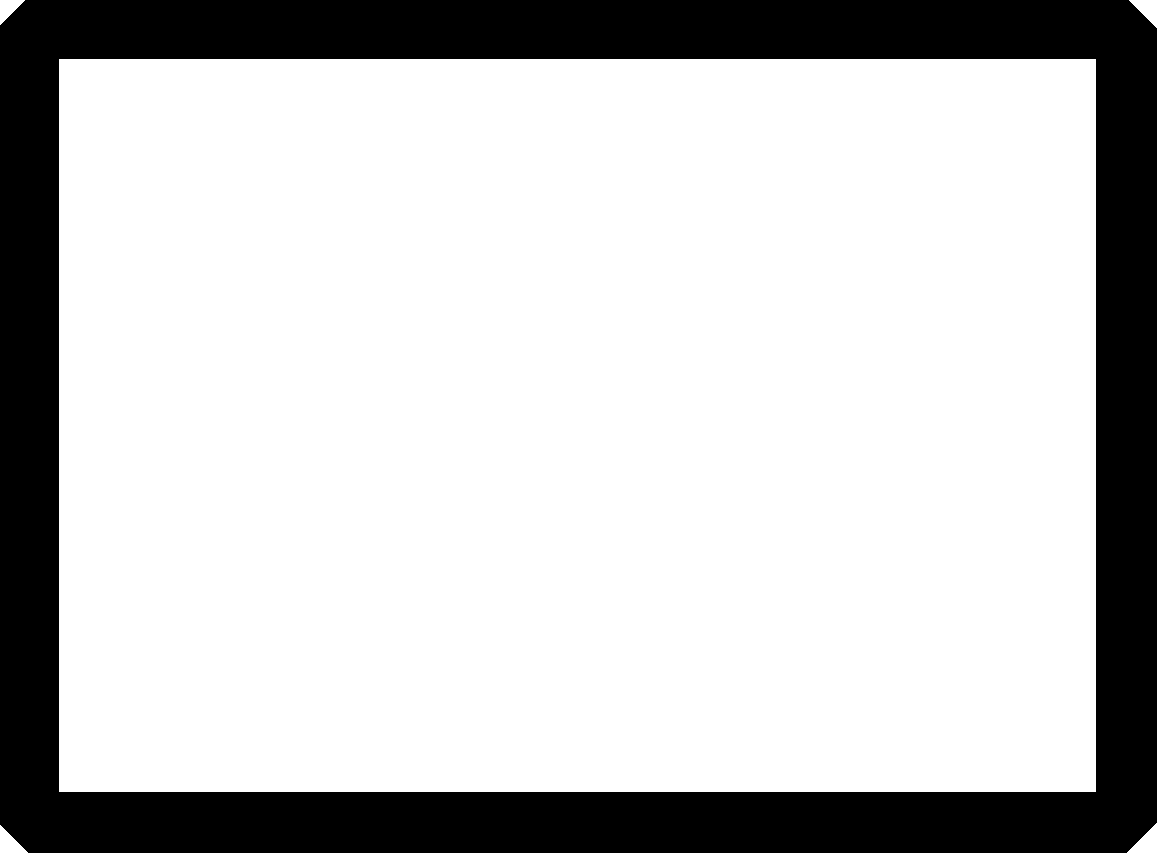 | 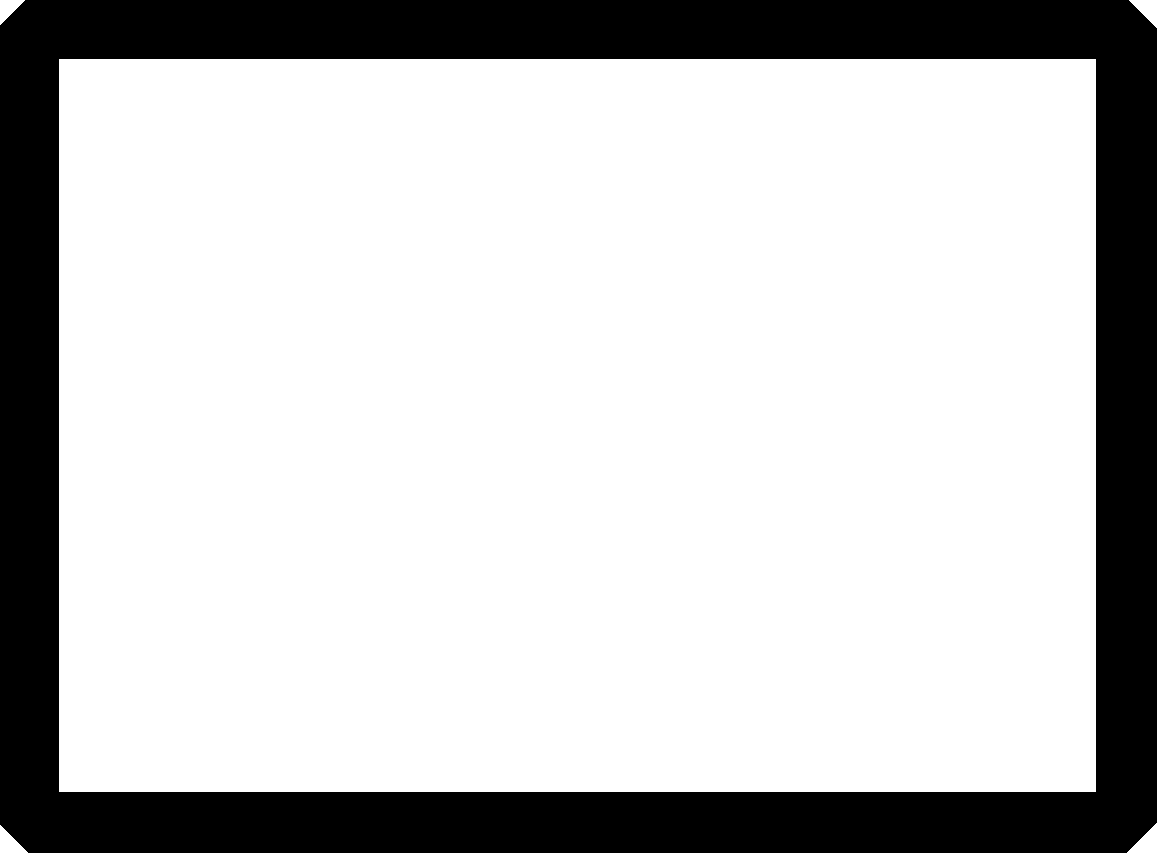 | 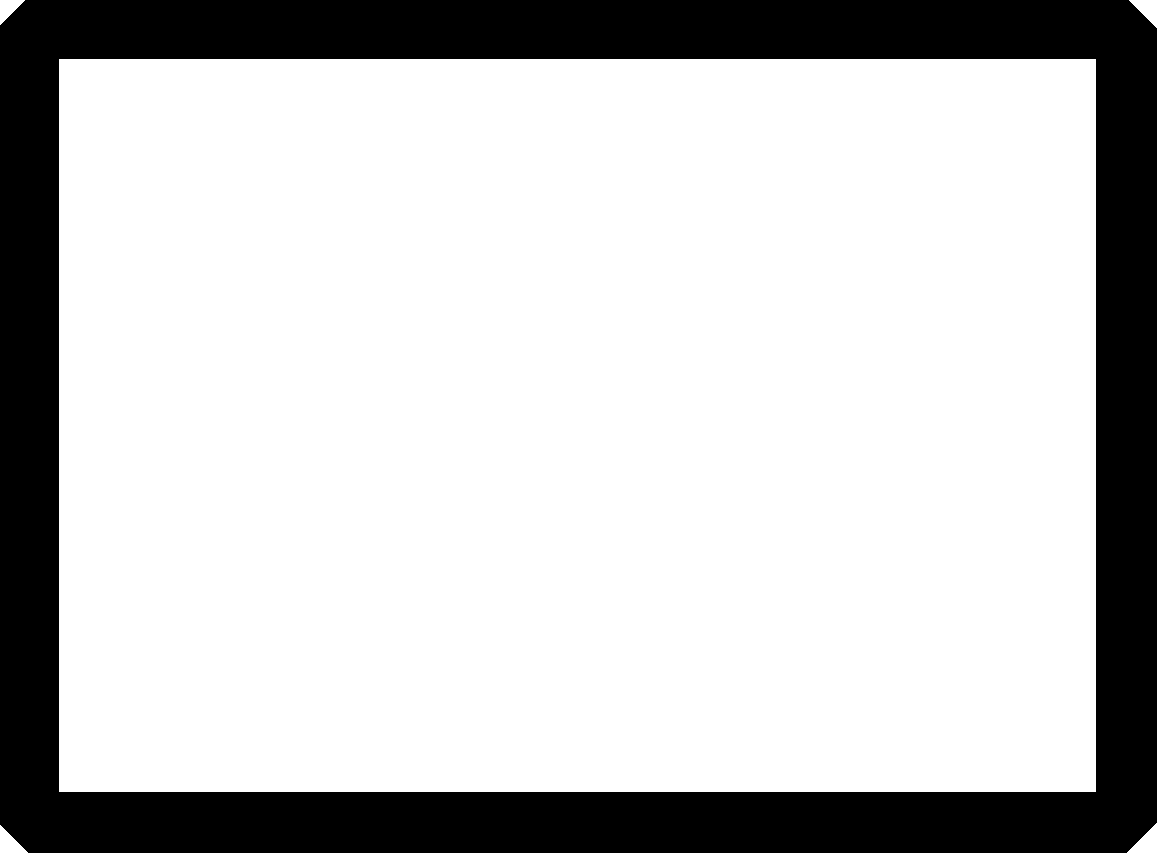 | 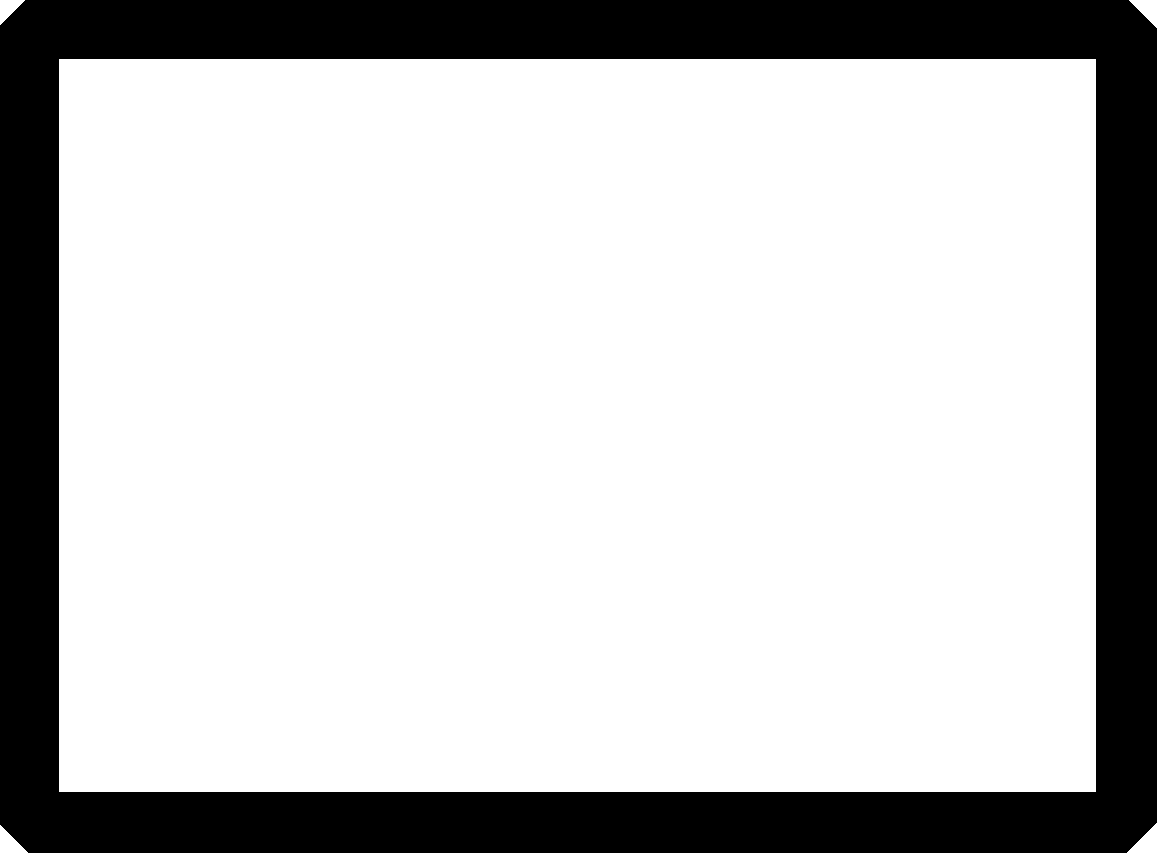 | 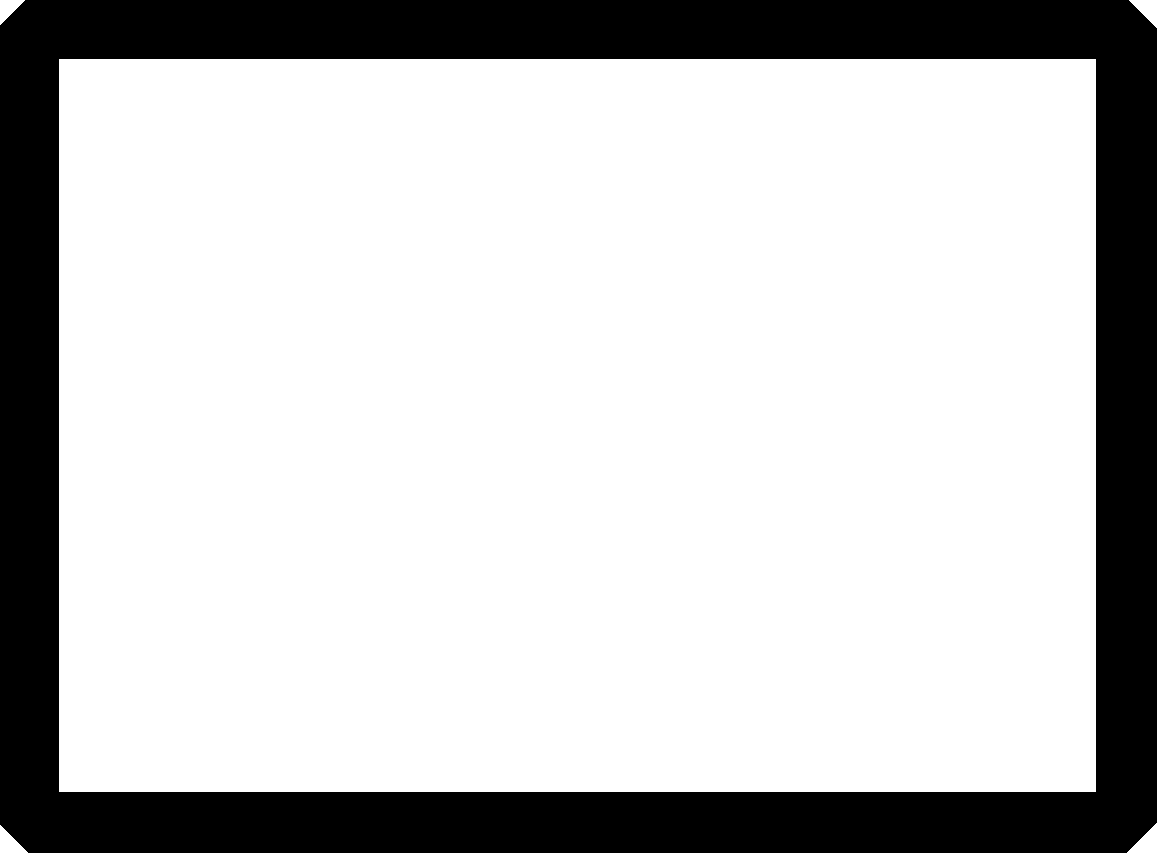 | 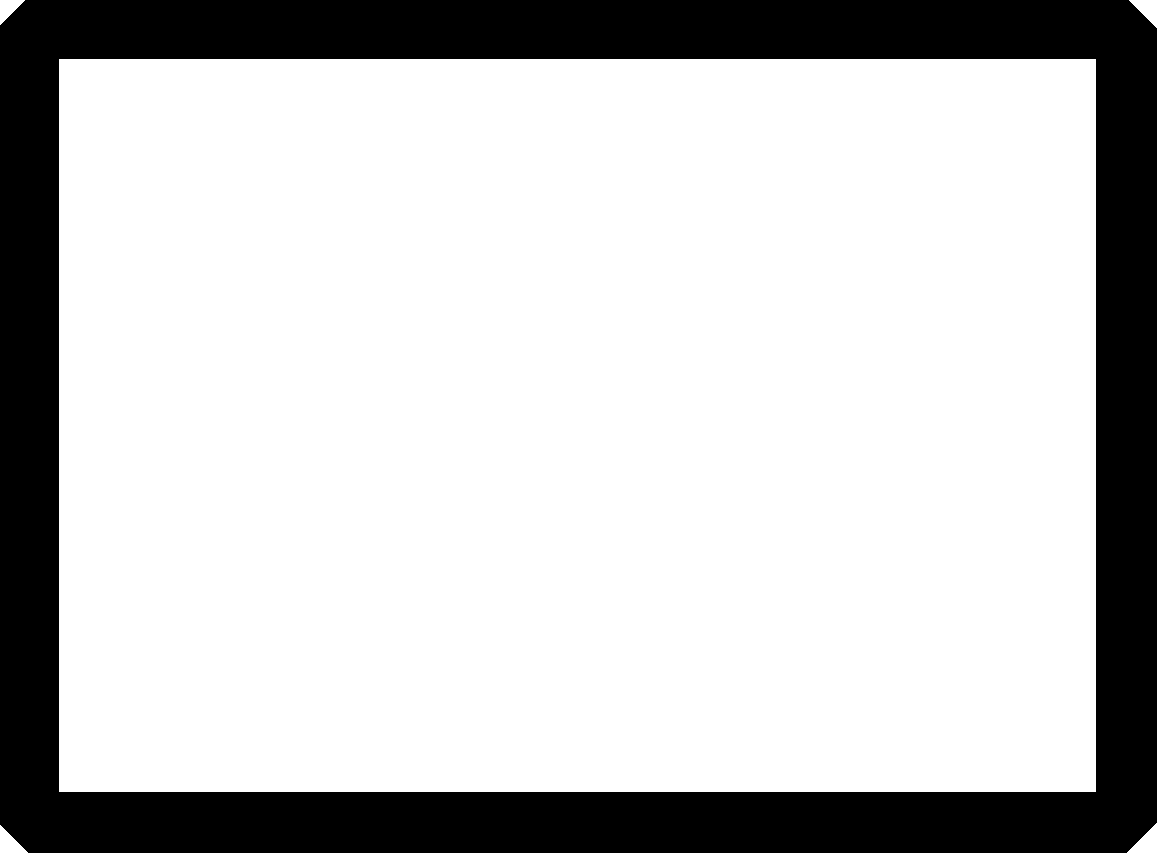 |
| **B2d.** Use social networking and online collaboration tools. | 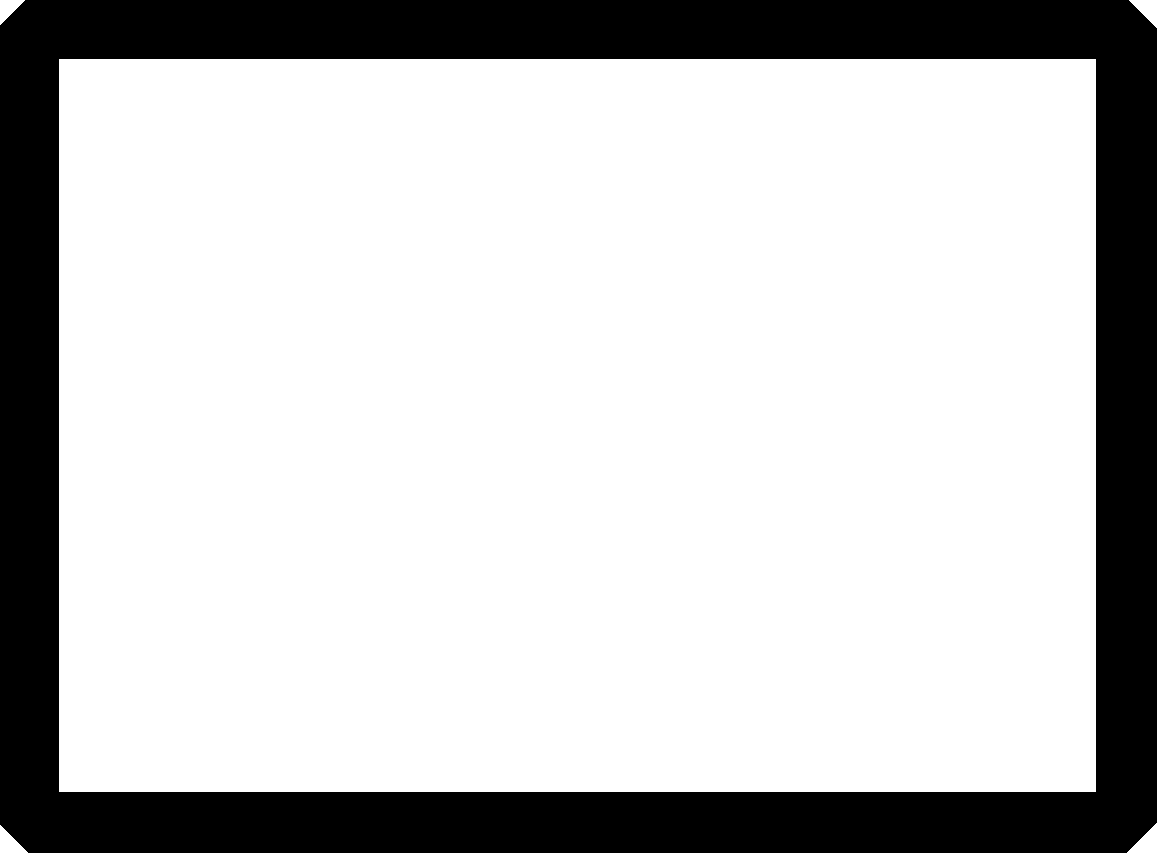 | 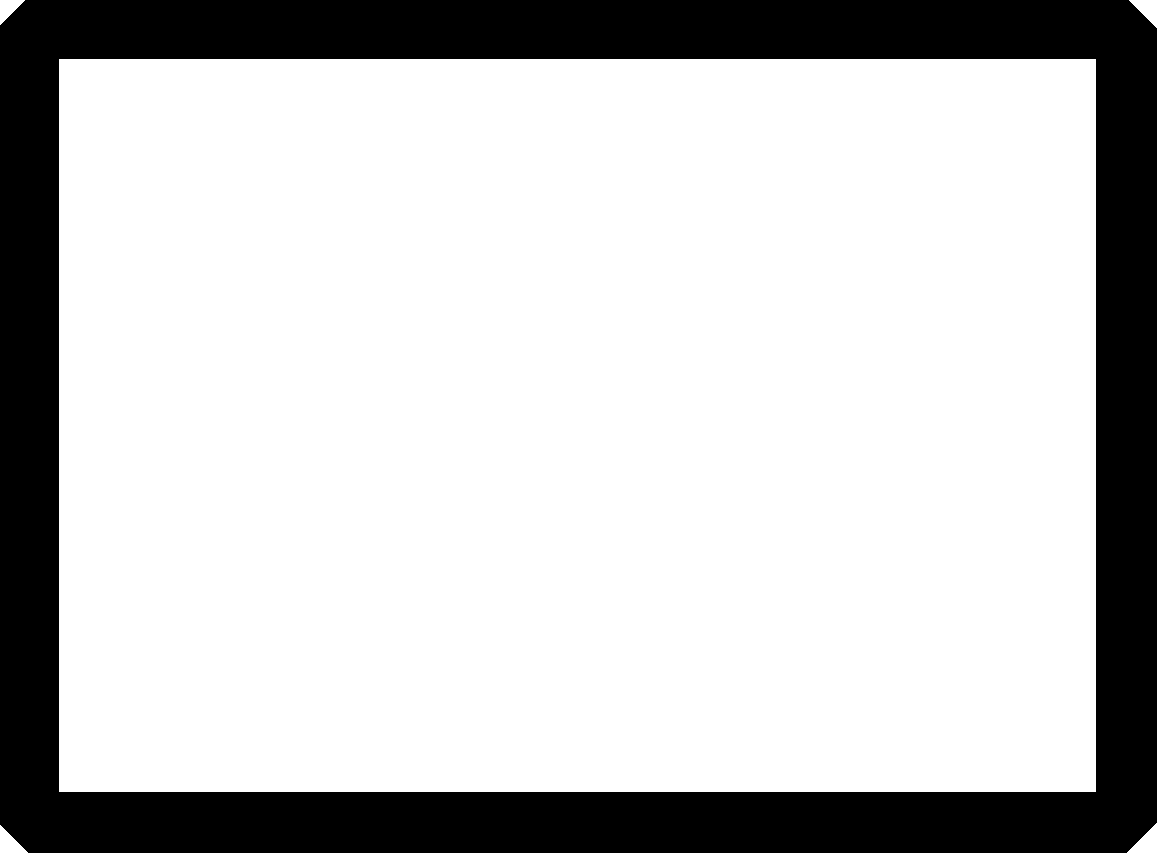 | 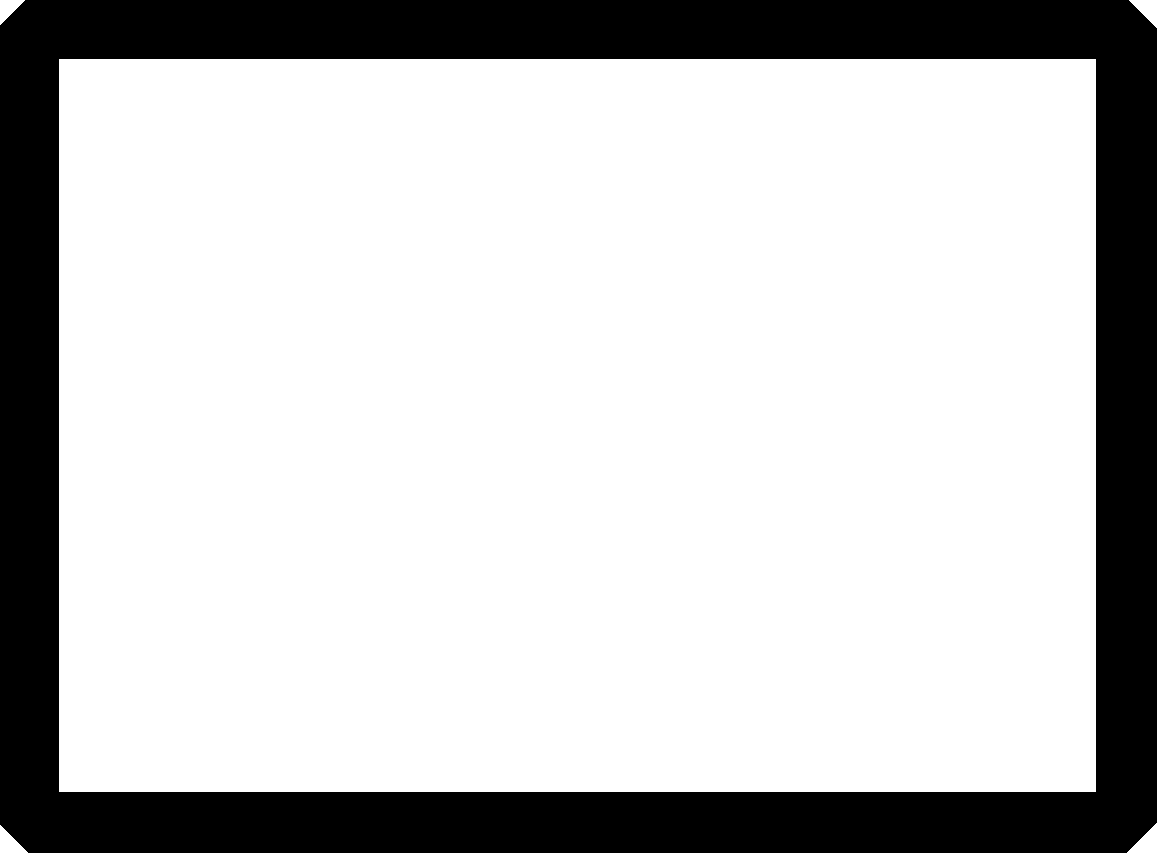 | 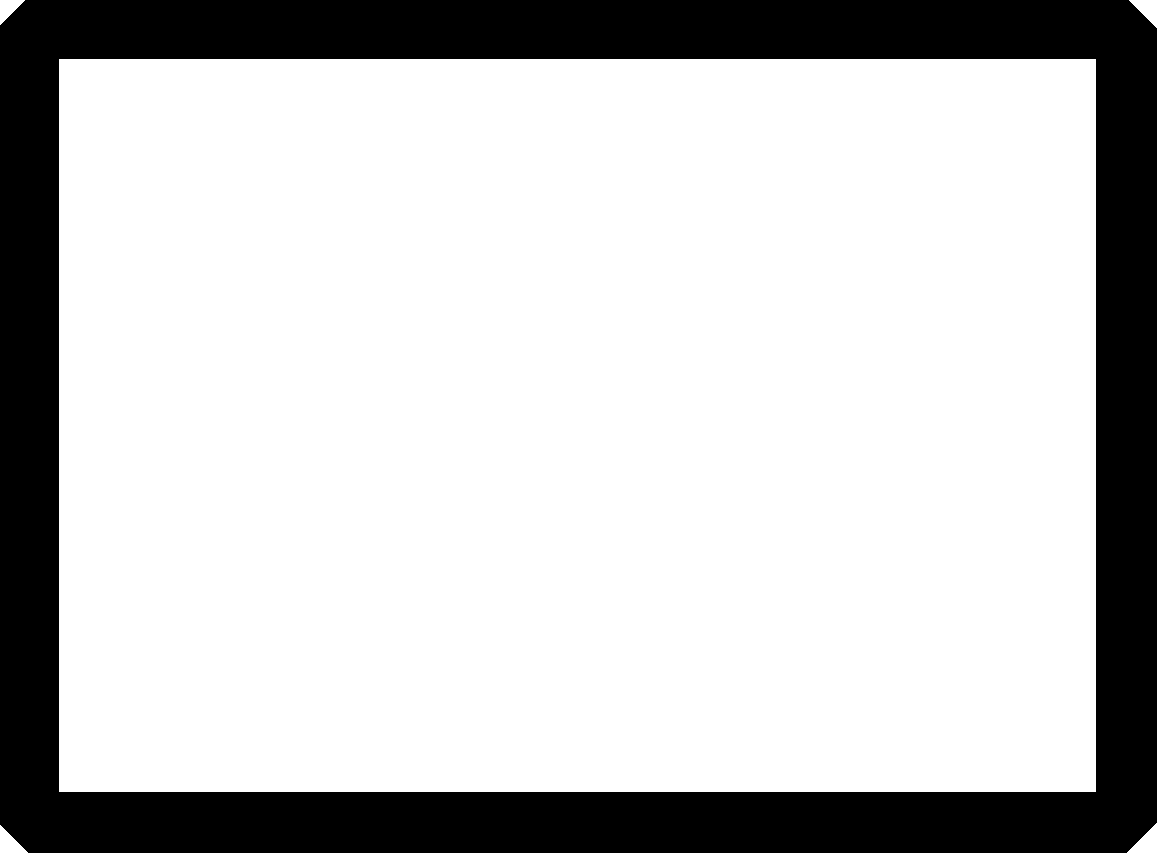 | 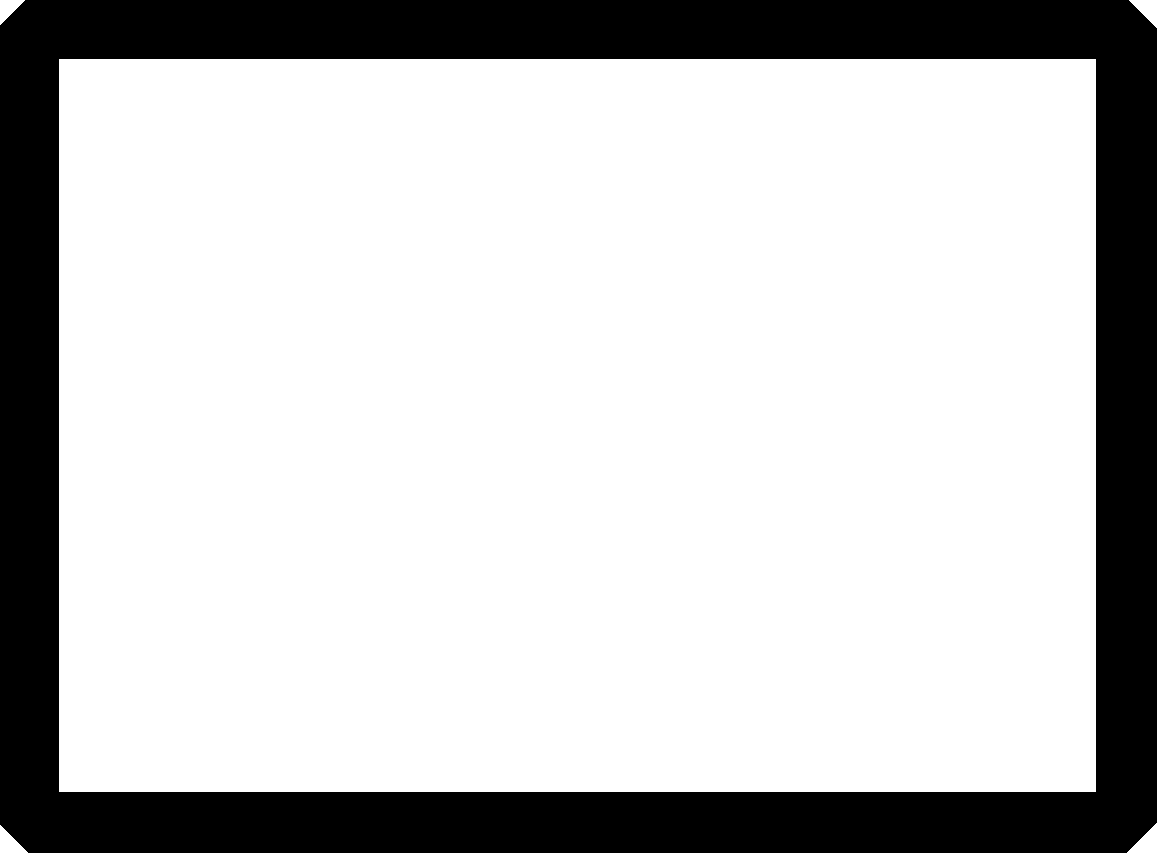 | 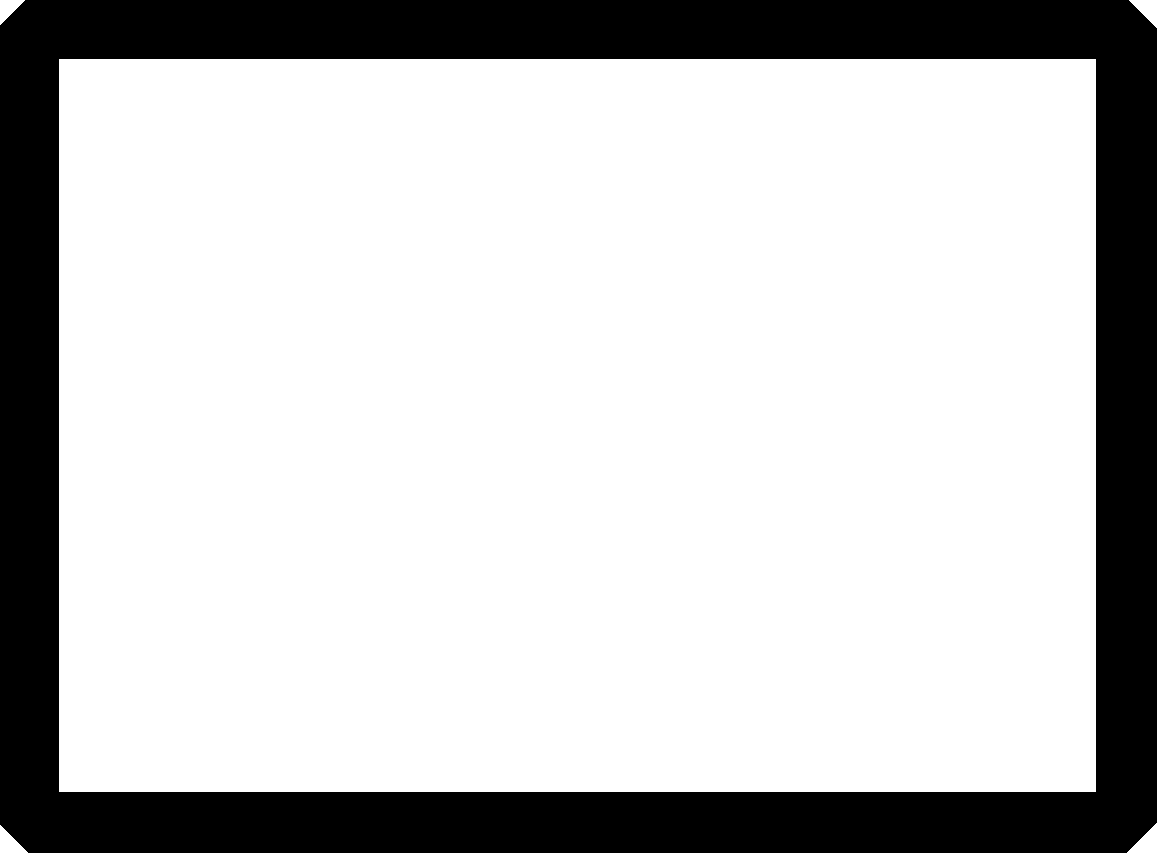 | 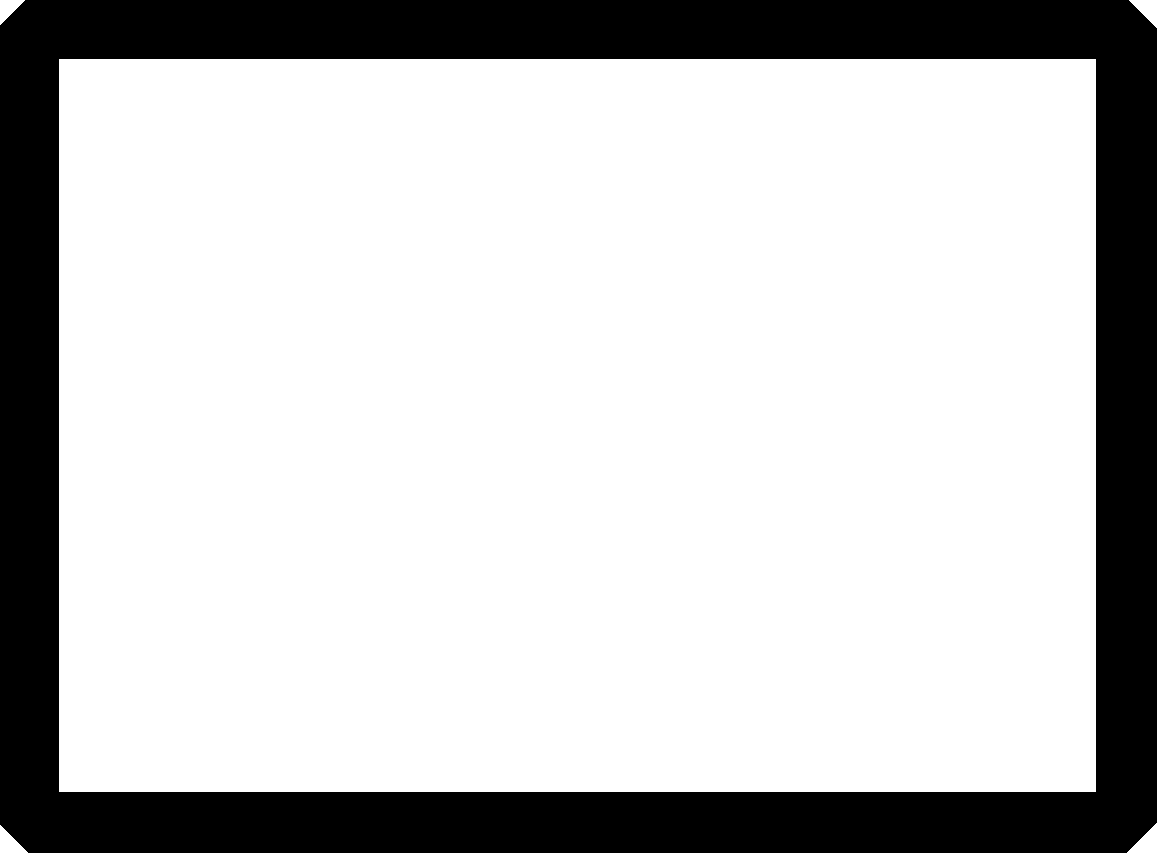 |
| **B2e.** Use digital communication standards (e.g., liking, retweeting, sharing a story, sending a DM). | 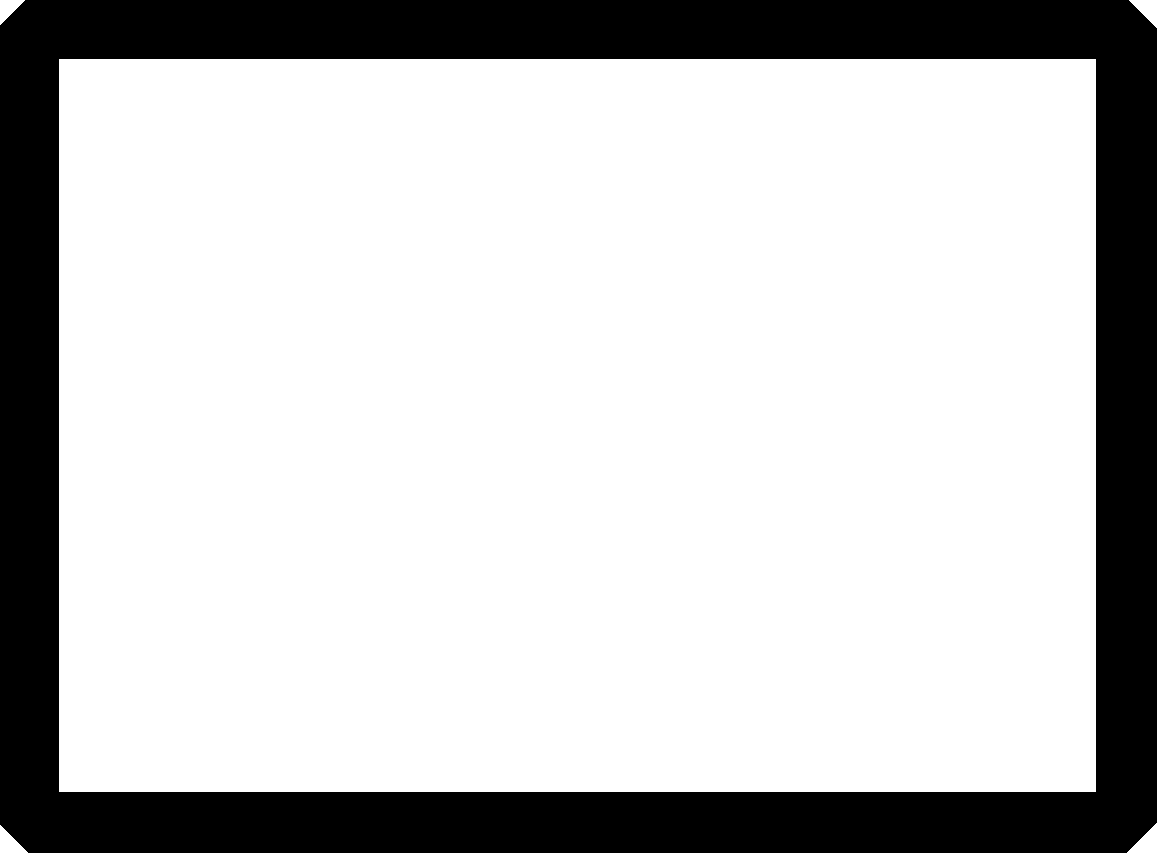 | 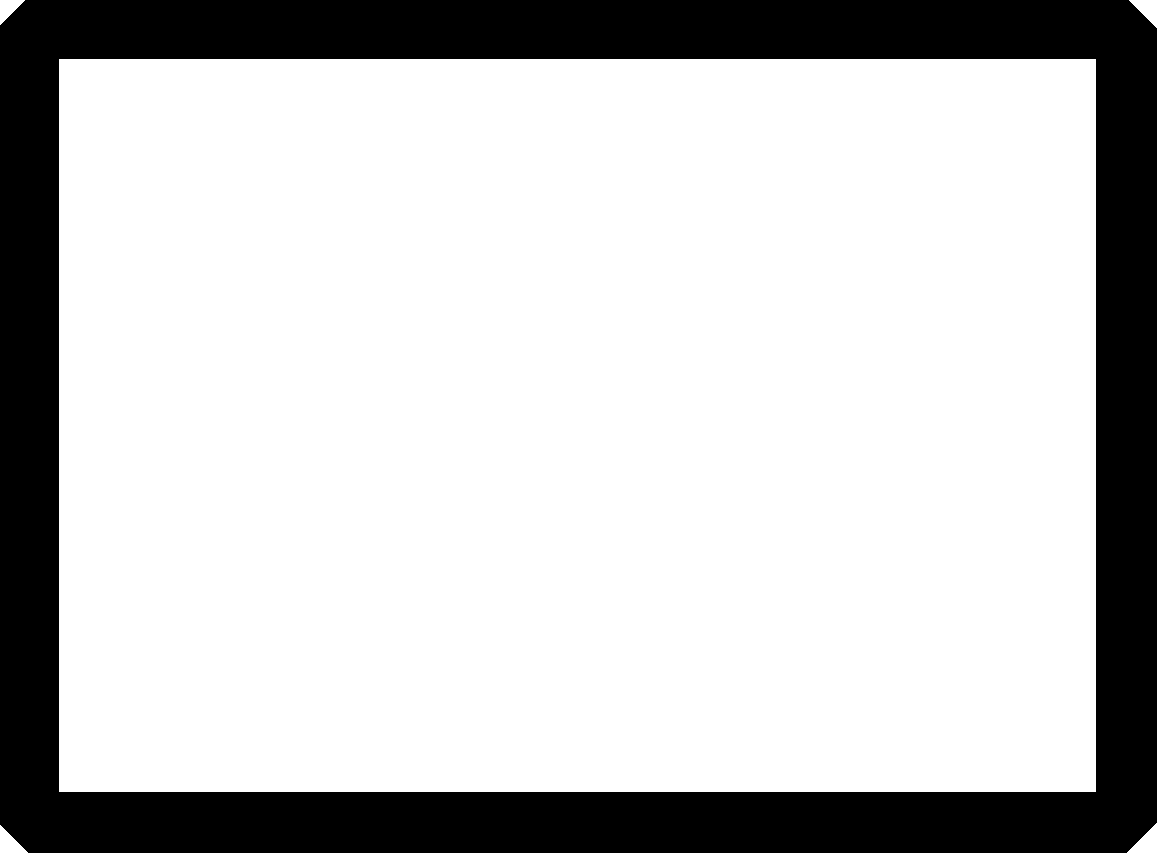 | 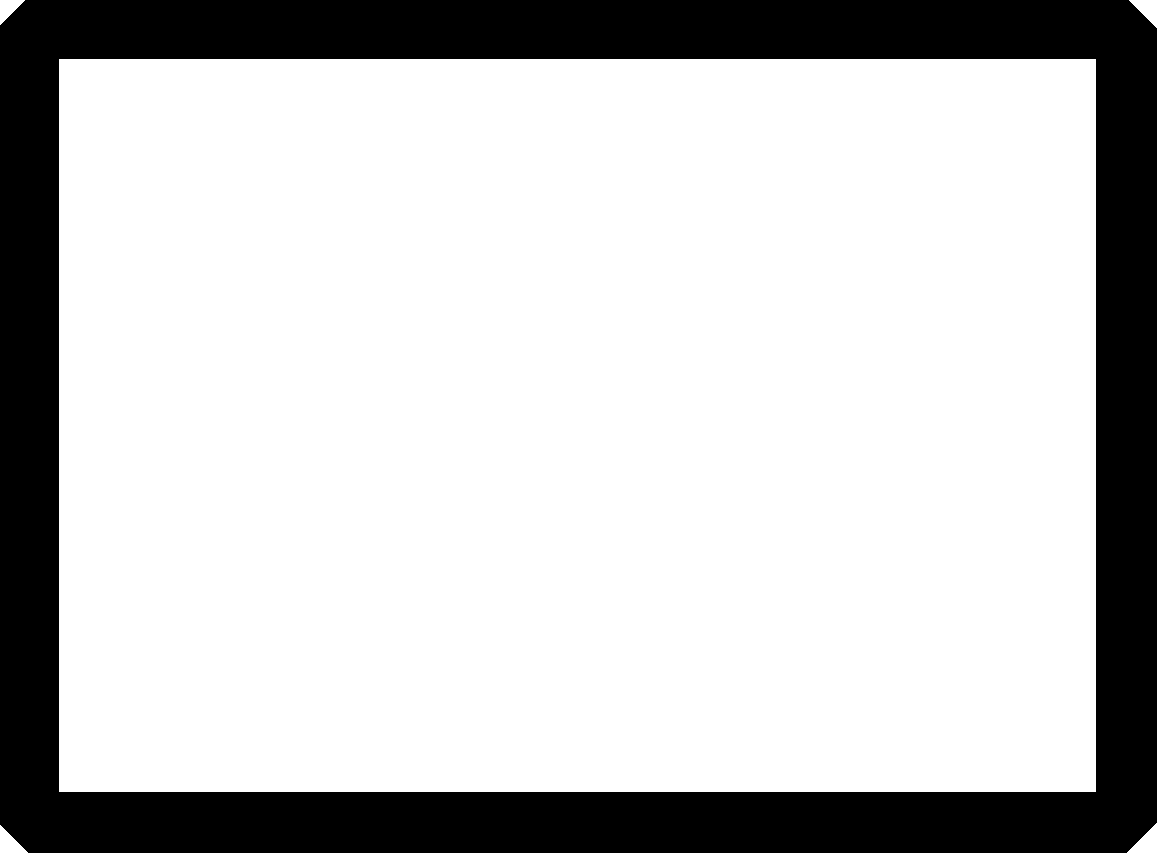 | 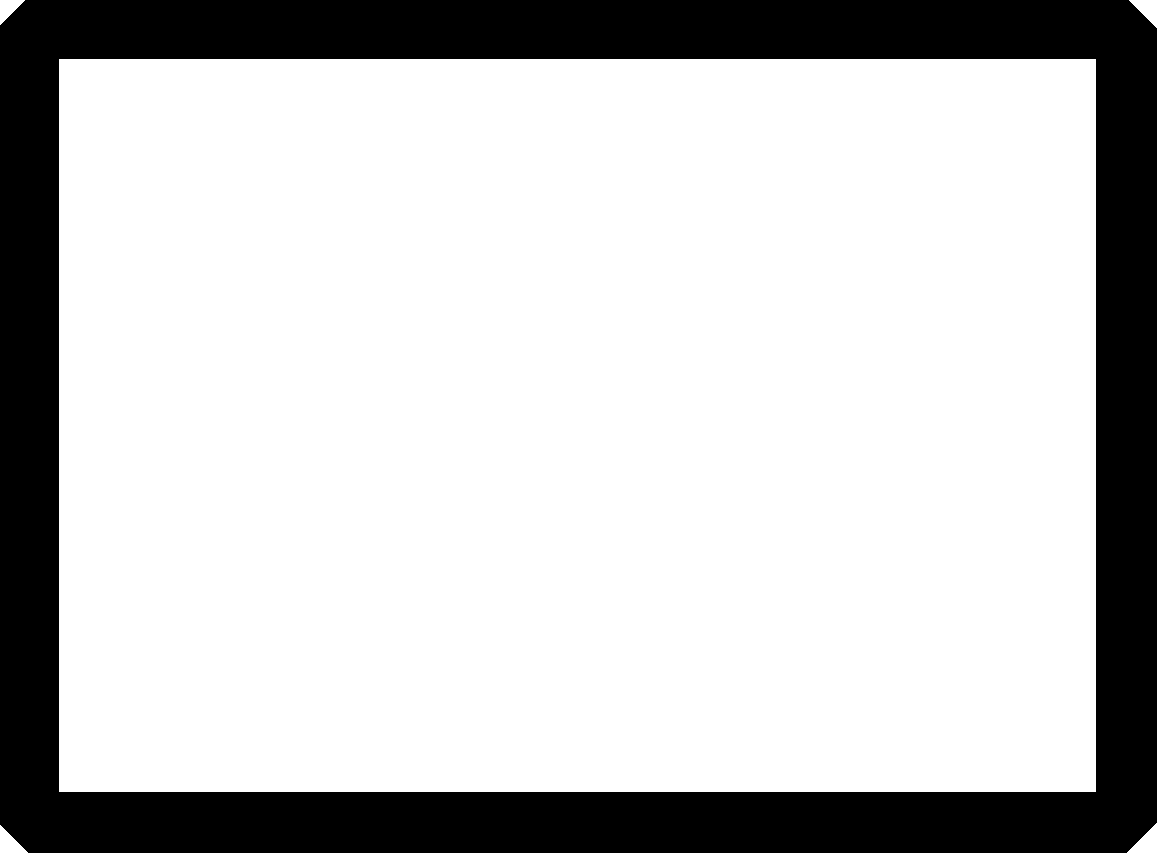 | 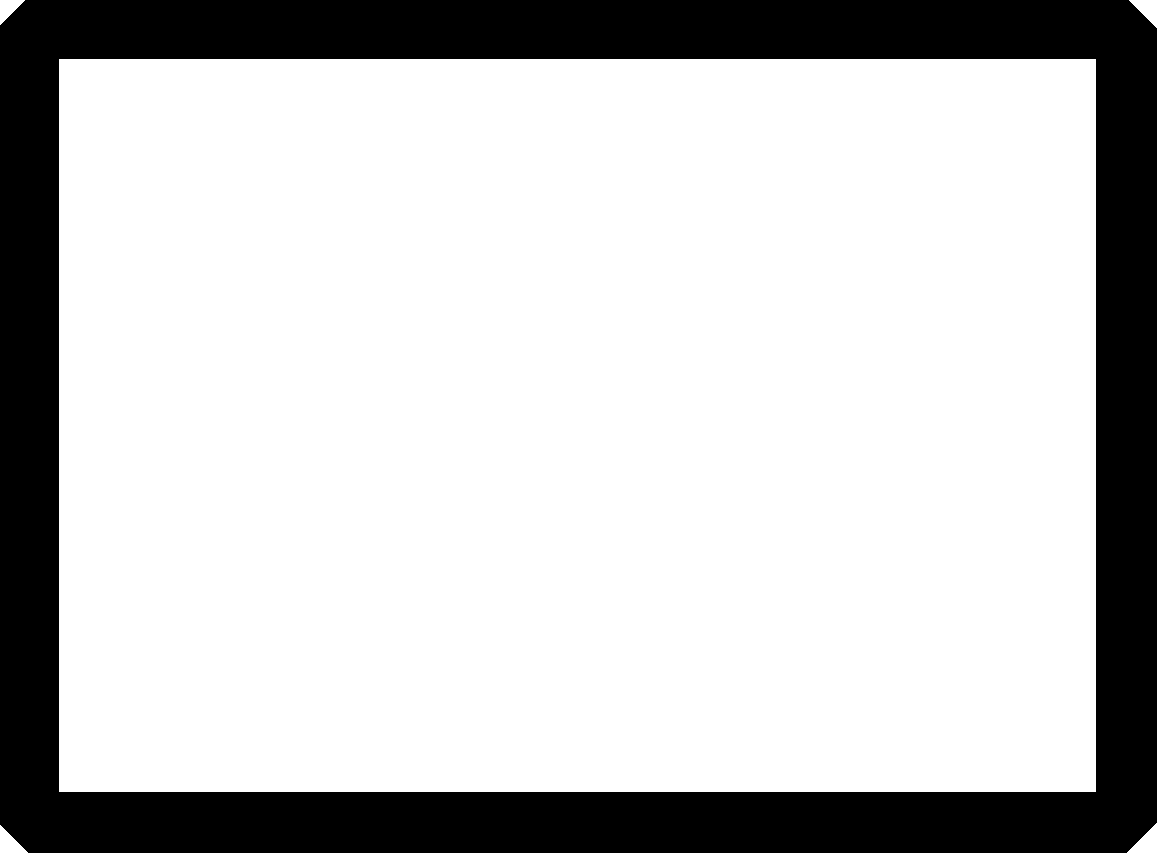 | 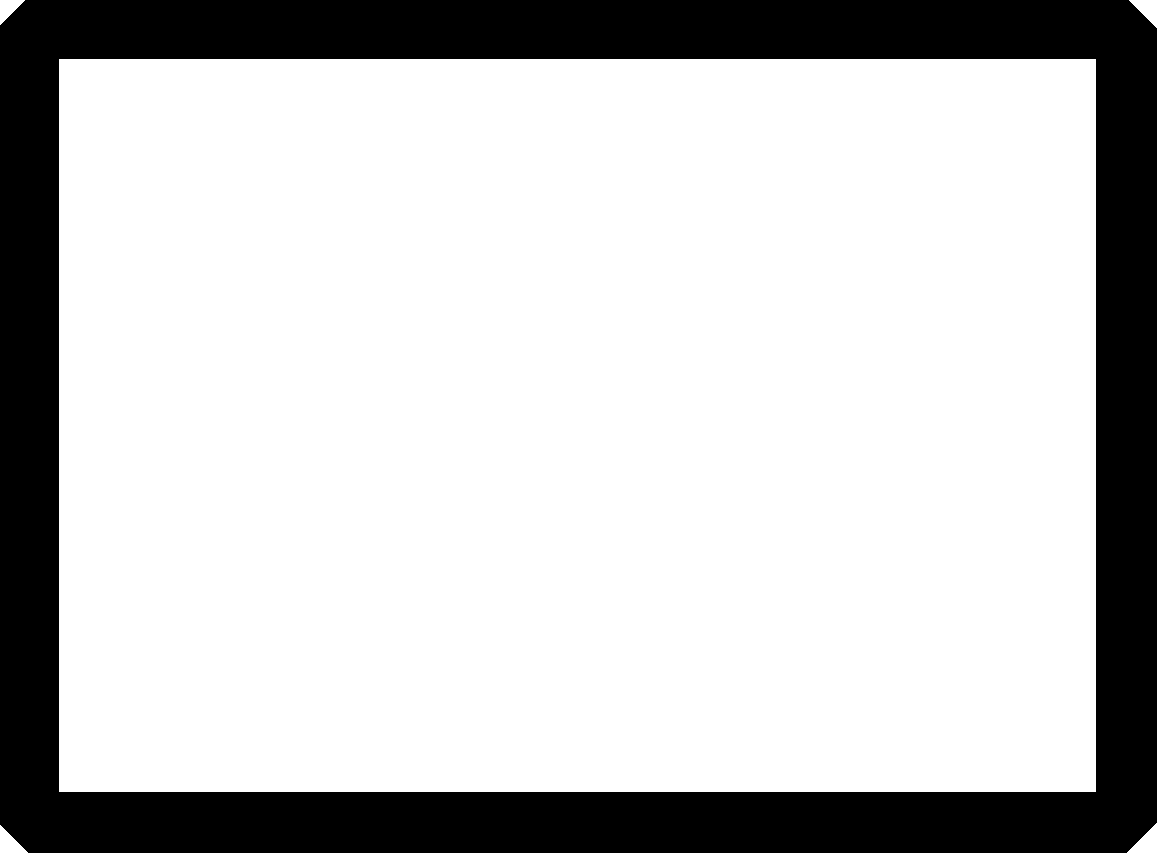 | 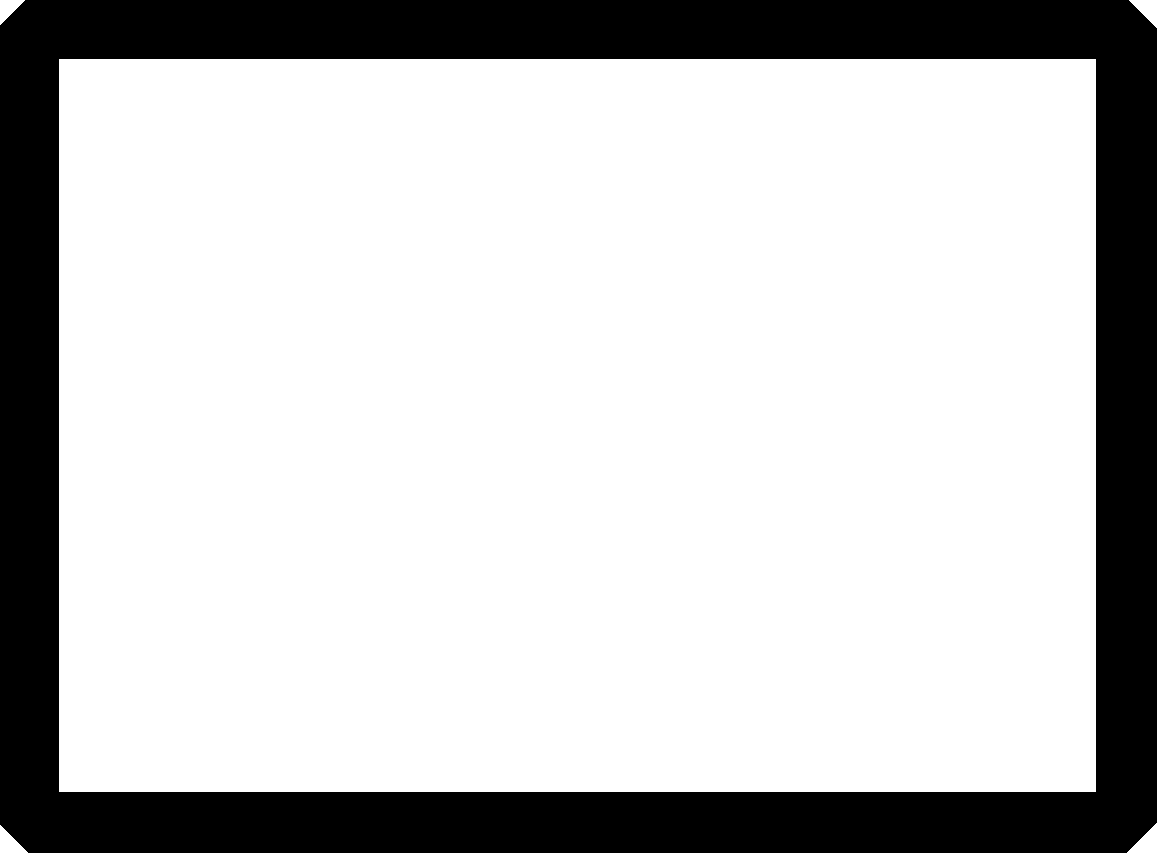 |
| **B2f.** Communicate statistical information according to the characteristics of the audience. | 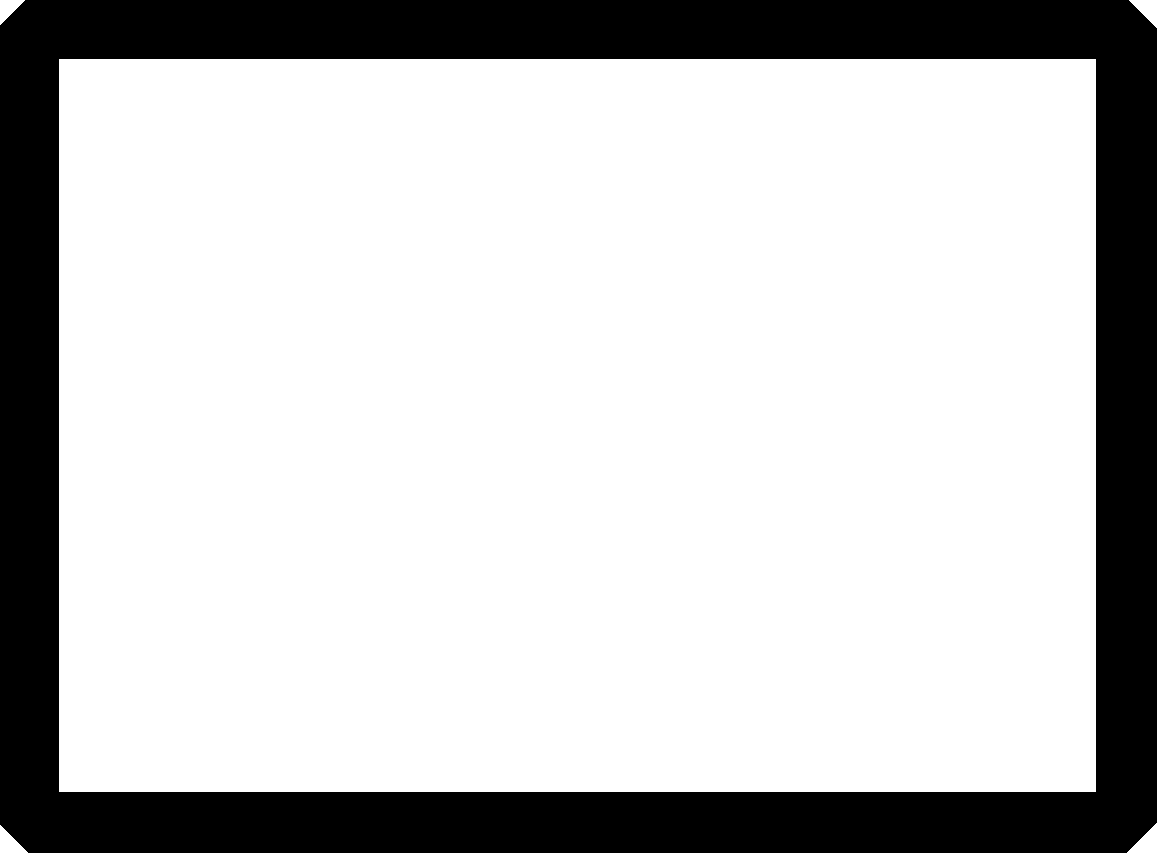 | 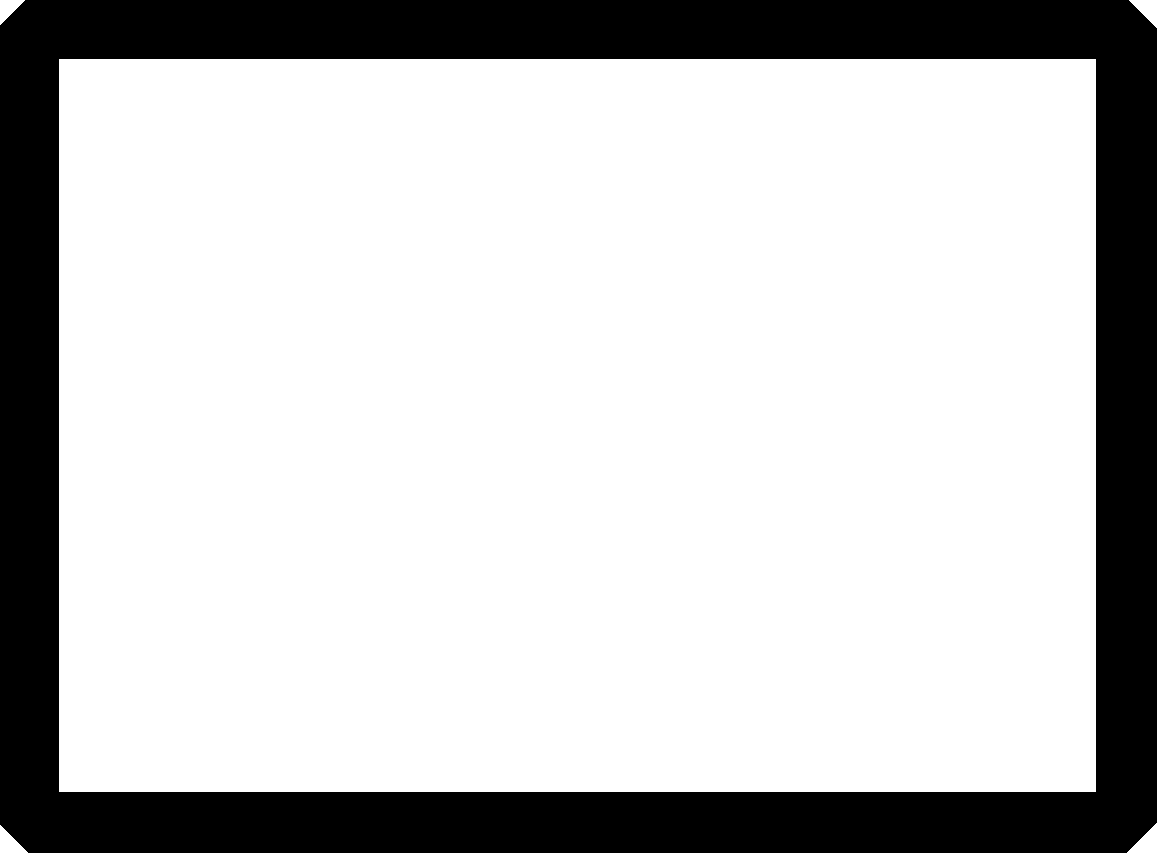 | 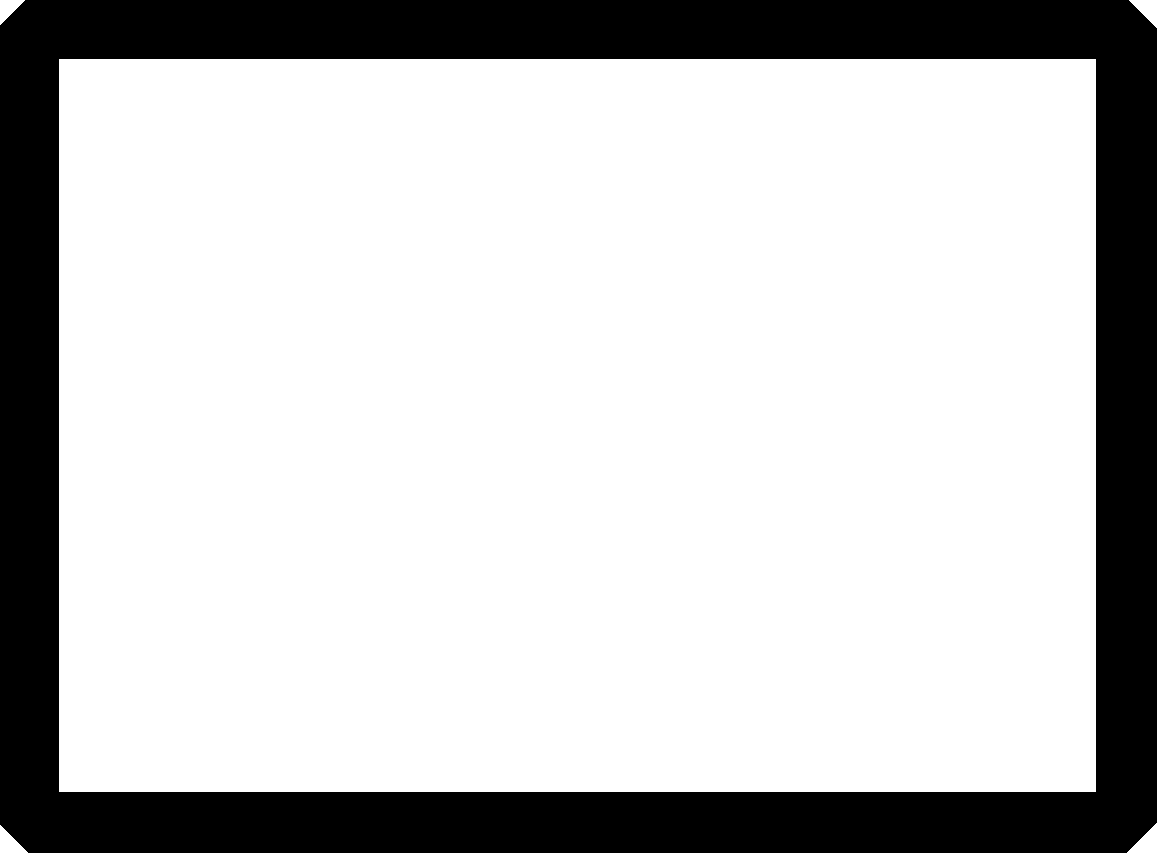 | 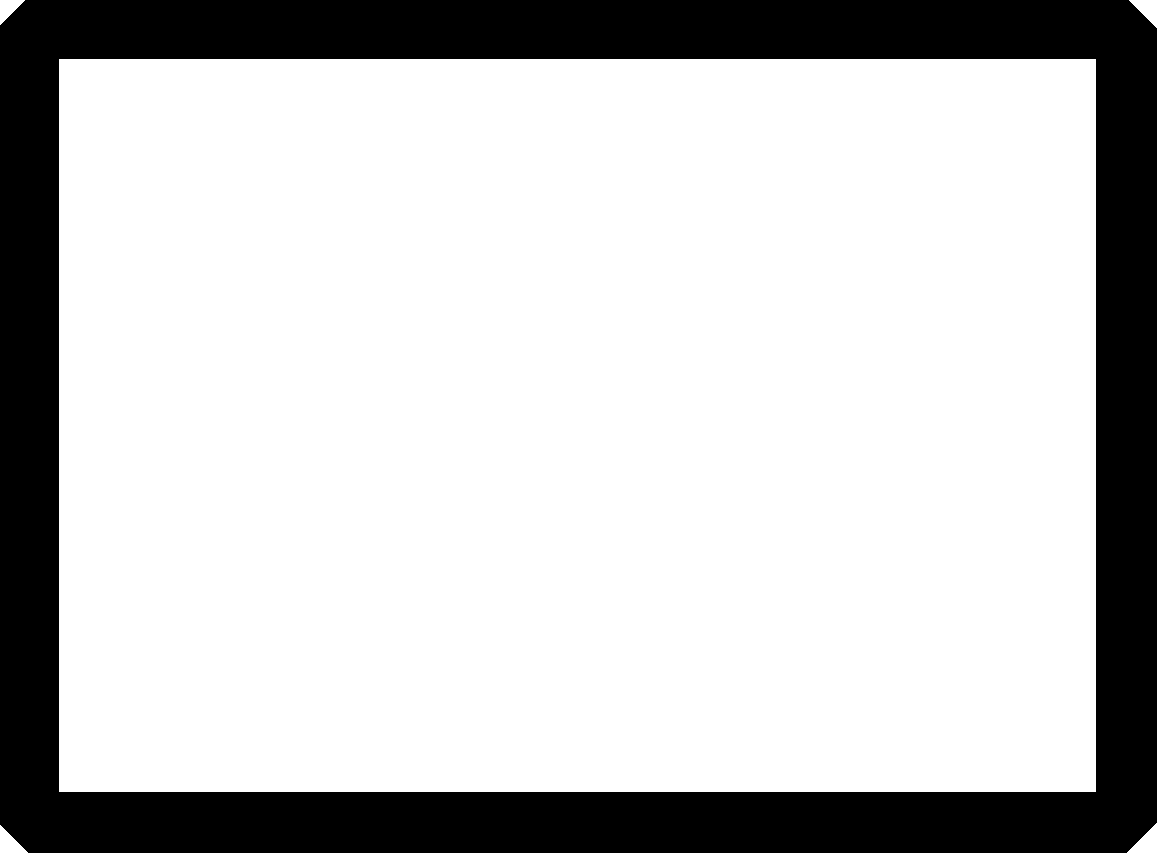 | 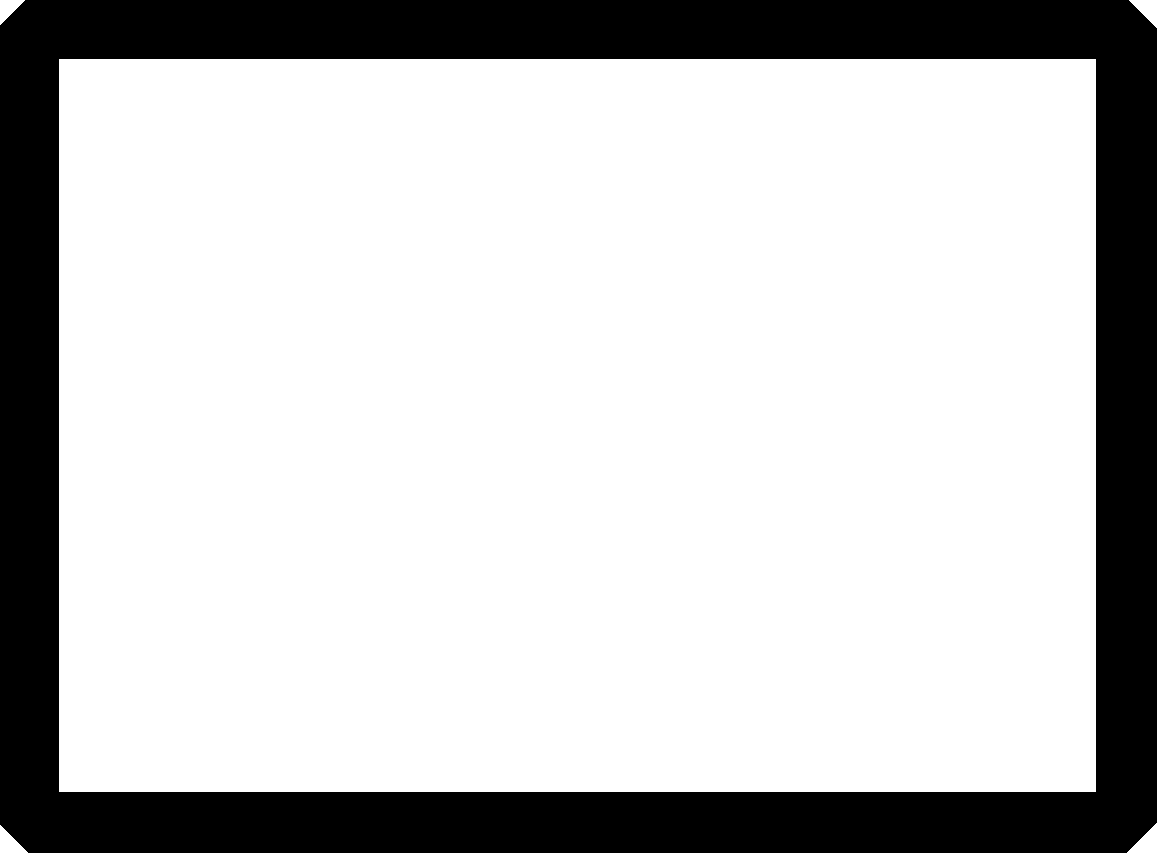 | 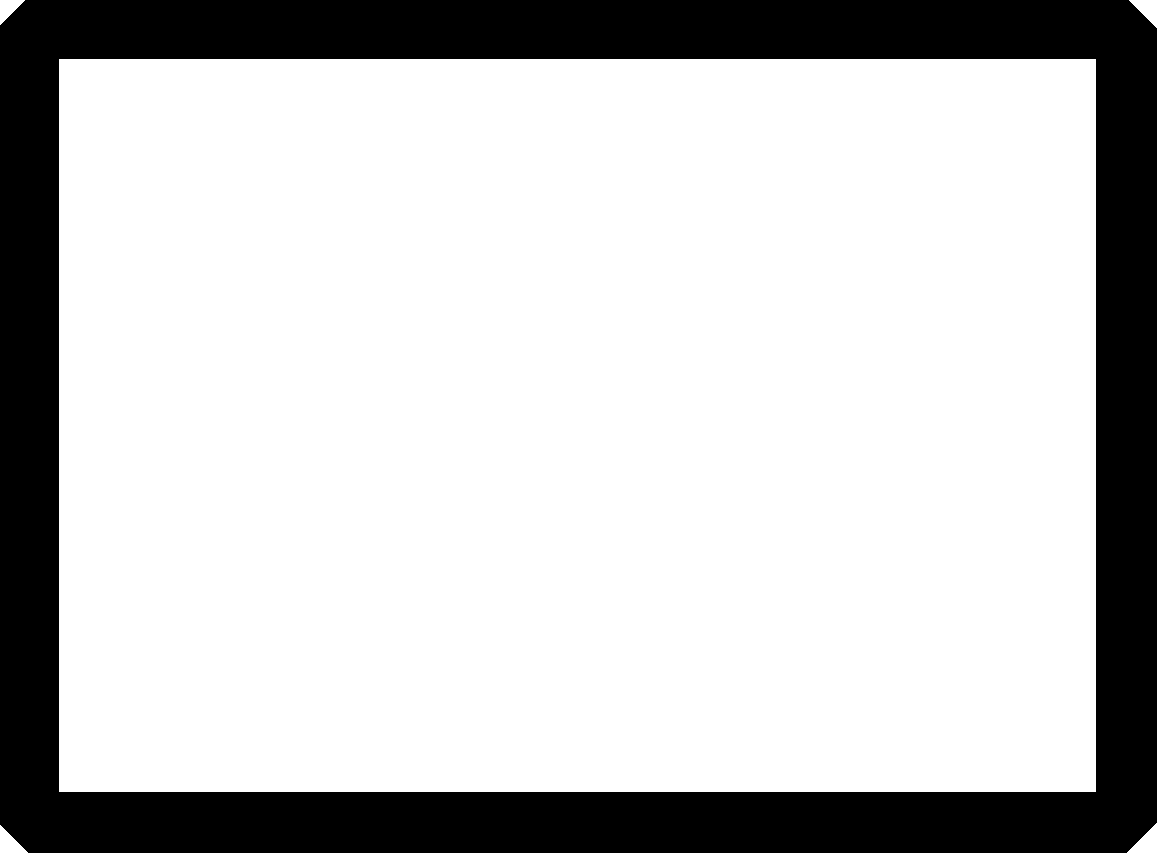 | 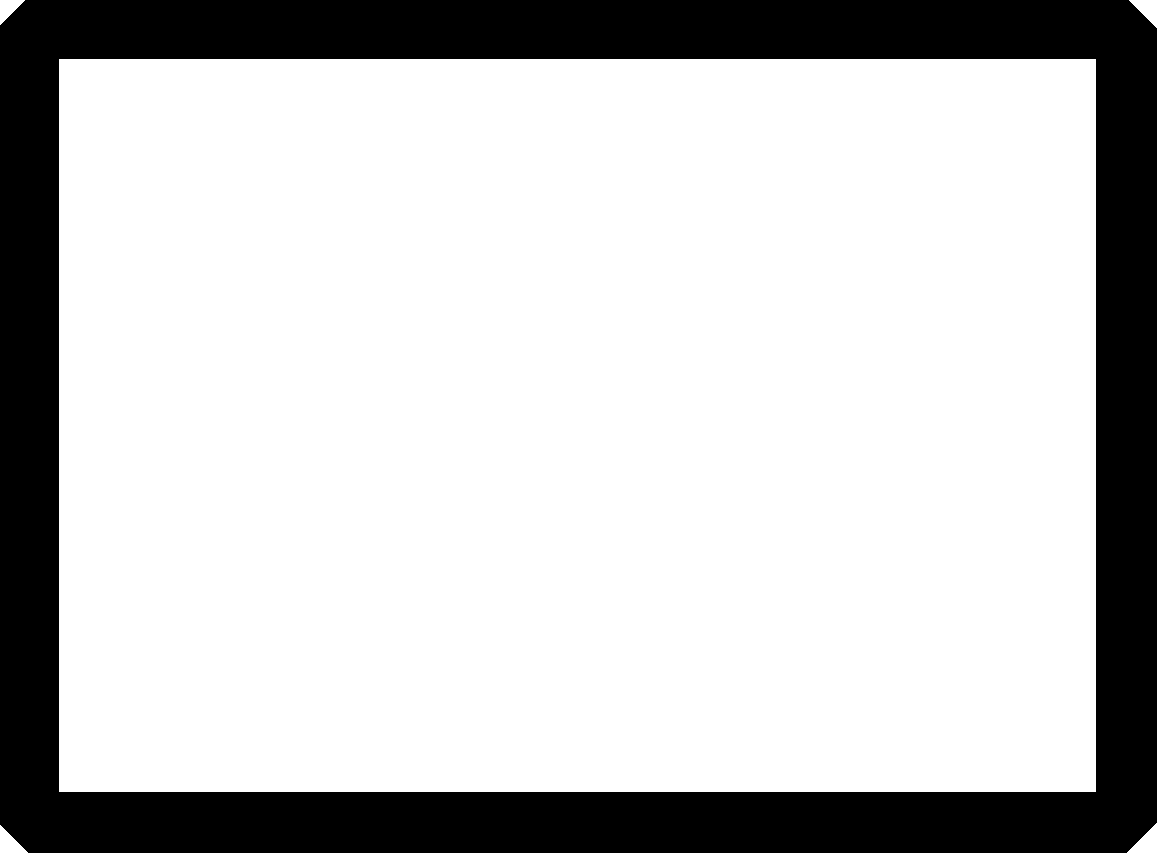 |
| **B2g.** Establish a forum with the patient/caregiver. |  |  |  |  |  |  |  |

**B3.** Digital skills in Content Creation. To what extent is training in the following digital content creation skills necessary for cancer care? Please indicate your opinion on a scale from 1 to 7 (1 being *Absolutely not necessary*and 7 being *Absolutely necessary*).

| **Absolutely not necessary**  **[1]** | **Absolutely not necessary**  **[1]** | **[2]** | **[3]** | **[4]** | **[5]** | **[6]** | **Absolutely necessary**  **[7]** |
| --- | --- | --- | --- | --- | --- | --- | --- |
| **B3a.** Produce simple digital content (e.g., text, tables, images, audio files) in at least one format using digital tools. |  |  |  |  |  |  |  |
| **B3b.** Perform basic editing of content produced by others. |  |  |  |  |  |  |  |
| **B3c.** Distinguish what content is protected by copyright |  |  |  |  |  |  |  |
| **B3d.** Apply and modify simple functions and settings of programmes and applications (e.g., change the default). |  |  |  |  |  |  |  |

**B4.** Digital skills in Safety. To what extent is training in the following digital safety skills necessary for cancer care? Please indicate your opinion on a scale from 1 to 7 (1 being Absolutely not necessary and 7 being Absolutely necessary).

|  | **Absolutely not necessary**  **[1]** | **[2]** | **[3]** | **[4]** | **[5]** | **[6]** | **Absolutely necessary**  **[7]** |
| --- | --- | --- | --- | --- | --- | --- | --- |
| **B4a.** Know how to take basic measures to protect devices (e.g., use antivirus and secure passwords, two-step verification). |  |  |  |  |  |  |  |
| **B4b.** Know what to do if your credentials (username and password) or any other confidential information are stolen. |  |  |  |  |  |  |  |
| **B4c.** Know what kind of information should not be shared on the Internet. |  |  |  |  |  |  |  |
| **B4d.** Manage the risk arising from the use of digital health technologies. |  |  |  |  |  |  |  |
| **B4e.** Recognise bad practices in the use of eHealth. |  |  |  |  |  |  |  |
| **B4f.** Identify whether a particular digital solution may be harmful to a patient/caregiver. |  |  |  |  |  |  |  |

**B5.** Digital skills in e-Health Problem Solving. To what extent is training in the following digital problem solving skills necessary for cancer care? Please indicate your opinion on a scale from 1 to 7 (1 being *Absolutely not necessary* and 7 being *Absolutely necessary*).

|  | **Absolutely not necessary**  **[1]** | **[2]** | **[3]** | **[4]** | **[5]** | **[6]** | **Absolutely necessary**  **[7]** |
| --- | --- | --- | --- | --- | --- | --- | --- |
| **B5a.** Know how to find support and assistance when a technical problem arises or when using a new device, programme or application. |  |  |  |  |  |  |  |
| **B5b.** Know how to solve some routine problems (e.g., close program, restart computer, reinstall/update program, check Internet connection). |  |  |  |  |  |  |  |
| **B5c.** Know that digital tools can help solve problems, but be aware of their limitations. |  |  |  |  |  |  |  |
| **B5d.** Know that when faced with a technological or non-technological problem, known digital tools can be used to solve it. |  |  |  |  |  |  |  |

**B6.** Digital skills in Ethics. To what extent is training in the following digital ethics skills necessary for cancer care? Please indicate your opinion on a scale from 1 to 7 (1 being *Absolutely not necessary* and 7 being *Absolutely necessary*).

|  | **Absolutely not necessary**  **[1]** | **[2]** | **[3]** | **[4]** | **[5]** | **[6]** | **Absolutely necessary**  **[7]** |
| --- | --- | --- | --- | --- | --- | --- | --- |
| **B6a.** Transformation of your health data (e.g., anonymisation/pseudo-anonymisation) for secondary use of health data (e.g., processing health data to evaluate the cost-effectiveness of a service). |  |  |  |  |  |  |  |
| **B6b.** Use digital health tools in accordance with the organisation's policies, regulations and current legislation at local, national and international level. |  |  |  |  |  |  |  |
| **B6c.** Identify conflicts of interest arising from the use of eHealth technologies. |  |  |  |  |  |  |  |
| **B6d.** Act in accordance with protocols and good clinical practice guidelines. |  |  |  |  |  |  |  |

**B7.** Digital skills in Patient Empowerment. To what extent is training in the following digital patient empowerment skills necessary for cancer care? Please indicate your opinion on a scale from 1 to 7 (1 being Absolutely not necessary and 7 being Absolutely necessary).

|  | **Absolutely not necessary**  **[1]** | **[2]** | **[3]** | **[4]** | **[5]** | **[6]** | **Absolutely necessary**  **[7]** |
| --- | --- | --- | --- | --- | --- | --- | --- |
| **B7a.** Skills and knowledge to make shared decision-making (benefits, risks, alternatives, doing nothing). |  |  |  |  |  |  |  |
| **B7b.** Use digital health technologies according to user needs, available resources and context. |  |  |  |  |  |  |  |
| **B7c.** Use of digital health solutions to improve access and equity of care, access, and equity of care. |  |  |  |  |  |  |  |
| **B7d.** Fit to digital patient/caregiver preferences. |  |  |  |  |  |  |  |
| **B7e.** Identify, advise, and support patient/caregiver regarding values and moral problems. |  |  |  |  |  |  |  |
| **B7f.** Inform and facilitate legal procedures affecting patient/caregiver's care (informed consent, participation in clinical trials, advance directives, etc.). |  |  |  |  |  |  |  |

**C.** Please rate from 1 to 7 (1 being *Very Bad* and 7 being *Very good*) the performance of your co-workers in each digital skill for cancer care.

|  | **Very bad [1]** | **[2]** | **[3]** | **[4]** | **[5]** | **[6]** | **Very good [7]** |
| --- | --- | --- | --- | --- | --- | --- | --- |
| **C1.** Digital Information skills |  |  |  |  |  |  |  |
| **C2.** Digital Communication skills |  |  |  |  |  |  |  |
| **C3.** Digital Content Creation skills |  |  |  |  |  |  |  |
| **C4.** Digital Safety skills |  |  |  |  |  |  |  |
| **C5.** Digital Problem-Solving skills |  |  |  |  |  |  |  |
| **C6.** Digital Ethics skills |  |  |  |  |  |  |  |
| **C7.** Digital Patient Empowerment skills |  |  |  |  |  |  |  |

**D.** Please indicate from 1 to 7 (1 being Not at all important and 7 being Highly important) the importance of each digital skill for cancer care.

|  | **Not important [1]** | **[2]** | **[3]** | **[4]** | **[5]** | **[6]** | **Highly important [7]** |
| --- | --- | --- | --- | --- | --- | --- | --- |
| **D1.** Digital Information skills |  |  |  |  |  |  |  |
| **D2.** Digital Communication skills |  |  |  |  |  |  |  |
| **D3.** Digital Content Creation skills |  |  |  |  |  |  |  |
| **D4.** Digital Safety skills |  |  |  |  |  |  |  |
| **D5.** Digital Problem-Solving skills |  |  |  |  |  |  |  |
| **D6.** Digital Ethics skills |  |  |  |  |  |  |  |
| **D7.** Digital Patient Empowerment skills |  |  |  |  |  |  |  |

**THANK YOU VERY MUCH FOR PARTICIPATING IN THIS SURVEY**

PC Survey

We would like to invite you to participate in a survey for the research activity carried out in the framework of the EU-funded TRANSiTION project (id: 101101261), which started in 2023 and will continue until 2025.

**WHAT IS THE PROJECT ABOUT?**

It aims to provide digital skills training for healthcare workers, focusing on the needs of clinical professionals, non-clinical professionals, patients and their informal carers for cancer prevention, diagnosis, treatment, and survivorship. The proposal aims to improve the digital competences of healthcare professionals and support their safe and effective use of existing digital tools, while increasing their readiness and willingness to adopt new ones. The training is expected to improve and re-skill healthcare staff in terms of use of digital solutions and digital readiness, but also to support a faster transition towards more efficient, cost-effective, and patient-centred healthcare models.

Clinical professionals and non-clinical professionals must increasingly combine their knowledge of clinical practice with their knowledge of technology and change management to ensure that digital technology fits the reality of care practice, ensure buy-in from frontline healthcare workers, and manage the cultural change needed to drive learning in healthcare organisations.

**PURPOSE OF THE SURVEY**

This survey is part of WP3 of the TRANSiTION project and aims to address the current training needs and knowledge of healthcare workers, non-healthcare workers, cancer patients and their caregivers. Your participation is particularly important to us, as it will provide us with essential information to guide the training programmes that will be developed in WP4.

**WHAT DO YOU NEED TO KNOW?**

We remind you that the answers shared are **CONFIDENTIAL** and will be anonymised in the elaboration of deliverables and scientific contributions. Your collaboration is **VOLUNTARY**. There are no right or wrong answers, better or worse. What we are interested in is the perspective and opinion of all those involved. All information provided will only be used as described in the project purpose.

As this is an international project, you should be aware that by agreeing to participate in the study, you are also giving your consent that the information you provide may be transferred to another country as part of the research collaboration and its publication. Of course, the project coordinator will ensure the security of your personal data in compliance with current EU legislation in terms of data protection.

**APPROVAL**

The project has been reviewed by the Pontevedra-Vigo-Ourense Research Ethics Committee (ref: 2023/309). In accordance with the General Data Protection Regulation, the WP3 Coordinator, Servizo Galego de Saúde (SERGAS), is responsible for ensuring that the processing of your personal health data has a lawful basis.

**WITHDRAW PARTICIPATION**

You can withdraw your consent at any time and without any reason. If you have any questions about the project, you can contact the WP3 coordinators.

**BLOCK I: CONSENT TO PARTICIPATE**

If you wish to participate, you must sign the declaration of consent below.

I declare that I am aware of the terms of this informed consent, the aims of the research, the forms of participation, the costs and risks involved, as well as access to information and the safeguarding of information produced in the study. I acknowledge that the information I provide during this research is strictly confidential and anonymous. Furthermore, it will be used for scientific dissemination purposes only. I have been informed that I may ask questions about the project at any time and that I may withdraw from the project at any time, without having to give explanations or suffer any consequences for such a decision.

**I agree to participate in this survey.**

**□ YES □ NO**

**BLOCK II:** **SOCIODEMOGRAPHIC VARIABLES**

To begin with, we would like you to answer the following questions.

**A1. Gender:**

| Male | Female | Another |
| --- | --- | --- |
|  |  |  |

**A2. Age group:**

| 18-30 years | 31-45 years | 46-60 years | 61 years or more |
| --- | --- | --- | --- |
|  |  |  |  |
| **A3. Country of residence: ____________________________________________** |  |  |  |

| **A4.** How large is the population of your municipality/city where you usually live? |
| --- |
| <50,000 population |
| 50,001-100,000 population |
| >50,000 population |

**A5. Group:**

| Patient | Caregiver |
| --- | --- |
|  |  |

| **A6.** What type of ownership is the health system you usually use? |
| --- |
| Public owned |
| Privately owned – subsidized |
| Privately owned – not subsidized |
| I don't know |

| **A7.**  Have you received prior training in digital competencies/skills? |
| --- |
| Yes |
| No |

**BLOCK III: TRAINING NEEDS**

**B1.** Digital skills in Information. In your health system, to what extent is training in the following digital information skills necessary for your cancer care/the care of the person you are caring for? Please indicate your opinion on a scale from 1 to 7 (1 being *Absolutely not necessary* and 7 being *Absolutely necessary*).

|  | **Absolutely not necessary**  **[1]** | **[2]** | **[3]** | **[4]** | **[5]** | **[6]** | **Absolutely necessary**  **[7]** |
| --- | --- | --- | --- | --- | --- | --- | --- |
| **B1a.** Search for information on the Internet using a search engine (e.g., Google, Bing, Yahoo!...). |  |  |  |  |  |  |  |
| **B1b.** Differentiate between reliable and unreliable online information. |  |  |  |  |  |  |  |
| **B1c.** Save or store files or content (e.g., text, images, music, videos, web pages) and retrieve them once saved or stored. |  |  |  |  |  |  |  |
| **B1d.**Access to your health data (electronic medical record). |  |  |  |  |  |  |  |
| **B1e.** Exchange health data through electronic systems. |  |  |  |  |  |  |  |

**B2.** Digital skills in Communication. In your health system, to what extent is training in the following digital communication skills necessary for your cancer care/the care of the person you are caring for?

Please indicate your opinion on a scale from 1 to 7 (1 being *Absolutely not necessary* and 7 being *Absolutely necessary*).

|  | **Absolutely not necessary**  **[1]** | **[2]** | **[3]** | **[4]** | **[5]** | **[6]** | **Absolutely necessary**  **[7]** |
| --- | --- | --- | --- | --- | --- | --- | --- |
| **B2a.** Communicate with others using mobile phone, voice over IP (e.g., Skype), email or chat, using basic functions (e.g., voice messaging, SMS, sending and receiving email, text exchange). |  |  |  |  |  |  |  |
| **B2b.** Share files and content with simple tools. |  |  |  |  |  |  |  |
| **B2c.** Use digital technologies to interact with services (e.g., governments, banks, hospitals). |  |  |  |  |  |  |  |
| **B2d.** Use social networking and online collaboration tools. |  |  |  |  |  |  |  |
| **B2e.**Use digital communication standards (e.g., liking, retweeting, sharing a story, sending a DM). |  |  |  |  |  |  |  |
| **B2f.**Communicate statistical information tailored to you/your caregiver’s knowledge. |  |  |  |  |  |  |  |
| **B2g.**Implement a medical web forum between health professionals and you/your carers. |  |  |  |  |  |  |  |

**B3.** Digital skills in Content Creation. In your health system, to what extent is training in the following digital content creation skills necessary for your cancer care/the care of the person you are caring for? Please indicate your opinion on a scale from 1 to 7 (1 being *Absolutely not necessary* and 7 being *Absolutely necessary*).

| **Absolutely not necessary**  **[1]** | **Absolutely not necessary**  **[1]** | **[2]** | **[3]** | **[4]** | **[5]** | **[6]** | **Absolutely necessary**  **[7]** |
| --- | --- | --- | --- | --- | --- | --- | --- |
| **B3a.** Produce simple digital content (e.g., text, tables, images, audio files) in at least one format using digital tools. |  |  |  |  |  |  |  |
| **B3b.** Perform basic editing of content produced by others. |  |  |  |  |  |  |  |
| **B3c.** Distinguish what content is protected by copyright |  |  |  |  |  |  |  |
| **B3d.** Apply and modify simple functions and settings of programmes and applications (e.g., change the default). |  |  |  |  |  |  |  |

**B4.** Digital skills in Safety. In your health system, to what extent is training in the following digital safety skills necessary for your cancer care/the care of the person you are caring for? Please indicate your opinion on a scale from 1 to 7 (1 being *Absolutely not necessary* and 7 being *Absolutely necessary*).

|  | **Absolutely not necessary**  **[1]** | **[2]** | **[3]** | **[4]** | **[5]** | **[6]** | **Absolutely necessary**  **[7]** |
| --- | --- | --- | --- | --- | --- | --- | --- |
| **B4a.** Know how to take basic measures to protect devices (e.g., use antivirus and secure passwords, two-step verification). |  |  |  |  |  |  |  |
| **B4b.** Know what to do if your credentials (username and password) or any other confidential information are stolen. |  |  |  |  |  |  |  |
| **B4c.** Know what kind of information should not be shared on the Internet. |  |  |  |  |  |  |  |
| **B4d.** Manage the risk arising from the use of digital health technologies. |  |  |  |  |  |  |  |
| **B4e.** Recognise bad practices in the use of eHealth. |  |  |  |  |  |  |  |
| **B4f.** Identify whether a particular digital solution may be harmful to a patient/caregiver. |  |  |  |  |  |  |  |

**B5.** Digital skills in e-Health Problem Solving. In your health system, to what extent is training in the following digital e-Health problem solving skills necessary for your cancer care/the care of the person you are caring for? Please indicate your opinion on a scale from 1 to 7 (1 being *Absolutely not necessary* and 7 being *Absolutely necessary*).

|  | **Absolutely not necessary**  **[1]** | **[2]** | **[3]** | **[4]** | **[5]** | **[6]** | **Absolutely necessary**  **[7]** |
| --- | --- | --- | --- | --- | --- | --- | --- |
| **B5a.** Know how to find support and assistance when a technical problem arises or when using a new device, programme or application. |  |  |  |  |  |  |  |
| **B5b.** Know how to solve some routine problems (e.g., close program, restart computer, reinstall/update program, check Internet connection). |  |  |  |  |  |  |  |
| **B5c.** Know that digital tools can help solve problems, but be aware of their limitations. |  |  |  |  |  |  |  |
| **B5e.** Know that when faced with a technological or non-technological problem, known digital tools can be used to solve it. |  |  |  |  |  |  |  |

**B6.** Digital skills in Ethics. In your health system, to what extent is training in the following digital ethics skills necessary for your cancer care/the care of the person you are caring for? Please indicate your opinion on a scale from 1 to 7 (1 being *Absolutely not necessary* and 7 being *Absolutely necessary*).

|  | **Absolutely not necessary**  **[1]** | **[2]** | **[3]** | **[4]** | **[5]** | **[6]** | **Absolutely necessary**  **[7]** |
| --- | --- | --- | --- | --- | --- | --- | --- |
| **B6a.** Transformation of your health data (e.g., anonymisation/pseudo-anonymisation) for secondary use of health data (e.g., processing health data to evaluate the cost-effectiveness of a service). |  |  |  |  |  |  |  |
| **B6b.** Use digital health tools in accordance with the organisation's policies, regulations and current legislation at local, national and international level. |  |  |  |  |  |  |  |
| **B6c.** Identify conflicts of interest arising from the use of eHealth technologies. |  |  |  |  |  |  |  |
| **B6d.** Act in accordance with protocols and good clinical practice guidelines. |  |  |  |  |  |  |  |

**B7.** Digital skills in Patient Empowerment. In your health system, to what extent is training in the following digital patient empowerment skills necessary for your cancer care/the care of the person you are caring for? Please indicate your opinion on a scale from 1 to 7 (1 being Absolutely not necessary and 7 being Absolutely necessary).

|  | **Absolutely not necessary**  **[1]** | **[2]** | **[3]** | **[4]** | **[5]** | **[6]** | **Absolutely necessary**  **[7]** |
| --- | --- | --- | --- | --- | --- | --- | --- |
| **B7a.** Skills and knowledge to make shared decision-making (benefits, risks, alternatives, doing nothing). |  |  |  |  |  |  |  |
| **B7b.** Use digital health technologies according to your needs, available resources, and context. |  |  |  |  |  |  |  |
| **B7c.** Use of digital health solutions to improve access and equity of care, access, and equity of care. |  |  |  |  |  |  |  |
| **B7d.** Fit to your/your caregiver’s digital competencies. |  |  |  |  |  |  |  |
| **B7e.** Identify, advise, and support you/your caregivers regarding values and moral problems. |  |  |  |  |  |  |  |
| **B7f.** Inform and facilitate legal procedures affecting your care (informed consent, participation in clinical trials, advance directives, etc.). |  |  |  |  |  |  |  |

**C.** Please rate from 1 to 7 (1 being *Very Bad* and 7 being *Very good*) the performance of the professionals at your health centre in each digital skill.

|  | **Very bad [1]** | **[2]** | **[3]** | **[4]** | **[5]** | **[6]** | **Very good [7]** |
| --- | --- | --- | --- | --- | --- | --- | --- |
| **C1.** Digital Information skills |  |  |  |  |  |  |  |
| **C2.** Digital Communication skills |  |  |  |  |  |  |  |
| **C3.** Digital Content Creation skills |  |  |  |  |  |  |  |
| **C4.** Digital Safety skills |  |  |  |  |  |  |  |
| **C5.** Digital Problem-Solving skills |  |  |  |  |  |  |  |
| **C6.** Digital Ethics skills |  |  |  |  |  |  |  |
| **C7.** Digital Patient Empowerment skills |  |  |  |  |  |  |  |

**D.** Please indicate from 1 to 7 (1 being Not at all important and 7 being Highly important) the importance of each digital skill for your cancer care/ the cancer care of the patient.

|  | **Not important [1]** | **[2]** | **[3]** | **[4]** | **[5]** | **[6]** | **Highly important [7]** |
| --- | --- | --- | --- | --- | --- | --- | --- |
| **D1.** Digital Information skills |  |  |  |  |  |  |  |
| **D2.** Digital Communication skills |  |  |  |  |  |  |  |
| **D3.** Digital Content Creation skills |  |  |  |  |  |  |  |
| **D4.** Digital Safety skills |  |  |  |  |  |  |  |
| **D5.** Digital Problem-Solving skills |  |  |  |  |  |  |  |
| **D6.** Digital Ethics skills |  |  |  |  |  |  |  |
| **D7.** Digital Patient Empowerment skills |  |  |  |  |  |  |  |

**THANK YOU VERY MUCH FOR PARTICIPATING IN THIS SURVEY**
