# Supplementary material for: Training Gaps in Digital Skills for the Cancer Health Care Workforce Based on Insights From Clinical Professionals, Nonclinical Professionals, and Patients and Caregivers: Qualitative Study
Source: JMIR Med Educ. 2025 Oct 8;11:e78490. doi: 10.2196/78490 (PMC12547342; doi:10.2196/78490)
Supplement: Multimedia Appendix 2 [file mededu_v11i1e78490_app2.docx]

Appendix II: Additional analysis of training needs

*Table 1.  Training needs in digital information skills.*

| Item | CP  M (SD) | NCP  M (SD) | PC  M (SD) | H | *p* |
| --- | --- | --- | --- | --- | --- |
| **B1a.** Search for information on the Internet using a search engine (e.g., Google, Bing, Yahoo!...). | 6.03 (1.72) | 5.75 (1.57) | 5.15 (1.57) | 5.32 | 0.070 |
| **B1b.** Differentiate between reliable and unreliable online information. | 6.16 (1.70) | 6.25 (1.06) | 6.31 (1.44) | 0.52 | 0.772 |
| **B1c.** Save or store files or content (e.g., text, images, music, videos, web pages) and retrieve them once saved or stored. | 5.76 (1.65) | 5.38 (1.31) | 5.08 (1.32) | 4.77 | 0.092 |
| **B1d.** Use the Electronic Health Record for the storage of health data. | 6.24 (1.48) | 6 (1.51) | 5.77 (1.74) | 1.10 | 0.576 |
| **B1e.** Identify the advantages, disadvantages, risks, and opportunities of data exchange through electronic systems. | 6.29 (1.23) | 6.06 (1.06) | 5.69 (1.32) | 3.84 | 0.146 |

*Note. CP: clinical professionals; NCP: non-clinical professionals; PC: patients/caregivers; H: Kruskal-Wallis test statistic*

*Table 2.  Training needs in communication skills.*

| Item | CP  M (SD) | NCP  M (SD) | PC  M (SD) | H | *p* |
| --- | --- | --- | --- | --- | --- |
| **B2a.** Communicate with others using mobile phone, voice over IP (e.g., Skype), email or chat, using basic functions (e.g., voice messaging, SMS, sending and receiving email, text exchange). | 5.63 (1.82) | 5.81 (1.05) | 5.15 (1.62) | 1.84 | 0.398 |
| **B2b.** Share files and content with simple tools. | 5.87 (1.60) | 5.44 (1.03) | 5.08 (1.66) | 5.43 | 0.066 |
| **B2c.** Use digital technologies to interact with services (e.g., governments, banks, hospitals). | 5.95 (1.54) | 5.56 (1.09) | 5.54 (1.27) | 4.35 | 0.114 |
| **B2d.** Use social networking and online collaboration tools. | 5.58 (1.64) | 5.25 (1.29) | 4.54 (1.61) | 5.66 | 0.059 |
| **B2e.** Use digital communication standards (e.g., liking, retweeting, sharing a story, sending a DM). | 4.76 (1.75) | 5.06 (1.48) | 4.62 (1.66) | 0.62 | 0.735 |
| **B2f.** Communicate statistical information according to the characteristics of the audience. | 5.71 (1.63) | 5.75 (1.18) | 4.85 (1.95) | 2.40 | 0.301 |
| **B2g.** Establish a forum with the patient/caregiver. | 5.61 (1.57) | 6.06 (1.12) | 5 (1.91) | 2.02 | 0.364 |

*Note. CP: clinical professionals; NCP: non-clinical professionals; PC: patients/caregivers; H: Kruskal-Wallis test statistic*

*Table 3.  Training needs in digital content creation skills*

| Item | CP  M (SD) | NCP  M (SD) | PC  M (SD) | H | *p* |
| --- | --- | --- | --- | --- | --- |
| **B3a.** Produce simple digital content (e.g., text, tables, images, audio files) in at least one format using digital tools. | 5.89 (1.56) | 5.44 (1.26) | 4.77 (1.96) | 5.22 | 0.074 |
| **B3b.**  Perform basic editing of content produced by others. | 5.84 (1.48) | 5.06 (1.24) | 4.46 (1.94) | 8.92 | 0.012 |
| **B3c.** Distinguish what content is protected by copyright | 5.84 (1.28) | 5.31 (1.35) | 4.77 (2.09) | 3.26 | 0.196 |
| **B3d.** Apply and modify simple functions and settings of programmes and applications (e.g., change the default). | 5.92 (1.42) | 5.19 (1.33) | 4.54 (1.76) | 9.08 | 0.011 |

*Note. CP: clinical professionals; NCP: non-clinical professionals; PC: patients/caregivers; H: Kruskal-Wallis test statistic*

*Table 4.  Training needs in digital safety skills*

| Item | CP  M (SD) | NCP  M (SD) | PC  M (SD) | H | *p* |
| --- | --- | --- | --- | --- | --- |
| **B4a.** Know how to take basic measures to protect devices (e.g., use antivirus and secure passwords, two-step verification). | 6.29 (1.43) | 5.94 (1.24) | 5.92 (1.60) | 2.45 | 0.294 |
| **B4b.** Know what to do if your credentials (username and password) or any other confidential information are stolen. | 6.50 (1.15) | 6.25 (0.86) | 6.15 (1.52) | 3.80 | 0.150 |
| **B4c.** Know what kind of information should not be shared on the Internet. | 6.53 (1.06) | 6.06 (0.99) | 6 (1.47) | 5.93 | 0.052 |
| **B4d.** Manage the risk arising from the use of digital health technologies. | 6.34 (1.47) | 5.94 (0.99) | 5.62 (1.71) | 3.64 | 0.162 |
| **B4e.** Recognise bad practices in the use of eHealth. | 6.47 (1.03) | 6.13 (0.88) | 5.62 (1.76) | 4.59 | 0.101 |
| **B4f.** Identify whether a particular digital solution may be harmful to a patient/caregiver. | 6.53 (0.98) | 6.19 (1.17) | 5.62 (1.76) | 3.65 | 0.161 |

*Note. CP: clinical professionals; NCP: non-clinical professionals; PC: patients/caregivers; H: Kruskal-Wallis test statistic*

*Table 5.  Training needs in digital e-Health problem solving skills*

| Item | CP  M (SD) | NCP  M (SD) | PC  M (SD) | H | *p* |
| --- | --- | --- | --- | --- | --- |
| **B5a.** Know how to find support and assistance when a technical problem arises or when using a new device, programme or application. | 6.39 (1.15) | 6 (1.09) | 5.31 (1.80) | 6.44 | 0,040 |
| **B5b.** Know how to solve some routine problems (e.g., close program, restart computer, reinstall/update program, check Internet connection). | 6.11 (1.43) | 6 (0.97) | 5.54 (1.45) | 2.63 | 0,268 |
| **B5c.** Know that digital tools can help solve problems, but be aware of their limitations. | 6.11 (1.37) | 5.63 (0.88) | 5.46 (1.45) | 6,55 | 0,038 |
| **B5d.** Use the Electronic Health Record for the storage of health data. | 5.92 (1.32) | 5.81 (1.05) | 5.08 (1.75) | 2,74 | 0,254 |

*Note. CP: clinical professionals; NCP: non-clinical professionals; PC: patients/caregivers; H: Kruskal-Wallis test statistic*

*Table 6.  Training needs in digital ethics skills*

| Item | CP  M (SD) | NCP  M (SD) | PC  M (SD) | H | *p* |
| --- | --- | --- | --- | --- | --- |
| **B6a.** Transformation of your health data (e.g., anonymisation/pseudo-anonymisation) for secondary use of health data (e.g., processing health data to evaluate the cost-effectiveness of a service). | 6.03 (1.51) | 6 (1.16) | 4.46 (1.76) | 9,77 | 0,008 |
| **B6b.** Use digital health tools in accordance with the organisation's policies, regulations and current legislation at local, national and international level. | 6.16 (1.50) | 5.81 (1.05) | 4.38 (1.76) | 14,09 | 0,001 |
| **B6c.** Identify conflicts of interest arising from the use of eHealth technologies. | 6.13 (1.47) | 5.56 (0.73) | 4.77 (1.69) | 14,19 | 0,001 |
| **B6d.** Act in accordance with protocols and good clinical practice guidelines. | 6.47 (1.35) | 6.25 (1) | 5.15 (1.95) | 7,11 | 0,029 |

*Note. CP: clinical professionals; NCP: non-clinical professionals; PC: patients/caregivers; H: Kruskal-Wallis test statistic*

*Table 7.  Training needs in digital patient empowerment skills*

| Item | CP  M (SD) | NCP  M (SD) | PC  M (SD) | H | *p* |
| --- | --- | --- | --- | --- | --- |
| **B7a.** Skills and knowledge to make shared decision-making (benefits, risks, alternatives, doing nothing). | 6.26 (1.41) | 5.88 (1.02) | 5.08 (1.80) | 7,58 | 0,023 |
| **B7b.** Use digital health technologies according to user needs, available resources and context. | 6.13 (1.51) | 5.44 (0.96) | 5.38 (1.76) | 9,22 | 0,010 |
| **B7c.** Use of digital health solutions to improve access and equity of care, access, and equity of care. | 6.16 (1.46) | 5.69 (1.20) | 5.54 (1.71) | 4,84 | 0,089 |
| **B7d.** Fit to digital patient/caregiver preferences. | 6 (1.51) | 5.75 (1) | 5.46 (1.85) | 2,37 | 0,306 |
| **B7e.** Identify, advise, and support patient/caregiver regarding values and moral problems. | 6.08 (1.46) | 5.75 (0.77) | 5.15 (1.91) | 5,75 | 0,057 |
| **B7f.** Inform and facilitate legal procedures affecting patient/caregiver's care (informed consent, participation in clinical trials, advance directives, etc.). | 6.37 (1.08) | 5.94 (0.85) | 5.23 (1.74) | 8,26 | 0,016 |

*Note. CP: clinical professionals; NCP: non-clinical professionals; PC: patients/caregivers; H: Kruskal-Wallis test statistic*
